# Helping hands, flourishing hearts: A meta‐analytic study of organizational citizenship behaviors and subjective well‐being

**DOI:** 10.1111/aphw.70158

**Published:** 2026-05-15

**Authors:** Christopher W. Wiese, Victoria S. Scotney, Daphne X. Hou, Louis Tay

**Affiliations:** ^1^ Georgia Institute of Technology Atlanta GA USA; ^2^ Purdue University West Lafayette USA; ^3^ University of South Florida Tampa USA

**Keywords:** life satisfaction, meta‐analysis, negative affect, organizational citizenship behaviors, positive affect, subjective well‐being

## Abstract

Does going the extra mile at work enhance an employee's well‐being, or does it come with hidden costs? With these two diverging perspectives on the impact of Organizational Citizenship Behaviors (OCBs) on employee well‐being and inconclusive previous meta‐analytic findings, the current study provides a much‐needed update. We conducted a meta‐analysis (*k* = 186, *n* = 29,034) on the relationship between OCBs and Subjective Well‐Being (positive affect, negative affect, life satisfaction) to provide updated estimates of the cross‐sectional effects and the first meta‐analytic evidence of longitudinal and within‐person effects. We also explore critical moderators (age, gender, affective context (work vs. general), and OCB type (individual vs. organizational)). Cross‐sectional findings reveal significant associations between OCBs and positive affect (ρ = .34), negative affect (ρ = −.11), and life satisfaction (ρ = .30). Notably, only negative affect correlates differently with different OCB types. Consistent with research on gender role expectations, females experience lower life satisfaction from OCBs than males. Longitudinal findings show that positive and negative affect predict future OCBs; however, limited studies examine the reverse causal direction. On a daily level, positive affect is associated with higher levels of OCBs, whereas negative affect is not associated with OCBs. When examining specific OCB types, negative affect is associated with greater engagement in OCB‐Is. An important implication is the need to foster employee well‐being to sustain prosocial behaviors at work. We discuss further implications for both theory and practice, highlighting the need for a more nuanced understanding of helping behaviors at work.

## INTRODUCTION

Every day, employees go out of their way to help their coworkers, volunteer for additional tasks, and contribute to a positive work environment. These actions, known as Organizational Citizenship Behaviors (OCBs), include altruism, civic virtue, and other discretionary acts that transcend formal job requirements (Podsakoff et al., 2000). Extensive research has established that OCBs are positively related to organizational outcomes, such as performance and effectiveness (Organ & Ryan, 1995; Podsakoff et al., 2000; Podsakoff et al., 2009, 2014). While the benefits of OCBs for organizational‐centric outcomes are well documented, their implications for employee well‐being have received less systematic attention and are consequently less well understood. Do these laudable acts relate to greater employee well‐being, or are they also associated with personal costs?

Over the last quarter century, a burgeoning body of research has started to unveil the potential “dark side” of OCBs, suggesting that they might not always result in favorable outcomes for employees (Bergeron, 2007; Bergeron et al., 2013; Bolino et al., 2013). Concerns have been raised regarding the possibility that the discretionary and often unrecognized nature of OCBs leads to work overload, stress, and a blurring of work‐life boundaries, ultimately impacting employee well‐being negatively. Additionally, the literature points to potential inequities in the benefits of OCBs related to gender (Allen, 2006; Kidder, 2002) and age (Huang et al., 2015; Pletzer, 2021; Profili et al., 2016). For instance, women may experience fewer well‐being benefits from OCBs due to gendered expectations that OCBs are expected rather than exceptional, unlike their male counterparts, who often receive more praise. Likewise, there may be differences in age, as older individuals might find more happiness in engaging in OCBs, reflecting a value shift toward altruism with aging. Furthermore, research has shown that the consequences for well‐being associated with OCBs may vary when analyzed at different levels (between‐ vs. within‐person) or by the type of OCB (Halbesleben & Wheeler, 2011; Koopman et al., 2016). Collectively, the literature paints a complex picture in which the relationship between OCBs and well‐being may vary depending on employee characteristics, OCB type, and measurement context.

Crucially, earlier meta‐analytic studies of this relationship (e.g., Kaplan et al., 2009; Organ & Ryan, 1995; Shockley et al., 2012; summarized in Table 1) could not fully explore these complexities and nuances, as the body of literature had not yet matured sufficiently. This research gap is particularly significant, given that employee well‐being has emerged as a key indicator of organizational success (Tay et al., 2023). Accordingly, the goal of the current meta‐analysis is not to definitively adjudicate whether OCBs benefit or harm employee well‐being, but rather to (a) provide an updated synthesis of the cross‐sectional associations between OCBs and both affective (positive and negative affect) and cognitive (life satisfaction) indicators of well‐being; (b) compare how individual‐focused (OCB‐Is) versus organization‐focused (OCB‐Os) behaviors relate to these SWB components; and (c) provide the first meta‐analytic estimates of the longitudinal and within‐person effects between these constructs to better understand their dynamic and temporal nature. By doing so, we identify where the evidence is robust and where further primary research is required to resolve remaining inconclusive findings.

**TABLE 1:** Effect sizes from previous meta‐analyses.

Study	SWB	OCB	*k*	*r*	ρ	Note
Organ & Ryan, 1995	PA	OCB‐O	6	.06	.07	OCB dimensions were altruism (OCB‐I) and compliance (OCB‐O) NA included measures of neuroticism, trait anxiety, and impatience‐irritability PA included measures of extraversion
	PA	OCB‐I	7	.12	.15	
	NA	OCB‐O	5	−.09	−.12	
	NA	OCB‐I	6	−.05	−.06	
Dalal, 2005	PA	OCB	23	.28	.34	Only included studies that examined a relationship between OCBs and CWBs (focus of this meta‐analysis)
	NA	OCB	23	−.08	−.10	
Kaplan et al., 2009	Trait PA	OCB	7	.19	.23	More restrictive on inclusion/exclusion criteria for positive/negative affect Only included affect measured “in general,” or a time period of six months or more
	Trait NA	OCB	11	−.08	−.10	
Shockley et al., 2012	State PA	OCB	24	.29	.32	Only state affect, which included any measure from the present up to one week in the past
	State NA	OCB	17	−.01	−.02	
Geiger et al., 2019	NA	OCB	70	−.11	−.13	Negative affect measured with no reference to time or situation was coded as trait; if measured with reference to a specific time or situation it was coded as state
	Trait NA	OCB	32	−.16	−.18	
	State NA	OCB	44	−.09	−.10	
	NA	OCB‐O	27	−.17	−.20	
	NA	OCB‐I	36	−.08	−.14	
	NA	OCB‐composite	33	−.11	−.14	
	Trait NA	OCB‐O	11	−.30	−.33	
	State NA	OCB‐O	18	−.11	−.12	
	Trait NA	OCB‐I	9	−.16	−.19	
	State NA	OCB‐I	29	−.05	−.06	
Chiaburu et al., 2022	PA	OCB‐O	9	.15	.17	Only included non‐self‐report measures of OCBs Excluded ESM studies Included both state and trait measures of affect (i.e., different time frames)
	PA	OCB‐I	12	.15	.17	
	PA	OCB‐CH	28	.24	.27	
	NA	OCB‐O	6	−.15	−.17	
	NA	OCB‐I	7	−.08	−.09	
	NA	OCB‐CH	23	−.09	−.11	

*Note*: SWB = Subjective Well‐Being; OCB = Organizational Citizenship Behavior; *k* = Number of independent samples; *r* = Observed correlation coefficient; ρ = Corrected correlation coefficient.

The current meta‐analytic investigation leverages the development of the OCB literature to examine these subtleties and makes significant contributions to the literature. First, we take a comprehensive look at the relationship between OCBs and both affective (positive, negative affect) and cognitive (life satisfaction) indicators of well‐being. This examination not only provides an update on previous work on affective well‐being (e.g., Kaplan et al., 2009; Organ & Ryan, 1995; Shockley et al., 2012), but also extends our understanding by examining cognitive well‐being. Moreover, we explore how individual‐focused (OCB‐Is) and organization‐focused OCBs (OCB‐Os) may relate differently to affective and cognitive well‐being. Previous meta‐analyses comparing OCB‐I and OCB‐O have not included positive affect (Geiger et al., 2019) or have only included studies with other‐reported measures of OCB, restricting the number of included studies (Chiaburu et al., 2022). Understanding the relationships between different types of OCBs and well‐being is crucial because the well‐being payoffs for OCB‐Is, which benefit specific individuals (e.g., helping behavior), may differ significantly from those of OCB‐Os, which are directed toward the organization (e.g., civic virtue) as a whole. Furthermore, our investigation into life satisfaction is particularly important as it broadens the potential benefits of OCBs beyond the immediate work domain, highlighting how these behaviors could enrich employees' overall quality of life and satisfaction, thereby contributing to a more comprehensive understanding of the far‐reaching impacts of OCBs.

Second, this study provides initial meta‐analytic evidence of the longitudinal relationship between OCBs and employee well‐being, marking an important contribution by illustrating how these behaviors influence well‐being over time, and vice versa. This longitudinal perspective is essential for elucidating the enduring effects of OCBs on well‐being, which can support integrating time into future theoretical models that have largely been built on cross‐sectional data. Relatedly, the current work also provides preliminary meta‐analytic evidence of the within‐person effects of OCBs and well‐being. The development of the OCB literature since previous meta‐analyses (e.g., Shockley et al., 2012) allows us to provide the first meta‐analytic estimates of longitudinal and within‐person effects on well‐being. These results may reveal a differential impact of the “dark side” of OCBs, supporting the assertions of scholars in this field (Bergeron, 2007; Bergeron et al., 2013).

Lastly, the current efforts seek to demonstrate how the OCB–well‐being relationship varies by the context of well‐being (work or general affect), gender, and age, which addresses critical gaps in the literature, as we currently do not understand whether these effects vary as a function of these potential moderators. These results will help enrich academic dialogue by emphasizing the need for inclusive, context‐sensitive research that captures the complexity of OCBs' effects across different demographic and situational boundaries.

In the following sections, we develop our theoretical framework by contrasting the “bright” and “dark” sides of OCBs through the lenses of COR and SDT. We first examine how OCBs relate to the three components of Subjective Well‐Being (SWB; i.e., positive affect, negative affect, life satisfaction; Diener et al., 1999). Our analysis then distinguishes between individual‐focused (OCB‐I) and organization‐focused (OCB‐O) behaviors to identify potential differences in their well‐being associations. Furthermore, we extend beyond cross‐sectional patterns to explore the enduring nature of these effects through longitudinal and within‐person analyses. Finally, we investigate gender, age, and measurement context as critical moderators of the OCB–well‐being linkage before discussing the implications of our findings for theory and organizational practice.

## THEORETICAL DEVELOPMENT AND BACKGROUND

Since Organ (1988) first defined Organizational Citizenship Behaviors (OCBs) as discretionary acts that contribute to organizational effectiveness without formal reward, research has robustly linked these behaviors to enhanced productivity and positive job attitudes. While early work focused on specific dimensions like altruism or courtesy, contemporary research typically organizes them into two broader categories based on the intended beneficiary of the behavior. Following Williams and Anderson's (1991) influential taxonomy, OCBs are commonly distinguished as OCB‐I, which encompasses behaviors directed toward benefiting specific individuals (e.g., assisting an overloaded coworker, orienting new employees), and OCB‐O, which captures impersonal behaviors aimed at supporting the organization more broadly (e.g., adhering to rules beyond minimum expectations, participating in non‐required organizational activities). These two forms have been shown empirically to be distinct constructs, and this distinction has become foundational in subsequent theoretical and empirical work on OCBs.

More recently, research has provided ample evidence that OCBs have positive relations with organizational outcomes, linking them with organizational‐focused outcomes (e.g., individual, group, and organizational effectiveness and productivity, absenteeism) as well as employee attitudes toward the job (e.g., job satisfaction, turnover intentions; N. P. Podsakoff et al., 2009; Podsakoff et al., 2000; Spitzmuller et al., 2008). Over time, however, the field began to broaden its lens. A growing person‐centric movement (Tay et al., 2023; Weiss & Rupp, 2011; Woo et al., 2018) pushed scholars to consider not only how OCBs advance organizational goals but also what they mean for the individuals who perform them (Bergeron, 2007; Penner et al., 2005). This shift reflects increasing recognition that citizenship behaviors lie outside formal job requirements, draw on employees' finite personal resources, and therefore may carry mixed implications for well‐being (e.g., Baranik & Eby, 2016; Bolino et al., 2013; Koopman et al., 2016). As this literature developed, researchers began questioning whether the benefits that OCBs reliably confer at the organizational level translate to personal outcomes. This has produced two contrasting perspectives: a “bright side,” which highlights gains such as enhanced job satisfaction, positive affect, and a sense of accomplishment, and a “dark side,” which emphasizes the potential for strain, resource depletion, and role overload when employees engage in sustained citizenship behavior. Together, these viewpoints underscore the multifaceted nature of OCBs and the need to understand their consequences not only for organizations but for the individuals who enact them.

Before turning to the theoretical perspectives that inform our predictions, it is important to clarify how we approach the relationship between OCBs and well‐being for most of the manuscript. Although these constructs may influence one another over time, our theoretical arguments in the following sections focus on their association rather than on any specific causal ordering. That is, when we describe how OCBs may relate to well‐being, or how well‐being may relate to OCBs, these mechanisms are intended to illustrate the processes that could underlie their linkage in either direction. These perspectives, therefore, inform expectations about the strength of the OCB‐well‐being relationship across conditions, not its temporal precedence. However, we rely on direction‐specific reasoning in our later discussion of longitudinal effects, where specific temporal pathways are explicitly considered. Against this backdrop, it is critical to understand the broader work on the link between helping and happiness.

### Helping and happiness

A wide breadth of conceptual frameworks has been used to explain why helping is often associated with happiness. One perspective argues that receiving well‐being benefits from helping out may be a hardwired characteristic of human nature. Evolutionary theories provide a foundational perspective, suggesting that prosocial behaviors, such as OCBs, are intrinsically rewarding because they played a role in our evolutionary past. Actions that benefit others can elicit neurobiological rewards, as shown by research indicating that helping others activates the brain's reward centers (Harbaugh et al., 2007; Moll et al., 2006). This phenomenon is partially explained by inclusive fitness theory, which posits that behaviors promoting the survival of one's genes can be inherently satisfying (Hamilton, 1964). Extending this, multilevel‐selection theory asserts that groups with altruistic members are more likely to survive than those with selfish individuals, emphasizing the adaptive value of helping behaviors (Sober & Wilson, 1998; Wilson, 1997).

Within organizational science, two primary theoretical frameworks—Conservation of Resources (COR) theory (Hobfoll, 1989; Hobfoll et al., 2018) and Self‐Determination Theory (SDT; Deci et al., 2017; Ryan & Deci, 2000) – provide complementary accounts of how OCBs relate to employee happiness. COR theory posits that people are motivated to secure and preserve valued resources (e.g., time, energy, and knowledge) and to build additional resources whenever possible (Hobfoll, 1989; Hobfoll et al., 2018). Engaging in OCBs requires a discretionary investment of these finite resources (Bolino et al., 2015; Halbesleben & Wheeler, 2011). This reflects an underlying “resource calculus”: employees typically help others to prevent further losses, restore what has been depleted, or create opportunities for future dividends, such as reciprocal support or social capital. However, this investment carries inherent risk; if the resources devoted to helping are not met with adequate returns, the resulting imbalance can strain or diminish well‐being.

Supplementing this resource view, SDT argues that human thriving depends on the satisfaction of three Basic Psychological Needs (BPNs): autonomy (feeling in control), competence (feeling capable), and relatedness (feeling connected) (Deci et al., 2017; Lavelle et al., 2007). When OCBs are performed, they create pathways for resource gains (e.g., trust, appreciation, and strengthened interpersonal bonds), which directly support the fulfillment of these core needs (Aknin et al., 2013; Dunn et al., 2014). This alignment between helping behaviors and fundamental psychological needs is what ultimately translates prosocial acts into enhanced psychological happiness. Together, COR and SDT reveal that helping has the potential to satisfy core psychological needs while simultaneously drawing on finite personal resources. This duality provides a useful foundation for understanding why scholars have argued that OCBs may, at times, enhance well‐being and, at other times, impose significant costs.

### The bright and dark sides of OCBs

Scholars use the tenets of COR and SDT to explain the “bright” and “dark” sides of OCBs. Briefly, from the “bright side” perspective, engaging in OCBs can generate meaningful returns on resource investments. OCBs generate meaningful returns on resource investments. For example, helping a coworker can foster reciprocal exchanges that provide instrumental benefits like social support, favorable performance evaluations, and career opportunities (e.g., stronger social support, favorable performance evaluations, career opportunities; Bergeron et al., 2013; Kidder, 2002; Podsakoff et al., 2009), which replenish resources and satisfy key psychological needs. Beyond these instrumental returns, OCBs may also provide emotional benefits: helping can redirect attention away from negative affect and elicit feelings of purpose, connection, and gratification (e.g., Glomb et al., 2011; Thompson et al., 1980).

Conversely, the dark‐side perspective emphasizes that OCBs require investments beyond formal requirements, which can lead to resource depletion. Bergeron (2007); Bergeron et al. (2013) argue that because individuals have finite resources, excessive citizenship behavior can detract from core task performance, leading to a lack of organizational recognition and missed career opportunities, key indicators of career success and high well‐being. Beyond simple resource trade‐offs, OCBs may be driven by social pressures or felt obligations rather than genuine altruism. This pressure can result in role overload, work–family conflict, and stress, ultimately undermining employee well‐being (e.g., Bolino et al., 2013). These two opposing perspectives can be used to understand how OCBs relate to various aspects of the employee's mental health and well‐being (e.g., job satisfaction, burnout). In the current efforts, we focus on a traditional conceptualization of well‐being that puts the psychological health of the employee front and center: Subjective Well‐Being.

### Subjective well‐being

To synthesize and clarify these potential effects, the current effort focuses on the tripartite perspective of Subjective Well‐Being (SWB; Diener et al., 1999), a widely recognized conceptualization of employee well‐being. Historically, the organizational sciences have favored an “organization‐first” perspective (Tay et al., 2023), emphasizing outcomes like job satisfaction and engagement that capture how individuals evaluate their jobs rather than their lives. While informative, such indicators can be overly context‐specific and may obscure the broader consequences of discretionary behaviors on employees as whole persons. Leveraging the SWB framework allows for a “person‐first” philosophy that views employees as individuals whose wellness encompasses domains beyond the workplace. Furthermore, this approach provides a cleaner test of these relationships by avoiding the conceptual confounding often found between OCBs and traditional job attitudes (e.g., engagement, job satisfaction), which are heavily intertwined with organizational outcomes.

SWB encompasses three key components: positive affect, negative affect, and life satisfaction. *Positive affect* and *negative affect* are the presence of pleasant and unpleasant moods and emotions, respectively, reflecting individuals' emotional responses to their life circumstances (Diener et al., 1999). *Life satisfaction* can be considered a cognitive judgment of one's life, where individuals assess the quality of their lives based on their own chosen criteria. Understanding these three components of subjective well‐being is crucial for exploring the relationship between well‐being and OCBs, as it provides insight into how these voluntary behaviors can not only influence affective states but also an individual's overarching evaluation of their life satisfaction.

### Subjective well‐being and organizational citizenship behaviors

While the bright and dark sides of OCBs offer competing predictions about how OCB should be related to SWB, previous meta‐analytic evidence tends to align more closely with the bright‐side perspective, which we argue currently. As noted earlier, COR and SDT both provide clear pathways for understanding why each of the three components of SWB will be related to OCBs. For positive affect, COR suggests that helping can generate meaningful resource gains that enhance positive emotional states. SDT similarly proposes that engaging in OCBs promotes positive affect by satisfying core psychological needs, especially relatedness and competence.

The theoretical picture for negative affect is more nuanced, but still compatible with the bright‐side arguments. COR argues that resource investment is not only a means of gaining resources but also a strategy for protecting against future losses and recovering from prior depletion. OCBs can serve this function by fostering supportive relationships, strengthening social bonds, and reducing interpersonal strain, each of which may buffer against and decrease negative affect. Thus, both COR and SDT predict that OCBs can reduce negative affect by building resources and fulfilling psychological needs, albeit to a lesser degree than positive affect.

Meta‐analytic evidence generally supports these expectations (see Table 1). Three decades ago, Organ and Ryan (1995) conducted the first meta‐analysis on the subject, with relatively few studies (*k* = 5–7), and found small effect sizes between positive and negative affect and between OCB‐Is (i.e., Altruism) and OCB‐Os (i.e., Compliance). Ten years later, the research had significantly expanded (k = 23), allowing Dalal (2005) to reveal that positive affect was more strongly associated with OCBs than negative affect, but still observed a modest negative relationship between negative affect and citizenship behavior. While the main purpose of Kaplan et al.’s (2009) meta‐analysis was to examine the relationship between trait PA/NA and job performance, they also reported similar effect sizes as Dalal (2005). Shockley et al. (2012) expanded on this work by examining state affect, demonstrating a significant relationship between state positive affect and OCBs but a non‐significant relationship between state negative affect and OCBs, contrasting previous findings. However, Geiger et al. (2019) provided the most comprehensive examination of negative affect to date, finding much larger effect sizes than previous research. Most recently, Chiaburu et al. (2022) focused exclusively on non‐self‐reported measures, finding small to modest effect sizes between PA/NA and OCBs. Collectively, we propose that OCBs will be positively associated with positive affect and negatively associated with negative affect.

Life satisfaction has not received the same degree of empirical attention as affective well‐being in the OCB literature, and no prior meta‐analysis has focused on this association specifically. Even so, the theoretical logic underlying the bright‐side perspective suggests that OCBs should be positively related to life satisfaction. SDT provides a clear foundation for this expectation: helping behaviors can enhance an individual's sense of autonomy, competence, and relatedness, which are strongly tied to global evaluations of one's life (Ryan & Deci, 2000). This theoretical reasoning is consistent with broader prosociality research, which has repeatedly demonstrated positive associations between helping behaviors and life satisfaction across diverse samples (e.g., Dulin et al., 2001; Harlow & Cantor, 1996; Hidalgo et al., 2013; Zuffianò et al., 2018) and a recent meta‐analysis that found a positive relationship between prosociality and a composite of many forms of well‐being, which included life satisfaction (Hui et al., 2020). Taken together, these arguments lead us to expect a positive relationship between OCBs and life satisfaction.

Hypothesis 1 There will be a significant relationship between OCBs and (a) positive affect (+), (b) negative affect (−), and (c) life satisfaction (+).

### Individual vs. organizations OCBs

The expansion of the literature allows us to take a comprehensive examination of a critical distinction in OCBs. As noted earlier, OCBs are commonly classified into two predominant types: OCB‐Individual (OCB‐Is) and OCB‐Organization (OCB‐Os; Spitzmuller et al., 2008). The distinction between OCB‐I and OCB‐Os is rooted in their target beneficiaries – OCB‐Is directly benefit specific individuals within the organization, such as through acts of helping coworkers with tasks, while OCB‐Os benefit the organization as a whole, through actions like adhering to company policies when unsupervised. Importantly, most of the research that uses different frameworks (N. P. Podsakoff et al., 2014; Podsakoff et al., 2000) can be subsumed under this simplified categorization. This is especially helpful for the purposes of a meta‐analysis, as it allows extant research to be easily integrated and summarized. Consequently, this parsimonious approach also facilitates our understanding of how the three dimensions of SWB relate to these two different types of OCBs.

Considering the previous arguments, there is no reason to expect a change in the direction of the effect size between SWB and OCB‐Is and OCB‐Os. However, it is possible that there will be differences in magnitude, with OCB‐Is demonstrating a stronger relationship with SWB than OCB‐Os. A stronger relationship between well‐being and OCB‐Is than OCB‐Os might be expected because OCB‐Is involve direct, personal interactions that satisfy basic psychological needs, such as relatedness and competence, as outlined in SDT (Ryan & Deci, 2000). Moreover, research on prosocial behavior has suggested that people are more likely to experience increased SWB when they have greater autonomy in helping and feel connected to those they are helping (Aknin et al., 2019). However, this pattern of magnitude may be present only for positive affect and life satisfaction, with the converse true for negative affect. As noted by Geiger et al. (2019), negative emotional experiences in interpersonal contexts (e.g., conflict, frustration, strain with coworkers or supervisors) can generalize and influence broader attitudes and behaviors. When employees feel negatively toward the individuals around them, those emotions may extend beyond the interpersonal domain and shape their overall orientation toward the organization (Lavelle et al., 2007). In this way, negative affect tied to interpersonal experiences may reduce engagement in OCBs that are directed at the organization itself, producing a stronger association between negative affect and OCB‐Os. Hence, we expect that:

Hypothesis 2 There will be a significant association between both OCB‐Is and OCB‐Os with a) positive affect (+), b) negative affect (−), and c) life satisfaction (+).

Hypothesis 3 (a) The relationship between OCB‐Is and positive affect/life satisfaction will be stronger than their relationship with OCB‐Os, while b) the relationship between OCB‐Os and negative affect will exceed that with OCB‐Is.

### Longitudinal effects

One of the unanswered questions within the organizational sciences is whether happy people engage in more OCBs or whether OCBs promote happiness. Much of the existing evidence is cross‐sectional, leaving open the question of whether these associations generalize to longitudinal patterns or reflect causal processes. Moreover, it is this same cross‐sectional evidence that overwhelmingly supports the bright‐side argument. Hence, it is important to revisit the merits of both the bright‐ and dark‐side perspectives when discussing longitudinal effects through the lens of COR and SDT.

From a bright‐side viewpoint, both theories would suggest reasons to expect positive longitudinal effects in both directions, which can be thought of as the SWB➔OCB and OCB➔SWB effects. For the SWB➔OCB effects, COR theory proposes that well‐being can itself be considered a resource (e.g., Hobfoll et al., 2023; Wiese et al., 2025). Individuals with greater well‐being possess more psychological and energetic capacity to invest in approach‐oriented behaviors (Baranik & Eby, 2016; Elliot & Thrash, 2002), such as OCBs. Relatedly, higher well‐being signals a state of relative resource surplus, making discretionary actions like OCBs more feasible and less risky. Evidence from the broader prosociality literature supports this logic, consistently showing that happier individuals engage in more helping behaviors across various contexts (see Aknin et al., 2018; Hui, 2022). Lastly, SDT aligns with this reasoning by suggesting that individuals experiencing need satisfaction (a core component of well‐being) are more intrinsically motivated and socially open (Deci et al., 2017; Ryan & Deci, 2000), which may increase their likelihood of engaging in citizenship behaviors.

For the OCB ➔ SWB pathway, both COR and SDT again point to potential long‐term benefits. COR suggests that helping others can generate valuable resource gains over time, such as stronger interpersonal bonds, increased social support, and a heightened sense of stability in one's social environment (Hobfoll, 1989; Koopman et al., 2016). These resource gains can accumulate and yield growing well‐being “dividends.” SDT complements this by emphasizing that engaging in OCBs can satisfy basic psychological needs, particularly relatedness and competence, fostering a sense of purpose and belonging within the workplace (Deci et al., 2017; Ryan & Deci, 2000). When these needs are met consistently, they contribute to enduring increases in SWB.

However, revisiting the dark‐side arguments suggests that these positive patterns may not persist indefinitely. For the OCB → SWB pathway, COR highlights that sustained helping requires ongoing resource expenditure (Bolino et al., 2004, 2015). If employees repeatedly invest time, energy, and emotional effort into OCBs without receiving sufficient resource gains or need satisfaction in return, a gradual depletion process can emerge. Over time, this imbalance may erode well‐being as employees begin to feel overextended, under‐supported, or obligated rather than autonomous (Bergeron, 2007), conditions that directly undermine SDT's core needs. In this sense, the same behaviors that initially generate well‐being dividends may later contribute to strain or diminished happiness once employees recognize that their efforts are not adequately replenished.

A parallel concern applies to the SWB➔OCB pathway, where high well‐being may directly inhibit discretionary effort through a mood maintenance mechanism. Individuals experiencing high levels of positive affect may become more protective of their current emotional state, leading them to avoid OCBs that are perceived as tedious, high‐effort, or potentially stressful. In this view, instead of well‐being acting as a fuel for helping, it acts as a “status quo” that the employee is unmotivated to disrupt, thereby creating a direct, negative pressure on the likelihood of engaging in extra‐role behaviors.

Given the conflicting perspectives and lack of empirical evidence, we present the following research question to explore the relationship between SWB and OCBs over time.

Research Question 1: What are the longitudinal, reciprocal associations between SWB and OCBs?

### Within‐person effects

The distinction between within‐person and between‐person effects in examining the relationship between affect and OCBs is pivotal for a nuanced understanding of how state affect impacts individuals' work behaviors. Traditional between‐person studies, by aggregating affect and OCBs across individuals, cater well to examining stable associations but fall short in capturing the dynamic, fluctuating nature of affect experienced by individuals on a day‐to‐day basis. This is because the meaning behind the correlation between two variables can fundamentally change when analyzed at different levels. While between‐person analyses might show a certain pattern or strength of relationship across individuals, within‐person analyses could reveal entirely different dynamics for the same variables within the same individuals over time. This discrepancy has practical implications for understanding and influencing work behavior through interventions targeted at affect. Within‐person methodologies (e.g., experience sampling and daily diary studies) address this gap by measuring affective states and corresponding OCBs within the same day, thereby providing insights into the proximal impact of affect on OCBs. Shockley et al. (2012) made an early attempt to coalesce these findings within a meta‐analysis, but had to examine this relationship qualitatively due to an inadequate number of studies in the literature.

The shift toward within‐person studies is increasingly recognized for its ability to uncover the complex dynamics of how daily variations in positive and negative affect influence OCBs (Beal & Gabriel, 2019; Dalal et al., 2009, 2014). We anticipate finding similar patterns at the within‐person level because the core mechanisms of COR and SDT are inherently sensitive to time. Specifically, on days when an employee experiences a momentary surplus of resources (positive affect) or satisfaction of psychological needs, they possess the immediate capacity and motivation to engage in OCBs. Conversely, daily spikes in negative affect signal a momentary resource threat, prompting individuals to conserve energy for core tasks. Because these theoretical frameworks describe processes that respond to shifting internal states, we expect within‐person dynamics to mirror between‐person associations.

Hypothesis 4 There will be a significant association between OCBs and a) positive affect (+) and b) negative affect (−) at the within‐person level of analysis.

### Moderators

As illustrated in Table 1 and corroborated by a review of primary studies, the effect size estimates between OCBs and SWB exhibit considerable variability. This variation suggests that the relationship between these discretionary workplace behaviors and well‐being may depend on various factors. One of the benefits of conducting a meta‐analysis is the ability to examine these potential systematic differences across different studies. In this work, we leverage previous research (e.g., Allen, 2006; Oishi et al., 1999) to consider the degree to which individuals value engaging in OCBs, as well as the surrounding expectations, and how they may play a pivotal role in affecting this relationship. Specifically, we examine the potential moderating effects of Gender, Age, and Context of Measurement. These moderators offer intriguing insights into how personal, situational, and measurement factors may differentially impact the OCB‐SWB relationship, providing a more nuanced understanding of the underlying mechanisms at play.

#### The role of gender

Gender may play an important role in shaping the relationship between OCBs and well‐being (Allen, 2006; Kidder, 2002; Lin, 2008; Ng et al., 2016; Thompson et al., 2020), and it may help explain the variations in effect sizes between OCBs and well‐being. Both COR and SDT emphasize that the benefits of discretionary behaviors depend on the extent to which those behaviors are valued, autonomous, and aligned with individuals' goals and identities. Because OCBs require resource expenditure, the well‐being consequences of engaging in them may differ across genders to the extent that societal gender roles and stereotypes shape expectations about who “should” engage in prosocial or relationally supportive behavior. When OCBs align with the socialized roles of a particular gender (e.g., expectations that women prioritize interpersonal support and relational maintenance), the behavior may feel more identity‐consistent and may yield greater resource gains or need satisfaction. Conversely, when OCBs are experienced as obligatory or taken for granted, the potential well‐being benefits diminish. Prior research indicates that OCBs are often evaluated and rewarded differently across genders, with male‐typed behaviors receiving greater recognition (e.g., Allen, 2006) and women reporting stronger felt obligation to engage in OCBs (P. S. Thompson et al., 2020). Such differences suggest that the same OCB may function as a resource gain for one gender but as a resource drain for another, leading to systematic variation in well‐being outcomes. Hence, we hypothesize that:

Hypothesis 5 The gender composition of studies will moderate the relationship between OCBs and a) positive affect, b) negative affect, and c) life satisfaction, predicting a weaker relationship as the proportion of female participants increases.

#### The role of age

In a related vein, the strength of the relationship between OCBs and SWB could vary with age, which also influences what constitutes a value‐congruent behavior. Socioemotional Selectivity Theory (SST) draws on the underlying tenets of COR and SDT to offer a complementary lifespan perspective on how these processes may change across age (Carstensen, 1992, 2021; Löckenhoff & Carstensen, 2004). SST argues that as people age, they increasingly recognize the finiteness of time and consequently shift their priorities toward emotionally meaningful goals and close, supportive relationships. When viewed through this lens, OCBs may serve as a particularly effective pathway for older workers to satisfy needs for relatedness and competence while accruing valued social and emotional resources. As a result, older employees may both engage more readily in OCBs and derive greater well‐being from them.

In contrast, younger employees often prioritize future‐oriented, instrumental goals (e.g., career advancement, impression management) that do not necessarily align with the intrinsic, need‐satisfying motives emphasized by SDT. Empirical evidence suggests that younger workers may engage in OCBs for these externally driven reasons (Huang et al., 2015; Pletzer, 2021), which can reduce the likelihood that the behavior generates meaningful resource gains or supports genuine need satisfaction. Consequently, OCBs may yield weaker well‐being benefits for younger individuals than for their older counterparts. Hence, we expect that:

Hypothesis 6 The average age of the studies will moderate the relationship between OCBs and a) positive affect, b) negative affect, and c) life satisfaction, such that the relationship becomes stronger as the sample age gets older.

#### The role of context

A final consideration is the extent to which the relationship between OCBs and subjective well‐being depends on the context in which each construct is measured. Specifically, the association may differ depending on whether well‐being is assessed in a work‐specific frame (e.g., work well‐being, job‐related affect) or in a general frame (e.g., life satisfaction, general affect). The compatibility principle (Ajzen & Fishbein, 1970, 1977; Fishbein & Ajzen, 1975) states that relationships between psychological constructs are strongest when the constructs are aligned on the same level of specificity or abstraction. Research consistently demonstrates that mismatched levels of abstraction tend to weaken associations between attitudes, emotions, and behaviors (e.g., Harrison et al., 2006). Applying this logic to the present context, OCBs, behaviors enacted within the work domain, should relate more strongly to work‐specific indicators of well‐being than to global measures that encompass broader life domains. When well‐being and OCBs are assessed within the same contextual frame, their conceptual alignment should yield stronger associations. Specifically, we expect that:

Hypotheses 7 The relationship between OCBs and affective well‐being (i.e., positive and negative affect) will be stronger when measuring work affect as compared to general affect.

## METHOD

**FIGURE 1:**
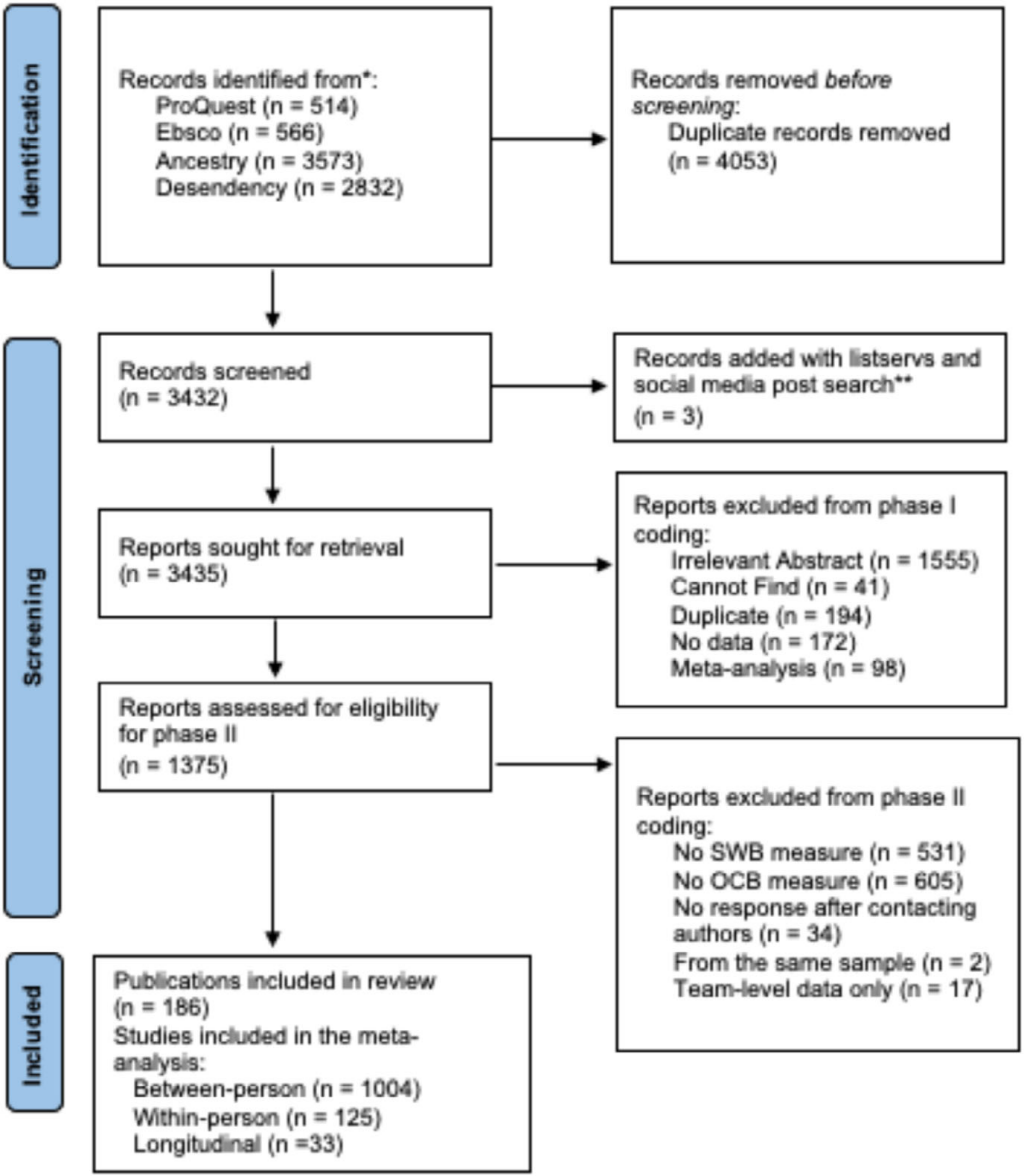
PRISMA flowchart for current meta‐analysis.

To test our hypotheses, we followed the preferred reporting for systematic reviews and meta‐analyses (PRISMA) standards (Page et al., 2021). Data and code used for the analyses can be found on OSF: https://osf.io/uq7jg/?view_only=617163ec6a7c40ddb83cc0a1202eaa27. All online supplemental materials can also be found at this link. See Figure 1 for the complete PRISMA flowchart.

### Literature search

We sought multiple sources to identify all studies, including published journal articles, theses, dissertations, and conference proceedings that have examined the relations between OCB and SWB. Searches were conducted in the following databases: Academic Search Complete, APA PsycArticles, APA PsychInfo, Business Abstracts with Full Text, Business Source Complete, and ProQuest including journals, theses, and dissertations. We applied a combination of keywords as our search term for OCBs (Organizational Citizenship Behavior* OR Conscientious* OR OCB* OR Job Dedication OR Compliance OR Civic Duty OR Personal Initiative OR Prosocial) and SWB (subjective well‐being OR happiness or life satisfaction or positive affect or negative affect OR positive feeling* OR negative feeling* OR PANAS OR SWB).

From these initial results, we selected 16 critical papers (Bolino et al., 2004; Dalal, 2005; Dekas et al., 2013; Erdogan et al., 2012; Kaplan et al., 2009; Lyubomirsky et al., 2005; Ocampo et al., 2018; Organ, 1997; Organ et al., 2005; Organ & Ryan, 1995; Podsakoff et al., 2000; Podsakoff & MacKenzie, 1997; Shockley et al., 2012; Spector & Fox, 2002; Spitzmuller et al., 2008; Van Dyne et al., 1994) that represented either meta‐analyses or reviews on OCBs and reviews on SWB to a secondary ancestry and descendency search. Ancestry and descendency reviews are critical supplemental literature searches in meta‐analyses, which involve cataloging the reference sections of critical articles in the field (Ancestry) and cataloging relevant articles that have cited these critical articles (Descendency). This step in our meta‐analysis is especially important as it helps ensure the comprehensiveness of our effort. In particular to our search, many of our variables (e.g., positive affect, negative affect) are used as control variables in studies. This method ensures that we capture studies that measured both OCBs and SWB. For the Ancestry search, we pulled all of the references of these critical articles into our article pool. For the Descendency search, we searched the articles that cited the previous meta‐analyses and reviews of OCB literature for the keywords: “positive affect,” “negative affect.” or “life satisfaction” in all of the text via both Scopus and Web of Science. For the reviews of the SWB literature, we searched for the term “organizational citizenship behavior” in all of the text. All of these results were placed in our initial article pool.

Our initial search resulted in 7485 articles (ProQuest: 514, Ebsco: 566, Ancestry: 3573, Desendency: 2832); after 4,053 duplicates were identified with reference software, we were left with 3,432 articles for further screening. To further prevent publication bias, we reached out to several listservs (OHPLIST, AOM, RMNET) and posted on social media accounts to ask for unpublished articles. This added three articles to our pool, leaving a final initial article pool of 3,435 to conduct Inclusion/Exclusion Coding on.

### Inclusion criteria and effect size coding

The coding of the articles was conducted by the first three authors and occurred in two phases. In phase one, articles were retained for further review if the abstract was relevant (mentioned OCBs, job performance, or anything within the work domain), the article was not a duplicate of another article, the article contained any quantitative data, and was not a meta‐analysis. All three coders double‐coded a subset of articles (25.25%) to establish a shared mental model (98.26% agreement). After this was established, the remaining articles were independently coded. Phase one coding resulted in articles being excluded if the abstract was not relevant (*n* = 1,555), the article could not be found (*n* = 41), was a duplicate (*n* = 194), had no data (*n* = 172), or was a meta‐analysis (*n* = 98), leaving 1,375 articles for phase two coding.

Phase two coding involved a closer examination of the articles for our inclusion and exclusion criteria and effect size coding of included studies. Specifically, articles were not included for effect size coding if they did not contain both measures of OCBs and SWB, whether it was a duplicate sample from other included samples, or they did not contain effect sizes that could be used in meta‐analytic investigation. All articles in phase two were coded independently by the first two authors, who then met to discuss and resolve any discrepancies. Coding was further validated with an AI coding software for meta‐analysis (Mehr et al., 2026).

We developed a code book that helped define the constructs of interest. We defined OCBs as discretionary behaviors that contribute to organizational functioning. Therefore, we coded measures of behaviors, but excluded measures of intentions, motivations, or values. Importantly, this approach allowed us to include any discretionary behaviors, whether they were an aggregate of multiple dimensions (e.g., a total score from Podsakoff et al.’s, 1990 five‐dimensions), respective to a particular framework (e.g., OCB‐O/OCB‐I vs. five‐dimension approach), or did not fall within a specific framework (e.g., questions concerning helping behavior toward coworkers). Throughout the coding process, we coded effect sizes based on the smallest reported level of abstraction (e.g., civic virtue). For analysis of overall OCBs, we created composite scores of the subscales (e.g., combining OCB‐I and OCB‐O, or the five‐dimensions). This use of composite OCB scores is consistent with common practice in both primary and meta‐analytic studies of OCBs (e.g., Ilies et al., 2009; LePine et al., 2002). For analysis comparing OCB‐I and OCB‐O, we coded based on the target of the behavior – the individual or the organization (Spitzmuller et al., 2008; Williams & Anderson, 1991). We created composite measures of OCB‐Is from measures of Altruism, Courtesy, Peacekeeping, and Cheerleading, and composite measures of OCB‐Os from measures of Conscientiousness, Civic Virtue, and Sportsmanship. Other measures were coded based on the target of the behavior. For example, measures of helping coworkers or supervisors were coded as a measure of OCB‐I, since they are directed toward individuals within the workplace.

Subjective well‐being (SWB) is composed of positive affect (PA), negative affect (NA), and life satisfaction (LS). Positive and negative affect reflect positive and negative emotional experiences, and life satisfaction reflects the cognitive evaluation of one's life (Diener et al., 1999). We included studies that measured at least one of the three dimensions of SWB. However, we excluded studies that only provided a composite of these measures. The majority of LS was measured with the Satisfaction with Life Scale (SWLS; Diener et al., 1985). We included all time frames of affect, such as “at this moment” and “in general.” To maximize similarity between measures, we only included adjective‐based measures of PA and NA. We also included measures of specific affect, like anger and enthusiasm, as long as they were adjective‐based, coding these measures into their broader categories (i.e., NA/PA). For our analysis of work versus non‐work affect, we distinguished whether the measure referred to general experiences of affect or was contextualized to the workplace, specifically measures of affect from work and/or at work. In ESM studies, we coded both between‐ and within‐person correlations.

If the article was missing any information (e.g., if an ESM study did not report both the within‐ and between‐person correlations), we contacted the authors to request the missing information. We contacted 70 authors, and received 26 responses, which allowed us to include five additional studies, as well as six between‐ or within‐person correlations not reported in the original paper for ESM studies. Articles in languages other than English were included with the use of Google Translate, or the assistance of native speakers, to identify relevant information. We excluded articles that did not measure SWBs (*n* = 531) or OCBs (*n* = 605) in ways that matched our definitions, lacked relevant statistics in the paper or author's response (*n* = 34), provided team‐level data (*n* = 17), or came from the same sample (*n* = 2). In total, we included effect sizes from 186 unique samples.

### Meta‐analytic approach

Meta‐analyses were computed following the procedures for random effects meta‐analysis delineated by Schmidt and Hunter (2015), using the *psychmeta* package (Dahlke & Wiernik, 2019). In this approach, we corrected for measurement error using Cronbach's alpha coefficients. In cases where reliability estimates were unavailable, an artifact distribution approach was employed to supplement missing data. For studies reporting multiple effect sizes for a particular variable (e.g., two measures of positive affect), we composited the effect sizes (Schmidt & Hunter, 2015). The specific effect sizes included in these composites were determined based on the analytical goals of each hypothesis being tested. For instance, in exploring Hypothesis 1, a composite effect size representing the relationship between positive affect and OCBs was used when both OCB‐Is and OCB‐Os were reported. Conversely, these effect sizes were not composited for the Hypothesis 2 analyses, as the distinction between OCB‐Is and OCB‐Os was of critical importance.

Importantly, we had three general types of analysis: between‐person, within‐person, and longitudinal analysis.^1^ To assess significant direct effects, we examined the 95% confidence interval (CI) around the corrected effect size (ρ). The CI offers a range of values within which the true population effect size is likely to fall. If the CI includes 0, it suggests there may be no direct relationship between the variables. We also calculated an 80% credibility interval (CV) to indicate the probable distribution of ρ. While the CI estimates sampling error for a single population, the CV represents the distribution and is not influenced by sampling error.

To explore potential moderating effects, we first examined whether the heterogeneity of the direct effect suggested the presence of moderators. Heterogeneity was evaluated using both the Q and *I* ^*2*^ statistics. A Q statistic serves as an indicator of whether there is heterogeneity among meta‐analytic effect sizes, while the *I* ^*2*^ statistic can provide insight into the extent of heterogeneity (Huedo‐Medina et al., 2006). The presence of a significant Q statistic and an *I* ^*2*^ statistic above 75% suggests that moderation hypotheses should be explored. We conducted a meta‐regression (Schmidt, 2017) for continuous moderators (e.g., Age, Gender) and built 90% CIs around the difference in effect sizes at different levels of the moderating variable for categorical moderators using the formula reported in De Jong et al. (2016).

Lastly, to evaluate potential publication bias, p‐hacking, and small study effects, we conducted several sensitivity analyses (cumulative meta‐analysis, PET–PEESE, Trim‐and‐Fill, and one‐study‐removed) for the three focal associations between overall OCBs and life satisfaction, positive affect, and negative affect. A full description of these procedures and results is provided in the online supplementary materials. Across tests, there were some weak indications of bias, particularly for the cross‐sectional and within‐person associations involving negative affect, but these signals were small in magnitude and did not meaningfully alter the interpretation of our main findings.

## RESULTS

### Between‐person analyses

Table 2 includes the cross‐sectional meta‐analytic estimates of the relations between different types of OCBs (i.e., overall OCB, OCB‐I, and OCB‐O) with different aspects of SWB (i.e., positive affect, negative affect, and life satisfaction). We found full support for Hypothesis 1, such that the overall OCBs have significant positive relationships with positive affect (ρ = .34, *k* = 130 95% CI [.31, .37]) and life satisfaction (ρ = .30, *k* = 35, 95% CI [.24, .36]) and a significant negative relationship with negative affect (ρ = −.11, *k* = 122, 95% CI [−.16, −.09]). After breaking down OCBs into OCB‐Is and OCB‐Os, we found support for Hypothesis 2 with significant and positive relationships between OCB‐Os and positive affect (ρ = .32, *k* = 40, 95% CI [.27, .38]) and life satisfaction (ρ = .23, *k* = 9, 95% CI [.11, .35]) as well as OCB‐Is and positive affect (ρ = .30, *k* = 68, 95% CI [.26, .34]) and life satisfaction (ρ = .30, *k* = 12, 95% CI [.16, .44]) and significant and negative relationships between OCB‐Os with negative affect (ρ = −.16, *k* = 34, 95% CI [−.24, −.09]) as well as OCB‐Is and negative affect (ρ = −.09, *k* = 57, 95% CI [−.13, −.05]).

**TABLE 2:** Meta‐analytic effect sizes between subjective well‐being and organizational citizenship behaviors at the between‐person level .

						95% CI		80% CR			
	k	*r*	SD_r_	ρ	SDρ	LL	UL	LL	UL	Q	*I* ^*2*^
	Organizational citizenship behaviors										
Life satisfaction	35	.25	.16	.30	.18	.24	.36	.09	.50	160.69**	78.84
Positive affect	130	.29	.15	.34	.18	.31	.37	.13	.54	591.18**	78.18
*Positive affect (no‐context)*	87	.29	.16	.34	.18	.30	.38	.13	.55	400.77**	78.54
*Positive affect (work context)*	55	.29	.14	.34	.17	.29	.39	.15	.53	201.52**	73.20
Negative affect	122	−.09	.16	−.11	.19	−.14	−.07	−.32	.11	511.47**	76.34
*Negative affect (no‐context)*	89	−.11	.15	−.12	.18	−.16	−.09	−.32	.07	321.40**	72.62
*Negative affect (work context)*	47	−.03	.23	−.03	.27	−.11	.05	−.35	.29	300.69**	84.70
	OCB‐Os										
Life satisfaction	9	.19	.13	.23	.13	.11	.35	.05	.41	30.10**	73.42
Positive affect	40	.27	.14	.32	.14	.27	.38	.14	.50	139.88**	72.12
*Positive affect (no‐context)*	24	.24	.13	.28	.15	.22	.35	.13	.44	62.97**	63.48
*Positive affect (work context)*	18	.33	.13	.39	.16	.31	.47	.20	.57	63.01**	73.02
Negative affect	34	−.14	.17	−.16	.18	−.24	−.09	−.41	.08	162.46**	79.69
*Negative affect (no‐context)*	22	−.12	.19	−.14	.23	−.25	−.04	−.42	.13	112.29**	81.30
*Negative affect (work context)*	16	−.10	.21	−.12	.25	−.25	.01	−.42	.18	86.62**	82.68
	OCB‐is										
Life satisfaction	12	.26	.20	.30	.20	.16	.44	.02	.57	73.11**	84.96
Positive affect	68	.26	.15	.30	.14	.26	.34	.11	.48	247.39**	72.92
*Positive affect (no‐context)*	43	.28	.16	.31	.19	.26	.37	.10	.53	179.29**	76.57
*Positive affect (work context)*	32	.23	.12	.26	.14	.21	.31	.13	.39	68.26*	54.58
Negative affect	57	−.08	.14	−.09	.13	−.13	−.05	−.26	.08	161.68**	65.36
*Negative affect (no‐context)*	38	−.07	.13	−.08	.15	−.13	−.03	−.23	.08	91.81**	59.70
*Negative affect (work context)*	26	−.07	.17	−.08	.20	−.16	.00	−.30	.15	97.55**	74.37

*Note*: k = Number of independent samples or studies; *r* = Observed correlation coefficient; SD_r_ = Standard deviation of observed correlation coefficients; ρ = Corrected or true score correlation; SDρ = Standard deviation of corrected or true score correlations; 95% CI = 95% Confidence Interval; 80% CR = 80% Credibility Interval; Q = Q statistic; *I* ^*2*^ = I‐squared statistic.

Hypothesis 3 posited that the relationship between OCB‐Is and positive affect/life satisfaction was stronger than their relationship with OCB‐Os, while the converse pattern of relationship was posited for negative affect (i.e., OCB‐Os > OCB‐Is). Using the approach detailed by De Jong et al. (2016), we built 90% confidence intervals around the difference in the effect size estimates. The relationship with OCB‐Is was not significantly different from the relationship with OCB‐Os for either positive affect (Δρ = .02; 90% CI [−.03, .08]) or life satisfaction (Δρ = −.07; 90% CI [−.20, .07]). However, the relationship between negative affect and OCB‐Os was stronger than negative affect's relationship with OCB‐Is (Δρ = −.07; 90% CI [−.14, −.01]), lending partial support for hypothesis 3.

### Longitudinal analyses

Table 3 includes meta‐analytic estimates of the longitudinal effects of SWBs on OCBs. There was some evidence supporting the existence of a longitudinal relationship between OCBs and SWB as positive affect (ρ = .39, *k* = 8, 95% CI [.24, .54]) and negative affect (ρ = −.13, *k* = 15, 95% CI [−.22, −.05]) both significantly predicted future OCBs, however, life satisfaction did not significantly predict future OCBs (ρ = .16, 95% CI [−.13, .45]), which had the least amount of studies to examine this research question (*k* = 3). Similarly, OCBs did not significantly predict future positive affect (ρ = .20, 95% CI [−.28, .68]) or future negative affect (ρ = −.05, 95% CI [−.89, .77]), which was also tested using very few studies (OCBs ➔ future positive affect, *k* = 4; OCBs ➔ future negative affect; *k* = 3). The relationship between OCBs and future life satisfaction could not be explored as there were no studies that examined that relationship. Therefore, our ability to speak to both causal directions was limited by the number of studies available.

**TABLE 3:** Meta‐analytic longitudinal effect sizes between subjective well‐being and organizational citizenship behaviors.

						95% CI		80% CR			
	k	*r*	SD_r_	ρ	SDρ	LL	UL	LL	UL	Q	*I* ^*2*^
Life satisfaction ➔ OCBs	3	.14	.11	.16	.12	−.13	.45	.00	.32	4.21	52.47
Positive affect ➔ OCBs	8	.35	.15	.39	.18	.24	.54	.16	.61	31.38**	77.69
Negative affect ➔ OCBs	15	−.12	.13	−.13	.15	−.22	−.05	−.30	.04	41.29**	66.09
OCBs ➔ positive affect	4	.17	.27	.20	.30	−.28	.68	−.26	.66	21.58**	86.10
OCBs ➔ negative affect	3	−.05	.30	−.06	.34	−.89	.77	−.64	.52	12.86**	84.45

*Note*: k = Number of independent samples or studies; *r* = Observed correlation coefficient; SD_r_ = Standard deviation of observed correlation coefficients; ρ = Corrected or true score correlation; SDρ = Standard deviation of corrected or true score correlations; 95% CI = 95% Confidence Interval; 80% CR = 80% Credibility Interval; Q = Q statistic; *I* ^*2*^ = I‐squared statistic.

### Within‐person analyses

To examine Hypothesis 4, we used the number of responses from each study in our analyses rather than the sample size to estimate the effect size.^2^ As summarized in Table 4, hypothesis 4a was supported as positive affect demonstrated a positive relationship with OCBs (ρ = .18, *k =* 23, 95% CI [.13, .24]), whereas hypothesis 4b was not supported as negative affect was not significantly related to OCBs (ρ = .05, *k* = 21, 95% CI [−.01, .10]). Exploratory analyses demonstrated positive affect was significantly related to OCB‐Is (ρ = .13, *k* = 12, 95% CI [.08, .18]) but not OCB‐Os (ρ = .17, *k* = 4, 95% CI [−.02, .35]) and that negative affect was positively related to OCB‐Is (ρ = .07, *k* = 9, 95% CI [.02, .12]), but not OCB‐Os (ρ = −.01, *k* = 3, 95% CI [−.12, .10]). Importantly, the supplementary results for OCB‐Os contained very few studies (*k* = 3–4).

**TABLE 4:** Meta‐analytic effect sizes between subjective well‐being and organizational citizenship behaviors at the within‐person level.

						95% CI		80% CR			
	k	*r*	SD_r_	ρ	SDρ	LL	UL	LL	UL	Q	*I* ^*2*^
	Organizational citizenship behaviors										
Positive affect	23	.16	.11	.18	.13	.13	.24	.02	.34	163.80**	86.57
*Positive affect (no‐context)*	14	.13	.09	.14	.10	.08	.21	.01	.28	69.12**	81.19
*Positive affect (work context)*	13	.17	.12	.19	.14	.10	.28	.01	.38	105.72**	88.65
Negative affect	21	.04	.11	.05	.12	−.01	.10	−.10	.20	134.14**	85.09
*Negative affect (no‐context)*	13	.02	.11	.03	.11	−.05	.10	−.13	.18	76.84**	84.38
*Negative affect (work context)*	13	.07	.11	.08	.13	.00	.16	−.08	.24	86.75**	86.17
	OCB‐Os										
Positive affect	4	.16	.11	.17	.11	−.02	.35	−.01	.35	24.01**	87.50
Negative affect	3	−.01	.04	−.01	.01	−.12	.10	−.03	.01	2.15	7.11
	OCB‐is										
Positive affect	12	.12	.08	.13	.07	.08	.18	.03	.23	34.16**	67.79
Negative affect	9	.06	.06	.07	.05	.02	.12	.01	.14	15.86*	49.55

*Note*: k = Number of independent samples or studies; *r* = Observed correlation coefficient; SD_r_ = Standard deviation of observed correlation coefficients; ρ = Corrected or true score correlation; SDρ = Standard deviation of corrected or true score correlations; 95% CI = 95% Confidence Interval; 80% CR = 80% Credibility Interval; Q = Q statistic; *I* ^*2*^ = I‐squared statistic.

### Moderator results

**FIGURE 2:**
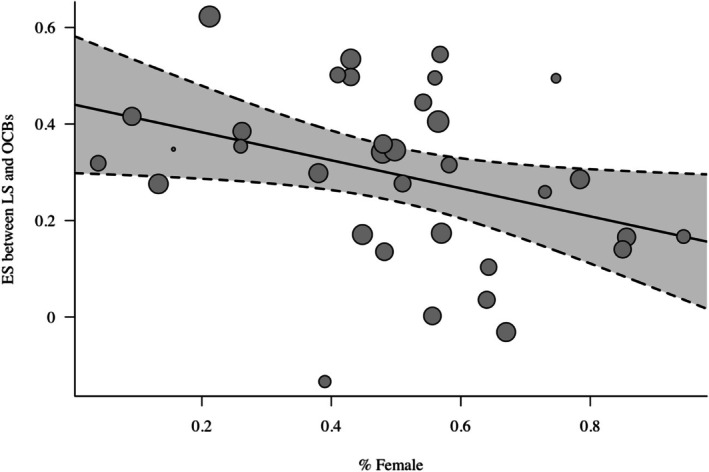
Effect size between life satisfaction and OCBs in relation the proportion of females in the sample.

For the between‐person and within‐person analyses, the Q and *I* ^*2*^ statistics indicated enough heterogeneity in effect size estimates to explore moderator hypotheses. Hypothesis 5 received some support as gender moderated the relationship between life satisfaction and OCBs at the between‐person level (*est* = −.29, *p* < .05), which shows that the relationship between OCBs and life satisfaction decreases as the sample is more represented by females (Figure 2). Hypothesis 6 was not supported, as age did not moderate the relationship between SWB and OCBs at either the between‐ or within‐person levels. Hypothesis 7 was also not supported as general positive affect (between; ρ = .34, within; ρ = .14) and work positive affect (between; ρ = .34, within; ρ = .19) predicted OCBs with similar magnitude (between, Δρ = .00; 90% CI [−.05, .05]; within, Δρ = −.05; 90% CI [−.13, .03]). While there was a significant difference for negative affect at the between‐person level (general negative affect, ρ = −.12, work negative affect ρ = −.03; Δρ = −.09; 90% CI [−.16, −.02]) it was not in the hypothesized direction. Moreover, there was not a significant difference between these same effects at a within‐person level (general negative affect, ρ = .03, work negative affect ρ = .08; Δρ = −.05; 90% CI [−.13, .03]).

## DISCUSSION

The present study makes a substantial contribution to the literature by providing a comprehensive and up‐to‐date account of the relationship between OCBs and SWB, not only expanding on previous work by including life satisfaction, but also comparing different types of OCBs, and offering an initial meta‐analytic account of within‐person effects. By synthesizing findings from a broad range of studies and employing meta‐analytic techniques, this research contributes significantly to our understanding of the interplay between employee well‐being and prosocial behaviors at work. Our findings support the expected direct effects at a high level of abstraction (e.g., the positive association between life satisfaction and OCBs) while also highlighting specific nuances like the differences between OCBs and OCBIs and the within‐person effects. This multifaceted approach provides valuable insights into how different aspects of SWB interact with various types of OCBs at different levels of analysis, emphasizing the complexity and depth of these relationships.

### Moderators

One of the ways that we examined this complexity is through moderators, where we found that the relationship between life satisfaction and OCBs was weaker in studies with a higher proportion of female participants. This finding aligns with the idea that women may receive less of the well‐being benefits from engaging in prosocial behaviors at work, possibly due to differing social expectations and roles. Women might experience more pressure to engage in OCBs regardless of their life satisfaction levels, or they may derive less personal satisfaction from these behaviors due to existing social norms. Future research should explore these gender differences in more detail to understand the underlying mechanisms and how organizational practices can be tailored to support all employees effectively.

While our other moderator hypotheses were not supported, it is important to note that examining these relationships at a high level of abstraction might obscure important nuances. For example, supplemental analysis showed a trend that the relationship between positive affect and OCB‐Os tended to be stronger among employees when the sample was older than 39 ($\rho_{>39}$= .42; $\rho_{<39}$ = .30; Δρ = .12, 90% CI [.02, .22]) whereas this difference was not present for OCB‐Is ($\rho_{>39}$= .30; $\rho_{<39}$ = .32; Δρ = −.01, 90% CI [−.09, .06]). Given that our results are based on the average age within a study and the increasing average age of the workforce (Bureau of Labor Statistics, 2023), understanding how age influences the interplay between affect and OCBs is still likely crucial. Older employees might value organizationally focused citizenship behaviors more highly due to their greater experience and long‐term commitment to the organization, while younger employees might prioritize different forms of engagement. Future research is needed to precisely delineate the presence and strength of these effects, providing insights that can inform age‐inclusive organizational strategies and support the well‐being and productivity of an aging workforce.

Our work found that negative affect had a stronger relationship with OCB‐Os than OCB‐Is. These results support the idea that negative feelings toward specific individuals can spill over and negatively influence behaviors directed at the organization, as suggested by the “target similarity” model proposed by Lavelle et al. (2007). However, we did not find a stronger relationship between positive affect or life satisfaction and OCB‐Is compared to OCB‐Os. Our original logic was that helping individuals would result in greater well‐being benefits due to the greater likelihood of fulfilling psychological needs when helping a colleague as opposed to the organization. Moreover, we posited that the well‐being experienced from OCB‐Is might be higher than that from OCB‐Os, as individuals may have a greater sense of choice when engaging in OCB‐Is. Given that we did not find a difference, it is possible that we did not sufficiently account for how volitional OCB‐Is are as represented by our data. Some have suggested that employees may not engage in OCBs by choice, but as a result of feelings of obligation (Organ, 1990; Thompson et al., 2020). While helping individuals benefit well‐being, it could be that the lack of volition in these actions leads to equivalent relationships for both OCB‐Is and OCB‐Os. Indeed, the sense of choice or volition plays a critical role in the happiness experienced from engaging in behaviors (Lyubomirsky et al., 2005; Weinstein & Ryan, 2010).

### Longitudinal findings

Our investigation also marks the first meta‐analytic examination of the longitudinal relationship between OCBs and positive and negative affect. The available evidence indicates that positive and negative affect are associated with future OCBs, although the small number of studies contributing to these estimates limits the strength of the conclusions that can be drawn. Supplementary analyses did not reveal systematic differences based on the length of the lag between measurement periods; however, given the limited statistical power, these null findings should also be interpreted cautiously. When considered together, the current longitudinal results offer an initial indication that affect may play a role in shaping later citizenship behavior.

Our goal was also to explore whether OCBs predict later well‐being, but here the literature remains especially underdeveloped. Specifically, although we present findings for OCBs predicting future positive and negative affect, we encourage readers to interpret these findings cautiously as they are based on a limited number of studies (three to four studies). The same caution applies to our findings on life satisfaction predicting future OCBs. Despite these limitations, the preliminary results, coupled with findings from other research on prosocial behaviors (Aknin et al., 2019; Hui, 2022; Hui et al., 2020), suggest that this is a fruitful avenue for future research. If true, this work will demonstrate that helping at work not only promotes happiness but also makes happier people more inclined to help others.

### Within‐person findings

These efforts represent the first meta‐analytic examination of within‐person effects, and the results are promising. We uncovered that the dynamic nature of daily fluctuations in affect associated with OCBs may not operate the same way at the within‐person level as they do at the between‐person level. Daily feelings of positive affect are associated with engaging in more OCBs, both generally and specifically for OCB‐Is. Moreover, while there is no significant relationship between negative affect and OCBs or OCB‐Os at a daily level, there is a *positive* relationship between negative affect and OCB‐Is. These findings align with previous meta‐analytic results, which have shown a weaker relationship between OCBs and negative affect when NA is measured as a state (e.g., Geiger et al., 2019; Shockley et al., 2012). However, they also reveal new insights when this relationship is broken down into different types of OCBs.

When considering the relationship between overall OCBs and negative affect, our results align with the Conservation of Resource theory perspective (Hobfoll, 1989), which suggests that individuals experiencing negative affect on a given day may conserve their resources and refrain from voluntary OCBs, focusing instead on essential tasks. This conservation behavior explains why negative affect does not negatively impact OCBs, as employees may still perform OCBs that are perceived as required by their job roles. Yet, this explanation does not fully account for the positive relationship between negative affect and OCB‐Is. To do so, we can leverage social exchange theory (Blau, 2017; Cropanzano & Mitchell, 2005).

Individuals experiencing negative emotions may be more inclined to engage in OCB‐Is in hopes of alleviating their negative feelings, either through the positive emotions that typically accompany such behaviors or through the expectation of immediate reciprocity, a concept often used to explain the motivation for engaging in OCBs (Elstad et al., 2011; Liaquat & Mehmood, 2017). From a COR perspective, this is most likely when individuals perceive helping as a strategic resource investment with a reasonable expectation of return, rather than as an act requiring substantial emotion regulation or resource expenditure. Moreover, the idea of immediate reciprocity could explain why this relationship is not apparent for OCB‐Os, as the reciprocation for engaging in those behaviors may be delayed. This not only provides new insights into the literature but also reinforces our earlier point that the relationship between affect and OCBs can be nuanced.

### Practical implications

Organizations should recognize that employee well‐being plays a critical role in their propensity to engage in OCBs, given our finding that positive affect and life satisfaction are associated with greater OCBs cross‐sectionally and over time. Organizations can benefit from evidence‐based practices and policies designed to facilitate employee happiness, not just at work but in general, which can yield significant payoffs for both employee functioning and organizational outcomes. Examples include work‐life balance programs, flexible scheduling, and access and support to development and growth opportunities. Such practices can enhance overall life satisfaction and help sustain prosocial behavior at work. Initiatives that foster meaning and purpose in work (e.g., Wrzesniewski et al., 1997), encourage job crafting (e.g., Berg et al., 2013), and build a culture of fairness and recognition (e.g., Colquitt et al., 2001) may be particularly effective in reinforcing employees' willingness to go beyond formal job duties.

In addition to these broader organizational strategies, our findings emphasize that these benefits manifest not only at a broad level but also on a daily basis and over time. Managers can use this insight to provide regular check‐ins that focus on emotional support rather than solely on tasks, timely acknowledgment of efforts, opportunities for small “wins” and autonomy throughout the workday, and encouraging employees to take micro‐breaks and engage in short recovery moments (e.g., Sonnentag, 2018). Such practices can help maintain a positive work environment and encourage continuous engagement in OCBs. Overall, at a broader level, practices such as promoting meaningful work, role‐modeling of work‐life balance from the leadership, allowing space and time for growth opportunities, and supporting employee autonomy can bolster overall life satisfaction, further enhancing prosocial behavior over time.

Moreover, awareness of equitable environments for OCB expectations is also essential. Our findings indicate that the relationship between OCBs and life satisfaction is potentially weaker for women, suggesting that engaging in OCBs may be less fulfilling for women compared to men. This could be due to the fact that women may carry more invisible or relational labor in organizations (e.g., Heilman & Chen, 2005), and men are more likely than women to receive organizational rewards for OCB engagement {Citation}. This highlights the need for organizations to manage expectations equitably and ensure that rewards for OCBs are given equally and consistently for all employees. Promoting transparent recognition systems, monitoring informal helping expectations, and regularly reviewing workload and invisible labor distribution can help foster a more inclusive and supportive workplace where all employees feel valued and recognized for their contributions. Taken together, these recommendations highlight that cultivating both enduring well‐being and positive daily experiences can help organizations build cultures where OCBs emerge naturally and sustainably, rather than through pressure or obligation.

## LIMITATIONS

Conducting a meta‐analysis inherently involves several subjective judgment calls (e.g., which studies to include, what measures to combine) that can affect the results in undesirable ways. While we attempted to follow meta‐analytic best practices (Rudolph et al., 2020; Schmidt & Hunter, 2015), some of our decisions (e.g., collapse across different types of OCBs) may have resulted in increased variability in effect size estimates (i.e., larger confidence intervals). Moreover, the limited number of samples available for some effect size calculations (e.g., positive and negative affect and OCB‐Os at the within‐person level) may result in less stable estimates. These estimates should be interpreted with caution due to their reliance on a relatively small sample size.

Another limitation of our study is the inability to thoroughly examine the tenets of “The Dark Side” of OCBs, as we could not clearly differentiate between OCBs that are genuinely voluntary and those performed under organizational pressure or with ulterior motives. This distinction is critical because OCBs driven by external pressures or personal gain may not have the same positive impact on well‐being and could potentially lead to adverse outcomes such as burnout or resentment. Future research should aim to distinguish between these different motivations to provide a clearer understanding of OCBs and their effects on employee well‐being.

Third, although our focus is on examining SWB as a general indicator of an employee's well‐being, there are other job‐relevant indicators that were excluded by this design. OCBs have been shown to be related to burnout, emotional exhaustion, job stress, role overload, job satisfaction, and work–family conflict (e.g., Baranik & Eby, 2016; Bolino & Turnley, 2005; Halbesleben et al., 2009; Koopman et al., 2016; Organ & Ryan, 1995). Although our initial rationale was that these outcomes are more domain‐specific and might not reflect broader well‐being processes, our moderation analyses showed no significant differences in the OCB–well‐being relationship when well‐being was measured in a general frame versus a work‐specific frame. This pattern suggests that domain concerns may be less pronounced than assumed and that job‐focused indicators such as job satisfaction, burnout, or work‐related affect may operate as meaningful proxies for broader well‐being. Future meta‐analytic work incorporating these job‐relevant outcomes could therefore help determine whether they exhibit similar or distinct patterns relative to SWB and clarify how domain specificity shapes the OCB–well‐being association.

## FUTURE DIRECTIONS

### Reciprocal influence

As noted above, more studies are needed to comprehensively examine the longitudinal relationship between SWB and OCBs meta‐analytically, which would facilitate the exploration of the reciprocal influence between these two constructs. While we found some evidence of unidirectional influence, there is reason to think that the reciprocal influence may operate differently depending on the constructs in question (e.g., OCB‐Is vs. OCB‐Os) and the level of analysis (e.g., within‐person vs. between‐person). Precision in examining this relationship is critical as it will facilitate theory development. For example, tenets of both the Conservation of Resources (Hobfoll, 1989; Hobfoll et al., 2018) and Broaden‐and‐Build Theory (Fredrickson, 2001; Fredrickson & Branigan, 2005) discuss the possible presence of reciprocal influences between affect and behavior, but whether these tenets accurately explain the relationship between OCBs and SWB is still in question. Moreover, more research on the longitudinal relationship between OCBs and SWB could facilitate more targeted interventions. By identifying the specific ways in which SWB and OCBs influence each other, organizations can design strategies that simultaneously enhance employee well‐being and promote prosocial behaviors, leading to mutually reinforcing positive outcomes.

### Explorations of more complex models

While our research provided evidence of direct effects, there is a need to develop and test more complex models. For instance, Baranik and Eby (2016) leveraged mood regulation theories and Broaden‐and‐Build Theory to examine how engaging in OCB‐Is can facilitate life satisfaction through the experience of positive emotions. Expanding on this approach, future research should continue to explore how different types of OCBs (e.g., OCB‐Os vs. OCB‐Is) interact with various aspects of SWB through mediating and moderating variables. This expansion could also show how OCBs operate outside of the workplace, such as the research by Smith et al. (2020) that focuses on the implications of where OCBs are performed on work‐family dynamics. These more thoughtful examinations will allow for future meta‐analytic investigations to examine a more complex model, the mechanisms through which OCBs affect SWB, and vice versa.

## CONCLUSION

In conclusion, this study underscores the importance of viewing employees as whole individuals whose general well‐being is integrally tied to the behaviors they engage in at work. The insights provided by this meta‐analysis pave the way for future research and organizational practices that prioritize the well‐being of employees, ultimately fostering a more supportive and productive work environment. The findings presented here not only advance our theoretical understanding but also offer actionable guidance for enhancing both employee well‐being and organizational success.

## CONFLICT OF INTEREST STATEMENT

The authors declare that they have no conflicts of interest related to this work.

## ETHICS STATEMENT

Ethics approval was not required for this study, as it involved a meta‐analysis of previously published research.

## Supporting information


**Table S1.** Description of studies included in the current meta‐analysis.
**Table S2.** Summary of Sensitivity Analyses Results.
**Figure S1a.** Forest Plot for LS ↔ OCBs.
**Figure S1b.** CMA Plot for LS ↔ OCBs.
**Figure S1c.** Funnel Plot for LS ↔ OCBs with PET (dark blue, solid), PEESE (dark green, dashed) effect size (black, solid) lines, and estimated confidence 95% CI of effect size.
**Figure S1d.** Leave‐One‐Out Visualization for LS ↔ OCBs.
**Figure S2a.** Forest Plot for PA ↔ OCBs.
**Figure S2b.** CMA Plot for PA ↔ OCBs.
**Figure S2b.** Funnel Plot for PA ↔ OCBs with PET (dark blue, solid), PEESE (dark green, dashed) effect size (black, solid) lines, and estimated confidence 95% CI of effect size.
**Figure S2d.** Leave‐One‐Out Visualization for PA ↔ OCBs.
Figure S3a. Forest Plot for NA ↔ OCBs.
**Figure S3b.** CMA Plot for NA ↔ OCBs.
**Figure S3c.** Funnel Plot for NA ↔ OCBs with PET (dark blue, solid), PEESE (dark green, dashed) effect size (black, solid) lines, and estimated confidence 95% CI of effect size.
**Figure S3d.** Trim‐and‐Fill Funnel Plot for NA ↔ OCBs.
**Figure S2e.** Leave‐One‐Out Visualization for NA ↔ OCBs.
**Figure S4a.** Forest Plot for LS ➔ Future OCBs.
**Figure S4b.** CMA Plot for LS ➔ Future OCBs.
**Figure S4c.** Funnel Plot for LS ➔ Future OCBs with PET (dark blue, solid), PEESE (dark green, dashed) effect size (black, solid) lines, and estimated confidence 95% CI of effect size.
**Figure S4d.** Leave‐One‐Out Visualization.
**Figure S5a.** Forest Plot for PA ➔ Future OCBs.
**Figure S5b.** CMA Plot for PA ➔ Future OCBs.
**Figure S5b.** Funnel Plot for PA ➔ Future OCBs with PET (dark blue, solid), PEESE (dark green, dashed) effect size (black, solid) lines, and estimated confidence 95% CI of effect size.
**Figure S5d.** Leave‐One‐Out Visualization.
**Figure S6a.** Forest Plot for NA ➔ Future OCBs.
**Figure S6b.** CMA Plot for NA ➔ Future OCBs.
**Figure S6c.** Funnel Plot for NA ➔ Future OCBs with PET (dark blue, solid), PEESE (dark green, dashed) effect size (black, solid) lines, and estimated confidence 95% CI of effect size.
**Figure S6d.** Leave‐One‐Out Visualization.
**Figure S7a.** Forest Plot for OCBs ➔ Future PA.
**Figure S7b.** CMA Plot for OCBs ➔ Future PA.
**Figure S7c.** Funnel Plot for OCBs ➔ Future PA with PET (dark blue, solid), PEESE (dark green, dashed) effect size (black, solid) lines, and estimated confidence 95% CI of effect size.
Figure S7d. Leave‐One‐Out Visualization.
**Figure S8a.** Forest Plot for NA ➔ Future OCBs.
**Figure S8b.** CMA Plot for NA ➔ Future OCBs.
**Figure S8c.** Funnel Plot for NA ➔ Future OCBs with PET (dark blue, solid), PEESE (dark green, dashed) effect size (black, solid) lines, and estimated confidence 95% CI of effect size.
**Figure S8d.** Leave‐One‐Out Visualization.
**Figure S9a.** Forest Plot for PA ↔ OCBs at within‐person level.
**Figure S9b.** CMA Plot for PA ↔ OCBs (Within).
**Figure S9c.** Funnel Plot for PA ↔ OCBs for within‐person analysis with PET (dark blue, solid), PEESE (dark green, dashed) effect size (black, solid) lines, and estimated confidence 95% CI of effect size.
**Figure S9d.** Leave‐One‐Out Visualization for PA ↔ OCBs (Within).
**Figure S10a.** Forest Plot for NA ↔ OCBs at within‐person level.
**Figure S10b.** CMA Plot for NA ↔ OCBs (Within).
**Figure S10c.** Funnel Plot for NA ↔ OCBs for within‐person analysis with PET (dark blue, solid), PEESE (dark green, dashed) effect size (black, solid) lines, and estimated confidence 95% CI of effect size.
**Figure S10d.** Leave‐One‐Out Visualization for NA ↔ OCBs (Within).
**Table S2.** Meta‐Analytic Cross‐Sectional Effect Sizes between Subjective Well‐Being and Organizational Citizenship Behaviors at the Between‐Person Level using Between, Fake Between, and Longitudinal Data.
**Table S3.** Meta‐Analytic Cross‐Sectional Effect Sizes between Subjective Well‐Being and Organizational Citizenship Behaviors at the Between‐Person Level using Between, Fake Between, and Longitudinal Data.

## Data Availability

The data supporting the findings of this study are openly available on the Open Science Framework at https://osf.io/uq7jg/?view_only=617163ec6a7c40ddb83cc0a1202eaa27.

## Footnotes

^1^
For the between‐person analysis, correlations from cross‐sectional, longitudinal, and intensive longitudinal design studies were used. In longitudinal studies, each repeated measure of a construct (e.g., Life Satisfaction – Time 1, Life Satisfaction – Time 2) was treated as a separate measure of that construct (e.g., two measures of life satisfaction) and was collapsed using the aforementioned compositing techniques. In intensive longitudinal studies, aggregated within‐person correlations (i.e., between‐person correlations calculated using within‐person data) were used for the between‐person analysis, which has been shown to be relatively equivalent to ‘true’ between‐person effects (e.g., Ilies & Judge, 2004). A comparison of effects from these types of correlations is provided in the online supplementary material.

^2^
Analysis with the sample size is reported in the online supplementary materials, but the results were relatively equivalent.
