# Supplementary material for: Helping hands, flourishing hearts: A meta‐analytic study of organizational citizenship behaviors and subjective well‐being
Source: Appl Psychol Health Well Being. 2026 May 15;18:e70158. doi: 10.1111/aphw.70158 (PMC13179150; doi:10.1111/aphw.70158)
Supplement: Supplementary file 1 — Table S1. Description of studies included in the current meta‐analysis. Table S2. Summary of Sensitivity Analyses Results. Figure S1a. Forest Plot for LS ↔ OCBs. Figure S1b. CMA Plot for LS ↔ OCBs. Figure S1c. Funnel Plot for LS ↔ OCBs with PET (dark blue, solid), PEESE (dark green, dashed) effect size (black, solid) lines, and estimated confidence 95% CI of effect size. Figure S1d. Leave‐One‐Out Visualization for LS ↔ OCBs. Figure S2a. Forest Plot for PA ↔ OCBs. Figure S2b. CMA Plot for PA ↔ OCBs. Figure S2b. Funnel Plot for PA ↔ OCBs with PET (dark blue, solid), PEESE (dark green, dashed) effect size (black, solid) lines, and estimated confidence 95% CI of effect size. Figure S2d. Leave‐One‐Out Visualization for PA ↔ OCBs. Figure S3a. Forest Plot for NA ↔ OCBs. Figure S3b. CMA Plot for NA ↔ OCBs. Figure S3c. Funnel Plot for NA ↔ OCBs with PET (dark blue, solid), PEESE (dark green, dashed) effect size (black, solid) lines, and estimated confidence 95% CI of effect size. Figure S3d. Trim‐and‐Fill Funnel Plot for NA ↔ OCBs. Figure S2e. Leave‐One‐Out Visualization for NA ↔ OCBs. Figure S4a. Forest Plot for LS ➔ Future OCBs. Figure S4b. CMA Plot for LS ➔ Future OCBs. Figure S4c. Funnel Plot for LS ➔ Future OCBs with PET (dark blue, solid), PEESE (dark green, dashed) effect size (black, solid) lines, and estimated confidence 95% CI of effect size. Figure S4d. Leave‐One‐Out Visualization. Figure S5a. Forest Plot for PA ➔ Future OCBs. Figure S5b. CMA Plot for PA ➔ Future OCBs. Figure S5b. Funnel Plot for PA ➔ Future OCBs with PET (dark blue, solid), PEESE (dark green, dashed) effect size (black, solid) lines, and estimated confidence 95% CI of effect size. Figure S5d. Leave‐One‐Out Visualization. Figure S6a. Forest Plot for NA ➔ Future OCBs. Figure S6b. CMA Plot for NA ➔ Future OCBs. Figure S6c. Funnel Plot for NA ➔ Future OCBs with PET (dark blue, solid), PEESE (dark green, dashed) effect size (black, solid) lines, and estimated confidence 95% CI of effect size. Figure S [file APHW-18-0-s001.docx]

Online Supplementary Material

Table of Contents

[Table of Study Characteristics 2](#_Toc214700968)

[Sensitivity Analyses and Plots 6](#_Toc214700969)

[Hypothesis 1 – OCBs and Life Satisfaction/Positive Affect/Negative Affect 6](#_Toc214700970)

[Life Satisfaction 6](#_Toc214700971)

[Positive Affect 11](#_Toc214700972)

[Negative Affect 16](#_Toc214700973)

[Hypothesis 1 – OCBs and Life Satisfaction/Positive Affect/Negative Affect 18](#_Toc214700974)

[Life Satisfaction 18](#_Toc214700975)

[Negative Affect 21](#_Toc214700976)

[Hypothesis 3 – Longitudinal Analysis 23](#_Toc214700977)

[Life Satisfaction 🡪 Future OCB 23](#_Toc214700978)

[Positive Affect 🡪 Future OCB 24](#_Toc214700979)

[Negative Affect 🡪 Future OCB 25](#_Toc214700980)

[OCB 🡪 Future Positive Affect 26](#_Toc214700981)

[OCB 🡪 Future Negative Affect 27](#_Toc214700982)

[Hypothesis 4 – Within-Person Analysis 28](#_Toc214700983)

[Positive Affect 28](#_Toc214700984)

[Negative Affect 29](#_Toc214700985)

[Comparison of Between-Person Effects using Cross-Sectional, Intensive Longitudinal, and Longitudinal Data 30](#_Toc214700986)

[Comparison Between Sample Size vs. Responses for Within-Person Analysis 31](#_Toc214700987)

# Table of Study Characteristics

This table provides a summary of the study characteristics of the meta-analyzed articles. The proportion of the sample that was female and the average age of the sample were examined as potential moderators (Hypotheses 5 and 6, respectively). The context of affect (work vs. general) is indicated in the SWB column, with affect coded as work given the suffix “-Work”; this was used to examine the moderating impact of affective context (Hypothesis 7). Variables are listed at the smallest level of abstraction coded, but were combined in different ways depending on the analysis. For example, OCB-Helping and OCB-Courtesy were combined into an OCB-I composite for analysis comparing OCB-I vs. OCB-O. Affect such as guilt or attentiveness were analyzed as part of the broader affective category (NA/PA). The full dataset and analysis code can be accessed through our online repository <https://osf.io/uq7jg/?view_only=617163ec6a7c40ddb83cc0a1202eaa27>.

| Table S1. Description of studies included in the current meta-analysis | | | | | | | | |
| --- | --- | --- | --- | --- | --- | --- | --- | --- |
| Citation | Publication Type | Sample *n* | Age *M* | % Female | Country of Data Collection | Study Design | OCB Variables | SWB Variables |
| Adams et al., 2013 | Article | 442 | 47.70 | .43 | USA^1^ | Cross-sectional | OCB | LS |
| Alessandri et al., 2012 | Article | 372 | 39.45 | .57 | Italy | Cross-sectional | OCB-I, OCB-O | LS, PA |
| Astakhova, 2014 | Article | 223 | - | .67 | Russia | Cross-sectional | OCB | LS |
| Avey et al., 2008 | Article | 132 | 30.40 | .48 | USA | Longitudinal | OCB-I | PA |
| Aydin, 2019 | Thesis | 424 | 26.50 | .21 | Turkey | Cross-sectional | OCB | LS |
| Baranik & Ebay, 2016 | Article | 202-301 | 43.29 | .64 | USA | Longitudinal | OCB-I | LS, PA |
| Barclay & Kiefer, 2012 | Article | 136 | 31 | .15 | Canada | Cross-sectional | OCB-H | PA-Work, NA-Work |
| Barnes et al., 2017 | Article | 68-223 | 29.95 | .65 | Multiple^1^ | Longitudinal | OCB | NA |
| Barros & Gómez, 2021 | Article | 282-735 | 35.50 | .43 | Chile | Longitudinal | OCB | LS, PA, NA |
| Bartels et al., 2019 | Article | 533 | 32 | .34 | USA^1^ | Cross-sectional | OCB-I, OCB-O | PA, NA |
| Bateman, 2019 | Dissertation | 185 | 44.50 | .56 | USA | Cross-sectional | OCB | LS |
| Belleville et al., 2019 | Article | 129 | 29.50 | .94 | Canada | Cross-sectional | OCB-CV | LS, PA, NA |
| Belschak & Den Hartog, 2009 | Article | 86 | 46 | .31 | The Netherlands | Cross-sectional | OCB-I, OCB-O | PA-Work, NA-Work |
| Binnewies et al., 2009 | Article | 358 | 40.49 | .67 | Germany^1^ | Longitudinal | OCB-I | NA |
| Binnewies et al., 2009 | Article | 133 | 40.64 | .50 | Germany | ESM | OCB | NA |
| Birkeland & Buch, 2015 | Article | 175 | 44.17 | .51 | Norway | Longitudinal | OCB | LS |
| Bolino et al., 2015 | Article | 260 | 46.04 | .31 | Taiwan | Longitudinal | OCB | NA |
| Bolino et al., 2010 | Article | 245 | 28.50 | .57 | USA | Longitudinal | OCB | NA |
| Bormann et al., 2020 | Article | 136 | 36 | .44 | Germany | ESM | OCB | PA-Work, NA-Work |
| Brady et al., 2017 | Article | 221 | 37.84 | .49 | USA/ Canada | Longitudinal | OCB-I | PA, PA-Work, NA, NA-Work |
| Brant, 2018 | Dissertation | 30-200 | 31.46 | .49 | USA | Cross-sectional | OCB-I, OCB-O | PA, NA |
| Byrne & Hochwarter, 2008 | Article; Sample 1 | 256 | 45.68 | .53 | USA | Cross-sectional | OCB-I | NA |
| Byrne & Hochwarter, 2008 | Article; Sample 2 | 143 | 44.69 | .58 | USA | Cross-sectional | OCB | NA |
| Carlson et al., 2014 | Article | 205 | 41.84 | .56 | USA | Cross-sectional | OCB-I, OCB-O | PA-Work |
| Chancellor et al., 2017 | Article | 32 | 35.31 | .16 | Japan | Cross-sectional | OCB | LS |
| Chauhan, 2017 | Dissertation; Sample 1 | 155 | 35.65 | .54 | USA | Cross-sectional | OCB-I | LS |
| Chauhan, 2017 | Dissertation; Sample 2 | 91 | 46.25 | .56 | USA | ESM | OCB-I | LS |
| Chen et al., 2021 | Article | 201 | - | .36 | China | Cross-sectional | OCB | PA |
| Cheung et al., 2018 | Article | 245 | - | - | Hong Kong/ Macau | Cross-sectional | OCB | NA |
| Chow, 2009 | Article | 121 | 30 | .26 | China | Cross-sectional | OCB-H, OCB-CV, OCB-Con | LS |
| Chuang et al. 2019 | Article | 120 | 37.98 | .81 | Taiwan | ESM | OCB-I | PA-Work, NA-Work |
| Chughtai, 2019 | Article | 187 | 33.30 | .04 | Pakistan | Cross-sectional | OCB | LS |
| Cohen, 1999 | Article | 283 | 33.80 | .85 | Israel | Cross-sectional | OCB | LS |
| Conway et al., 2009 | Article | 80 | 35.76 | .76 | USA^1^ | ESM | OCB-H | PA |
| Cooper et al., 2018 | Article; Sample 1 | 228 | 36 | .76 | USA | Cross-sectional | OCB | PA |
| Cooper et al., 2018 | Article; Sample 2 | 258 | 41.43 | .87 | USA | Cross-sectional | OCB | PA |
| Credé et al., 2009 | Article; Sample 1 | 1147 | - | .24 | USA | Cross-sectional | OCB | PA, NA |
| Credé et al., 2009 | Article; Sample 2 | 375 | - | .30 | USA | Cross-sectional | OCB | PA, NA |
| Credé et al., 2009 | Article; Sample 3 | 295 | - | .57 | Australia | Cross-sectional | OCB | PA, NA |
| Dalal et al., 2012 | Article | 191 | 32.67 | .70 | USA | Cross-sectional | OCB | PA, NA |
| De Cremer et al., 2010 | Article | 344 | 42.71 | .35 | The Netherlands | Cross-sectional | OCB-H | PA |
| Dina & Aharon, 2011 | Article | 133 | 36.35 | .87 | Israel | Cross-sectional | OCB-H, OCB-Court, OCB-Con, OCB-S, OCB-CV | PA |
| Donovan, 1999 | Dissertation | 188 | 42 | .63 | USA | Longitudinal | OCB | High PA-Work. Low PA-Work, High NA-Work, Low NA-Work, Pleasant-Work, Unpleasant-  Work |
| Duarte et al., 2018 | Article | 334 | 37 | .64 | United Kingdom | Cross-sectional | OCB-I, OCB-O | PA-Work, NA-Work |
| Edgar et al., 2017 | Article | 281 | 55 | .78 | New Zealand | Cross-sectional | OCB-I | LS |
| Eisenbeiss & van Knippenberg, 2015 | Article | 135 | 41.04 | .20 | Germany | Cross-sectional | OCB-H | Moral Emotions-Work |
| Eissa & Lester, 2018 | Article | 174 | 33.90 | .48 | USA | Cross-sectional | OCB-H | PA-Work |
| Ellen et al., 2017 | Article | 136 | 48.04 | .34 | USA^1^ | Longitudinal | OCB | PA, NA |
| Fehr et al., 2017 | Article | 155 | 32.80 | .48 | China | ESM | OCB | NA |
| Findley et al., 2000 | Article | 199 | 41 | .85 | USA | Cross-sectional | OCB-Con, OCB-Court, OCB-H | NA |
| Fisher, 2002, 2003^2^ | Article | 121 | 37.96 | .73 | Australia | ESM | OCB | PA, PA-Work, NA, NA-Work, LS |
| Fisher, 2003 | Article | 121 | - | .73 | Australia | Cross-sectional | OCB | LS, PA, NA |
| Fletcher & French, 2022 | Unpublished Data | 312 | 34.89 | .40 | USA | Cross-sectional | OCB-I, OCB-O | PA, NA |
| Fluegge, 2008 | Dissertation | 205 | 22 | .49 | USA | Cross-sectional | OCB | PA-Work |
| Fox et al., 2012 | Article; Sample 1 | 136-169 | - | .76 | USA | Cross-sectional | OCB | PA-Work, NA-Work |
| Fox et al., 2012 | Article; Sample 2 | 515 | - | .81 | USA | Cross-sectional | OCB | PA-Work, NA-Work |
| George, 1991 | Article | 169 | - | .84 | USA | Cross-sectional | OCB-H, OCB- Customer | PA-Work |
| Gerpott et al., 2022 | Article | 91 | 36.70 | .77 | UK | ESM | OCB-I | NA-Work |
| Gilmore et al., 2013 | Article | 212 | 30.50 | .74 | China | Cross-sectional | OCB | PA |
| Giluk, 2010 | Dissertation | 24-59 | - | - | USA | Longitudinal | OCB-I | PA, NA |
| Glomb et al., 2011 | Article | 68 | 42 | .54 | USA | ESM | OCB-H, OCB-Court | PA, NA, PA-Work, NA-Work |
| Gooty, 2007 | Dissertation | 29 | - | - | USA | DRM | OCB-I, OCB-O | PA, NA, Anger-Work, Guilt-Work, Joy-Work, Pride-Work |
| Goswami et al., 2016 | Article | 253 | 27.14 | .35 | India | Cross-sectional | OCB | PA-Work |
| Gotlib et al., 2011 | Dissertation | 185 | 35 | .63 | USA | Cross-sectional | OCB-I, OCB-O | PA, NA |
| Greenidge & Coyne, 2014 | Article | 202 | 35 | .50 | Barbados | Cross-sectional | OCB-O, OCB-H, OCB-Court | PA-Work, NA-Work |
| Greguras & Diefendorff, 2010 | Article | 154 | 35.91 | .58 | Singapore | Cross-sectional | OCB | LS |
| Haar & Brougham, 2016 | Article; Sample 1 | 211 | 38.80 | .55 | New Zealand | Cross-sectional | OCB-O | PA, NA |
| Haar & Brougham, 2016 | Article; Sample 2 | 291 | 39.10 | .55 | New Zealand | Cross-sectional | OCB-O | PA, NA |
| Halbesleben et al., 2009 | Article; Sample 1 | 80 | 39.23 | .02 | USA | Longitudinal | OCB-I | NA |
| Halbesleben et al., 2009 | Article; Sample 2 | 513 | 37.98 | .60 | USA^1^ | Longitudinal | OCB-I | NA |
| Halbesleben et al., 2009 | Article; Sample 3 | 251 | 31.90 | .88 | USA^1^ | Cross-sectional | OCB-Customer | NA |
| Hall & Ferris, 2010 | Article | 84-215 | 40.14 | .54 | USA | Cross-sectional | OCB | PA, NA |
| Hill et al., 2021 | Article | 101 | 26.61 | .57 | USA | ESM | OCB | PA-Work, NA-Work |
| Holtom et al., 2012 | Article | 279 | 42.60 | .47 | USA | Longitudinal | OCB | NA |
| Hoon & Ansari, 2005 | Conference Paper | 140 | 31.68 | .56 | Malaysia | Cross-sectional | OCB-H, OCB-Con, OCB-Court, OCB-CV, OCB-S | PA, NA |
| Hoption et al., 2012 | Article | 316 | 20 | .48 | USA/ Canada^1^ | Cross-sectional | OCB | PA-Work |
| Hui et al., 1999 | Article | 347 | 24 | .52 | China/ Hong Kong | Cross-sectional | OCB | NA |
| Hurst, 2010 | Dissertation | 111 | 36 | .69 | USA | ESM | OCB | PA |
| Ilies et al., 2006 | Article | 62 | 36 | .72 | USA^1^ | ESM | OCB | PA-Work |
| Im et al., 2018 | Article | 312 | 34.048 | .47 | South Korea | Cross-sectional | OCB-I | PA |
| Imer et al., 2014 | Article | 360 | 33 | .58 | Turkey | Cross-sectional | OCB-H, OCB-CV, OCB-S | PA, NA |
| Ismail et al., 2018 | Article | 463 | 33 | .70 | Malaysia | Cross-sectional | OCB-H | NA |
| Jang, 2018 | Dissertation; Sample 1 | 812 | 36.84 | .55 | USA | Cross-sectional | OCB-I | PA |
| Jang, 2018 | Dissertation; Sample 2 | 625 | 47.93 | .53 | USA | Longitudinal | OCB-I | PA |
| Janssen et al., 2010 | Article | 241 | 24.10 | .81 | China/ Hong Kong | Cross-sectional | OCB-I, OCB-O | PA-Work, NA-Work |
| Jensen & Raver, 2012 | Article | 212 | 38 | .66 | USA | Cross-sectional | OCB-O | PA, NA |
| R. E. Johnson et al., 2010 | Article | 120 | 35.70 | .41 | USA | Cross-sectional | OCB-I, OCB-O | PA, NA |
| S. K. Johnson, 2008 | Article | 126 | - | .81 | USA^1^ | Cross-sectional | OCB | PA, NA, PA-Work, NA-Work |
| A. M. Jones, 2009 | Dissertation | 116 | 43.34 | .59 | USA^1^ | Cross-sectional | OCB-I | PA-Work, NA-Work |
| M. D. Jones, 2002 | Dissertation | 86 | 28.20 | .59 | USA | Cross-sectional | OCB | LS |
| Kemery et al., 1996 | Article | 65 | 37.20 | .59 | USA | Cross-sectional | OCB-H | PA, NA |
| Khan, 2020 | Article | 213 | 29.50 | .32 | Pakistan | Longitudinal | OCB-I | NA, Anger-Work |
| Kickul & Lester, 2001 | Article | 183 | 31.73 | .46 | USA | Cross-sectional | OCB-H | NA-Work |
| M. Kim & Beehr, 2018 | Article | 347 | 35.39 | .45 | USA | Longitudinal | OCB | LS, NA |
| S. Y. Kim et al., 2013 | Article | 261 | 31.03 | .42 | South Korea | Cross-sectional | OCB-I | PA |
| Kluemper et al., 2009 | Article | 106 | 27 | .43 | USA | Longitudinal | OCB | PA, NA |
| Koopman et al., 2016 | Article | 82 | 43.40 | .83 | USA | ESM | OCB-I | PA, NA |
| Koopman et al., 2019a | Article; Sample 1 | 111 | 37.70 | .76 | USA | ESM | OCB-I | PA, NA |
| Koopman et al., 2019a | Article; Sample 2 | 164 | 35.30 | .79 | USA | Cross-sectional | OCB-I | NA |
| Koopman et al., 2019b | Article | 541 | 40.90 | .60 | USA | Cross-sectional | OCB-I, OCB-O | PA-Work |
| Kwan, 2018 | Dissertation | 585 | 40.50 | .50 | Hong Kong | Cross-sectional | OCB-I, OCB-O | LS |
| Lam et al., 2013 | Article | 245 | 24 | .81 | China | Cross-sectional | OCB | PA-Work, NA-Work |
| Lambert, 2010 | Article | 160 | 33 | .41 | USA | Cross-sectional | OCB | LS |
| Lanaj & Jennings, 2020 | Article | 43 | 38.30 | .21 | USA | ESM | OCB-I | PA, NA, PA-Work, NA-Work |
| Lanaj et al., 2016 | Article | 68 | 33.70 | .32 | USA | ESM | OCB-H | PA |
| Lavy & Littman-Ovadia, 2017 | Article | 1095 | 41.30 | .81 | Multiple | Cross-sectional | OCB | PA-Work |
| H.-J. Lee, 1999 | Dissertation | 224 | 33.40 | .91 | UK | Cross-sectional | OCB-H | High PA-Work,  Low PA-Work, High NA-Work,  Low NA-Work |
| K. Lee, 2000 | Dissertation | 146-215 | 45.43 | .95 | Canada | Cross-sectional | OCB-I, OCB-O | Self-assurance-Work, Attentiveness-  Work, Joviality-Work, Fear-Work, Hostility-Work, Guilt-Work, Sadness-Work |
| Levesque et al., 2004 | Article | 146 | - | .39 | Gabon | Cross-sectional | OCB-H, OCB-Con | LS |
| C. W. Lin et al., 2011 | Article | 357 | 35 | .53 | Taiwan | Cross-sectional | OCB | PA, NA |
| K. J. Lin et al., 2017 | Article; Sample 1 | 86 | 43.30 | .76 | USA | ESM | OCB-I | PA |
| K. J. Lin et al., 2017 | Article; Sample 2 | 95 | 30.10 | .50 | China | ESM | OCB-I | PA, PA-Work |
| Little et al., 2011 | Article | 168 | 29.20 | .48 | USA | Cross-sectional | OCB-I, OCB-O | PA |
| Lloyd et al., 2015 | Article | 328 | 34.40 | .59 | Germany | Cross-sectional | OCB-O | PA-Work; NA-Work |
| Loi et al., 2020 | Article | 136 | 32 | .49 | USA | Longitudinal | OCB | NA |
| Luan et al., 2022 | Article | 209 | 26.21 | .86 | China | Longitudinal | OCB-customer | LS |
| Luksyte et al., 2020 | Article; Sample 1 | 190 | 41.16 | .64 | Multiple | Longitudinal | OCB | LS |
| Luksyte et al., 2020 | Article; Sample 2 | 224 | 37.95 | .48 | USA | Cross-sectional | OCB | LS |
| Mai et al., 2016 | Article; Sample 1 | 60 | 43.18 | .85 | USA | Longitudinal | OCB | NA-Work |
| Mai et al., 2016 | Article; Sample 2 | 130 | 31.60 | .79 | China | Longitudinal | OCB | NA-Work |
| Mazumdar, 2018 | Dissertation | 194 | 66.42 | .48 | Canada | Cross-sectional | OCB | LS |
| McAllister et al., 2016 | Article; Sample 1 | 133 | 44.82 | .51 | USA | Cross-sectional | OCB | PA, NA |
| McAllister et al., 2016 | Article; Sample 2 | 154 | 41.73 | .44 | USA^1^ | Cross-sectional | OCB | PA, NA |
| McAllister et al., 2016 | Article; Sample 3 | 171 | 32.14 | .50 | USA | Cross-sectional | OCB | PA, NA |
| Meyer et al., 2012 | Article | 180-403 | 42.30 | .85 | Canada^1^ | Cross-sectional | OCB-I, OCB-O | PA-Work, NA-Work |
| Meynhardt et al., 2020 | Article | 1045 | 43.259 | .48 | Switzerland | Cross-sectional | OCB-CV | LS |
| Michel, 2016 | Article | 291 | 39.20 | .54 | USA | Cross-sectional | OCB-H, OCB-S | PA-Work |
| Midili, 1996 | Dissertation | 147 | 31 | .28 | USA | Cross-sectional | OCB-H, OCB-S, OCB-Con, OCB-CV, OCB-Court | PA |
| Miles et al., 2002 | Article | 203 | 25 | .68 | USA | Cross-sectional | OCB | PA-Work, NA-Work |
| Mitchell et al., 2019 | Article | 108 | 39.80 | .66 | USA | ESM | OCB | PA-Work, NA-Work |
| Molitor, 1998 | Dissertation | 313 | 41.50 | .81 | USA | Cross-sectional | OCB | PA, NA |
| Moorman, 2016 | Article | 225 | - | - | USA | Cross-sectional | OCB-H, OCB-Court, OCB-S, OCB-Con  OCB-CV | PA, NA |
| Mostafa, 2017 | Article | 362 | 41 | .35 | Wales | Longitudinal | OCB | PA |
| Mugayar-  Baldocchi, 2021 | Dissertation | 188 | 37.10 | .65 | USA | Cross-sectional | OCB-I, OCB-O | NA |
| Mühlemann et al., 2022 | Article | 225 | 43.08 | .26 | Multiple European | Cross-sectional | OCB-O | LS |
| Munson, 2000 | Dissertation; Sample 1 | 453 | 42 | .71 | USA | Cross-sectional | OCB | PA, NA, PA-Work, NA-Work |
| Munson, 2000 | Dissertation; Sample 2 | 364 | 32 | .76 | USA | Cross-sectional | OCB | PA, NA, PA-Work, NA-Work |
| Munyon et al., 2009 | Article; Sample 1 | 165 | 38.90 | .63 | USA | Cross-sectional | OCB-H | PA, NA |
| Munyon et al., 2009 | Article; Sample 2 | 219 | 39.23 | .69 | USA | Cross-sectional | OCB-H | PA, NA |
| Munyon et al., 2009 | Article; Sample 3 | 642 | 35.66 | .53 | USA | Cross-sectional | OCB-H | PA, NA |
| Muric et al., 2022 | Article | 108 | 39.45 | .64 | Spain | Longitudinal | OCB-I | PA-Work |
| Neubert et al., 2008 | Article | 250 | 40.44 | .32 | USA | Longitudinal | OCB | PA, NA |
| Ng & Feldman, 2009 | Article | 162 | 34 | .48 | Hong Kong | Cross-sectional | OCB | PA, NA |
| Niklas, 2008 | Dissertation | 116 | 43.92 | .42 | Germany | Longitudinal | OCB | PA, NA |
| Organ & Konovsky, 1989 | Article | 369 | 35.60 | .80 | USA | Cross-sectional | OCB-H, OCB-Con | PA-Work, NA-Work |
| Patel, 2019 | Dissertation | 64 | 42.10 | .80 | USA | ESM | OCB-I | PA-Work |
| Paul et al., 2019 | Article | 345 | 37 | .13 | India | Cross-sectional | OCB | LS |
| Paul & Garg, 2013 | Article | 240 | 38 | .09 | India | Cross-sectional | OCB | LS, PA, NA |
| Penezić et al., 2013 | Article | 172 | 36.25 | - | Croatia | Cross-sectional | OCB-H, OCB-Con, OCB-Court, OCB-CV, OCB-S | LS |
| Raver, 2004 | Dissertation | 642 | 35.93 | .50 | USA | Cross-sectional | OCB-I, OCB-O | NA-Work |
| Raza et al., 2021 | Article | 258 | 27.82 | .66 | China | Cross-sectional | OCB | PA, NA |
| Richards & Schat, 2011 | Article | 146 | 36.96 | .50 | - | Cross-sectional | OCB-I, OCB-O | PA, NA |
| Rioux & Penner, 2001 | Article | 130 | 45 | .39 | USA | Cross-sectional | OCB-H, OCB-Con, OCB-Court, OCB-CV, OCB-S | PA-Work |
| Robert & Vandenberghe, 2018 | Article | 232 | 38.93 | .64 | Canada/ France | Cross-sectional | OCB-I | PA, NA |
| Rodell & Judge, 2009 | Article | 100 | 32.50 | .78 | USA | ESM | OCB | Attentiveness-  Work, Anxiety-Work, Anger-Work |
| Roney & Soicher, 2022 | Article | 295 | 34 | .38 | USA | Cross-sectional | OCB-Con, OCB-H | LS |
| Rosen et al., 2020 | Article | 84 | 22 | .72 | USA | ESM | OCB | Attentiveness, Anxiety |
| Scott et al., 2018 | Article | 89 | 38.30 | .51 | USA | Cross-sectional | OCB-O | PA |
| Sheridan & Ambrose, 2022 | Article | 79 | 41.40 | .75 | USA | ESM | OCB-H | LS |
| Shih & Chen, 2015 | Article | 485 | 36.60 | .50 | Taiwan | Cross-sectional | OCB | PA |
| Shin & Hur, 2019 | Article | 50 | 29.58 | .82 | South Korea | ESM | OCB | PA-Work, NA-Work |
| Shin et al., 2019 | Article | 152 | 34.50 | .18 | South Korea | Cross-sectional | OCB-O | PA, NA |
| Somech & Ron, 2016 | Article | 104 | 38.50 | .95 | Israel | Cross-sectional | OCB-H, OCB-Con, OCB-Court, OCB-CV, OCB-S | PA, NA |
| Sosik et al., 2019 | Conference Paper | 218 | - | - | USA | Cross-sectional | OCB-I | PA-Work, NA-Work |
| Spence et al., 2014 | Article | 104 | 38.68 | .44 | Canada^1^ | ESM | OCB-I, OCB-O | PA-Work |
| Spence et al., 2011 | Article | 99 | 32.12 | .65 | Canada | ESM | OCB | PA-Work, NA-Work |
| Spitzmuller et al., 2020 | Article | 33 | 32 | .94 | Singapore | ESM | OCB-I | PA, PA-Work, NA, NA-Work |
| Tang et al., 2020 | Article; Sample 1 | 91 | 28.73 | .58 | China | ESM | OCB-O, OCB-  Customer | PA, NA, Guilt, Guilt-Work |
| Tang et al., 2020 | Article; Sample 2 | 78 | 33.70 | .53 | Hong Kong | ESM | OCB-O, OCB-  Customer | PA, NA, Guilt, Guilt-Work |
| Tenhiälä & Lount, 2013 | Article | 494 | - | .46 | Finland | Cross-sectional | OCB-H | PA, NA |
| Tepper et al., 2018 | Article | 93 | 43.30 | .79 | USA | ESM | OCB-I, OCB-O | PA, NA, PA-Work, NA-Work |
| Thomas, 2011 | Dissertation | 176 | 29.42 | .54 | USA | Cross-sectional | OCB | PA; NA |
| Tobares, 2009 | Thesis | 298 | 20.07 | .43 | USA^1^ | Cross-sectional | OCB-I, OCB-O | PA-Work, NA-Work |
| Tsai et al., 2007 | Article | 306 | 37.80 | .73 | Taiwan | Cross-sectional | OCB-I | PA |
| Uy et al., 2017 | Article | 102 | 28 | .59 | Singapore | ESM | OCB-I | PA, NA |
| Van Woerkom & Meyers, 2015 | Article | 405 | 41.90 | .50 | The Netherlands/ Belgium | Cross-sectional | OCB | PA-Work |
| Wayne et al., 2021 | Article | 596 | 54 | .57 | USA | Cross-sectional | OCB | LS |
| L. J. Williams, 1988 | Dissertation | 85-286 | 30 | .35 | USA | Cross-sectional | OCB-I, OCB-O | PA-Work, NA-Work |
| L. J. Williams & Anderson, 1991 | Article | 127 | 30 | .32 | USA | Cross-sectional | OCB-I, OCB-O | Negative Activation-  Work, Positive Arousal-Work |
| S. Williams & Shiaw, 1999 | Article | 139 | 28 | .58 | Singapore | Cross-sectional | OCB | PA, NA |
| Wu et al., 2014 | Article | 131 | 36.20 | .50 | Taiwan | Cross-sectional | OCB-I, OCB-O | PA |
| Yam et al., 2017 | Article; Sample 1 | 345 | 32.05 | .45 | China | Cross-sectional | OCB | NA |
| Yam et al., 2017 | Article; Sample 2 | 180 | 38.21 | .33 | USA^1^ | Cross-sectional | OCB | NA |
| Yang et al., 2016 | Article | 71 | 27.77 | .49 | China | ESM | OCB | PA, NA |
| Yue et al., 2016 | Article | 106 | - | .62 | China | Longitudinal | OCB-I | PA, NA |
| Yue et al., 2017 | Article; Sample 1 | 70 | 35.70 | - | China | ESM | OCB-H | NA |
| Yue et al., 2017 | Article; Sample 2 | 54 | 36.17 | - | Chine | ESM | OCB-Customer | NA |
| Zellars et al., 2002 | Article | 373 | 26 | .08 | USA | Cross-sectional | OCB | PA, NA |
| Zhou et al., 2021 | Article | 79 | 31.64 | .49 | China | ESM | OCB-H | PA-Work |
| Ziegler et al., 2012 | Article | 92 | 50.50 | .49 | Germany | Longitudinal | OCB | PA, NA, PA-Work, NA-Work |
| Notes. ^1^Location of data collection inferred by location of primary author institution. ^2^Fisher, 2002 and Fisher, 2003 used the same sample but provided some unique correlations of interest to our study; therefore, we coded these as a single article to avoid duplicating the sample. ESM = Experience Sampling Method; OCB-H = Helping/Altruism; OCB-S = Sportsmanship; OCB-Con = Conscientiousness; OCB-CV = Civic Virtue; OCB-Court = Courtesy; OCB-I = Interpersonal; OCB-O = Organizational; PA = Positive Affect in General; PA-Work = Positive Affect at Work; NA = Negative Affect in General; NA-Work = Negative Affect at Work; LS = Life Satisfaction | | | | | | | | |

# Sensitivity Analyses and Plots

To evaluate the potential influence of publication bias. *p*-hacking, and study effects, we conducted a series of sensitivity analyses (e.g., Banks et al., 2012; Morris, 2023) for the three focal associations examined in Hypotheses 1, 3, and 4. That is, the relationships between overall OCBs and each component of subjective well-being (life satisfaction, positive affect, and negative affect). Specifically, we conducted a cumulative meta-analysis (Borenstein, Hedges, Higgins, & Rothstein, 2005), PET–PEESE (Stanley & Doucouliagos, 2014), Trim-and-Fill (Duval & Tweedie, 2000), and a one-study-removed analysis (Borenstein et al., 2009). The cumulative meta-analysis (CMA) involved ordering effect sizes by sample size and re-estimating the cumulative effect one study at a time. When studies are sorted by sample size, upward “drift” in the cumulative effect indicates that small-sample, small-magnitude effects may be missing, consistent with potential publication bias. PET–PEESE is a regression-based approach used to detect funnel-plot asymmetry. Significant PET or PEESE estimates indicate a systematic relationship between effect sizes and their precision, suggesting the possibility that observed effects may be inflated due to publication bias or related research practices. Trim-and-Fill provides an adjusted estimate of the effect size by imputing the number and location of potentially missing studies, offering an approximation of what the pooled effect would be under a more symmetric distribution. Finally, the one-study-removed analysis evaluates the stability of the findings by assessing whether any single study disproportionately influences the overall effect.

A summary of these sensitivity analyses is presented in Table S2. Overall, the cross-sectional association appears to show some indications of publication bias, specifically with negative affect. The cumulative meta-analysis revealed a notable discrepancy between the largest-sample study (Credé et al., 2009, Study 1; ρ = .01, n = 1,147) and the final cumulative estimate (ρ = –.11). PET–PEESE analyses also indicated significant funnel-plot asymmetry, and the Trim-and-Fill results suggested that the adjusted effect could be somewhat stronger (ρ = –.15) than the estimate reported in the main analyses. Although these diagnostics are informative, they do not substantively alter the interpretation presented in the primary manuscript. For the within-person relationship, several sensitivity tests also signaled potential bias for the negative affect’s association with OCBs. The largest-sample study (Fehr et al., 2017; ρ = –.20, n = 1,532) visibly influenced the cumulative trend, as evidenced by drift in the CMA plot. However, neither PET nor PEESE was statistically significant, and the influence of this study on the pooled effect was minimal. A supplementary Trim-and-Fill analysis indicated that imposing funnel-plot symmetry would increase the effect size by only .02, and removing the Fehr et al. study increased the estimate by just .01. Together, these results suggest that although minor indications of bias are present, they are weak and would not meaningfully change the conclusions drawn from the main analyses.

| Table S2. Summary of Sensitivity Analyses Results. | | | | | | |
| --- | --- | --- | --- | --- | --- | --- |
|  | CMA | | PET-PEESE | | Trim-and-Fill | |
|  | 𝛥𝝆^1^ | Drift? | PET | PEESE | Original 𝝆 | Filled 𝝆 |
| Hypothesis 1 |  |  |  |  |  |  |
| LS ↔ OCB | .00 | No | *ns* | *ns* | N/A | N/A |
| PA ↔ OCB | .00 | No | *ns* | *ns* | N/A | N/A |
| NA ↔ OCB | .12 | No | *z* = 3.63, *p* < .001 | *z* = 3.66, *p* < .001 | -.11 | -.15 |
|  |  |  |  |  |  |  |
| Hypothesis 3 |  |  |  |  |  |  |
| LS 🡪 OCB | .01 | No | *ns* | *ns* | N/A | N/A |
| PA 🡪 OCB | .05 | No | *ns* | *ns* | N/A | N/A |
| NA 🡪 OCB | .02 | No | *ns* | *ns* | N/A | N/A |
| OCB 🡪 PA | .11 | No | *ns* | *ns* | N/A | N/A |
| OCB 🡪 NA | .35 | No | *ns* | *ns* | N/A | N/A |
|  |  |  |  |  |  |  |
| Hypothesis 4 |  |  |  |  |  |  |
| PA ↔ OCB | .04 | No | *ns* | *ns* | N/A | N/A |
| NA ↔ OCB | .25 | Yes | *ns* | *ns* | .05 | .07 |
| *Note.* ^1^𝛥𝝆 is the differences between the largest *n* study and the overall effect size | | | | | | |

## Hypothesis 1 – OCBs and Life Satisfaction/Positive Affect/Negative Affect

### Life Satisfaction

A forest plot of the effects is seen in Figure S1a. For the CMA, there was no drift observed as the sample with the largest sample (Meynhardt et al., 2020, *ρ* = .34, *n* = 1,045) did not drift meaningfully (Figure S1b) or deviate from the final effect size (*ρ* = .34). The PET-PEESE analysis indicated that neither the PET (z = -.47, p > .05) or PEESE (z = -.20, p > .05) indicated significant funnel plot asymmetry, suggesting no publication bias or small-study effects in the meta-analysis of Life Satisfaction. Moreover, the contour-enhanced funnel plot (Figure S1c) did not indicate clustering within that significant p-value range, suggesting a lack of significant p-hacking in the results. Lastly, an examination of the one-sample removed figure (Figure S1d) shows that no one study significantly influenced the results of these effect sizes.

| 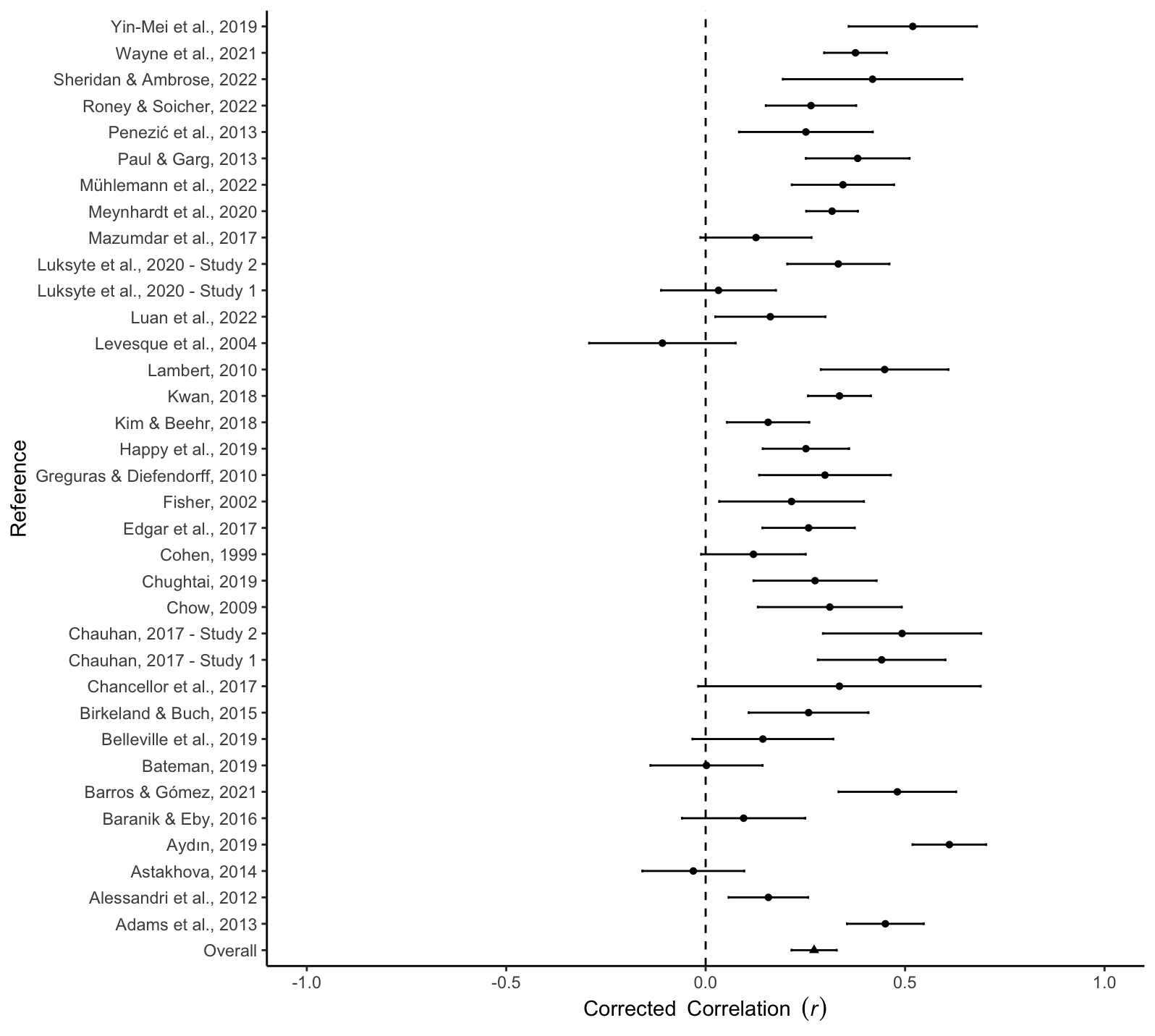 |
| --- |
| Figure S1a. Forest Plot for LS ↔ OCBs |

| 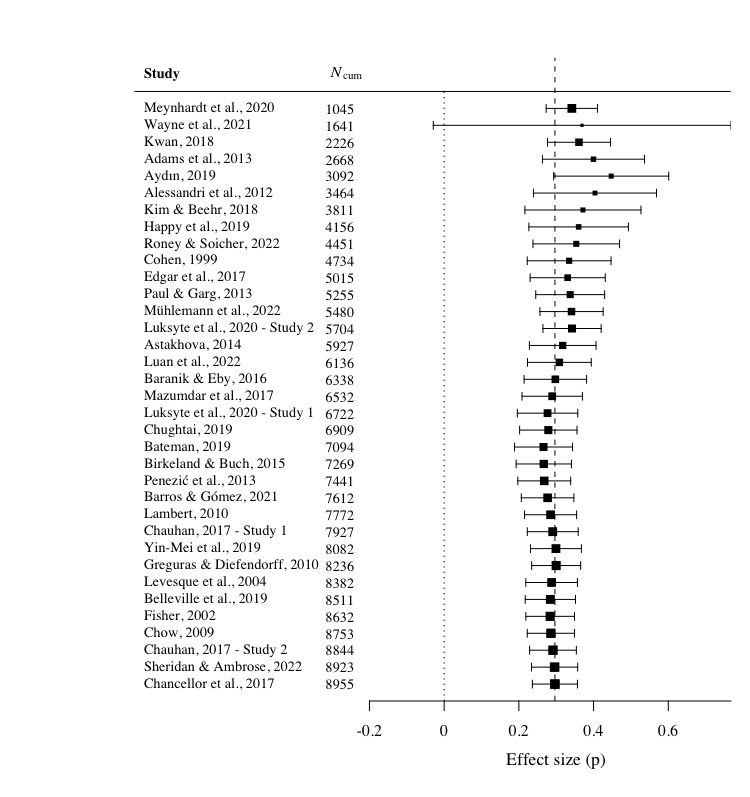 |
| --- |
| Figure S1b. CMA Plot for LS ↔ OCBs |

| 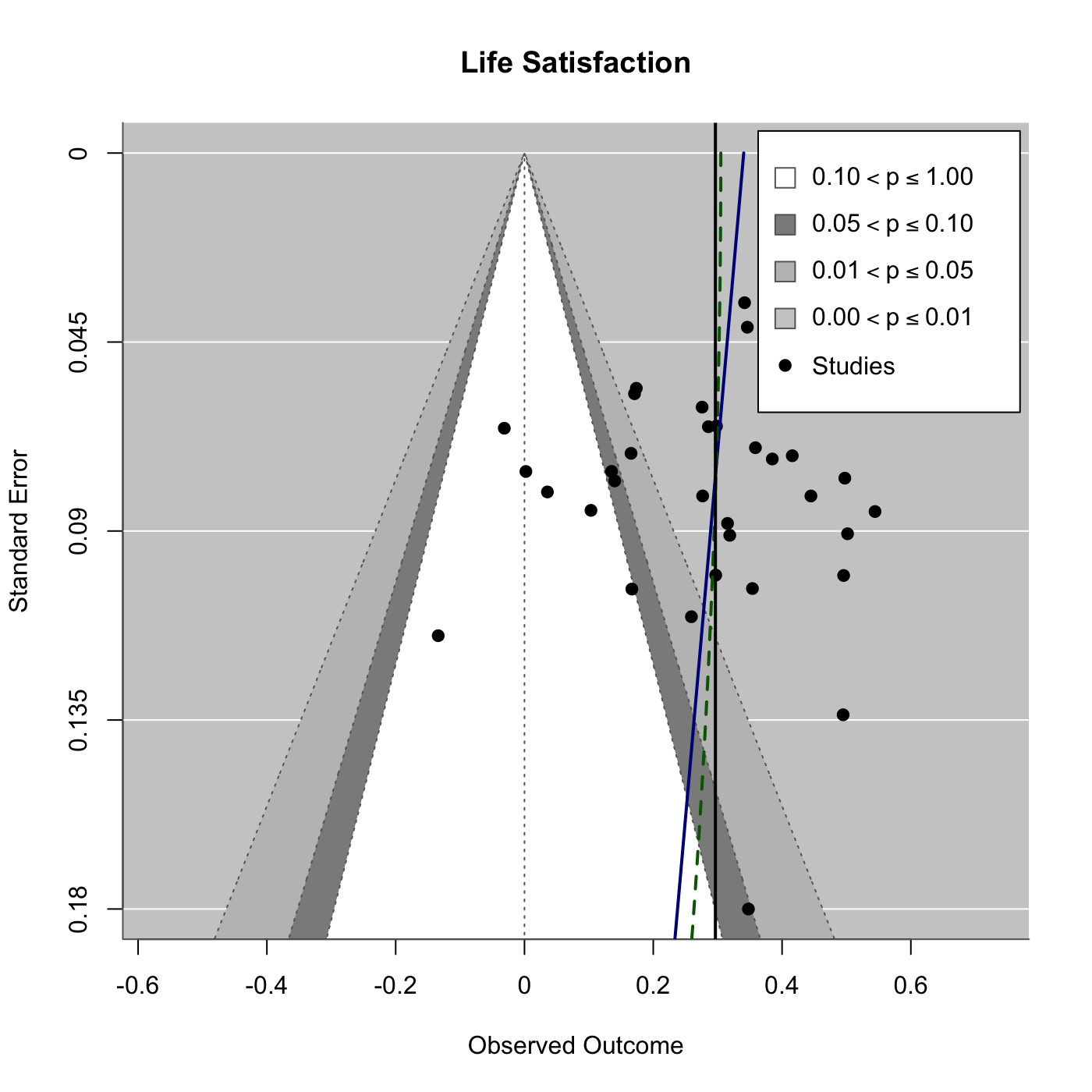 |
| --- |
| Figure S1c. Funnel Plot for LS ↔ OCBs with PET (dark blue, solid), PEESE (dark green, dashed) effect size (black, solid) lines, and estimated confidence 95% CI of effect size |

| 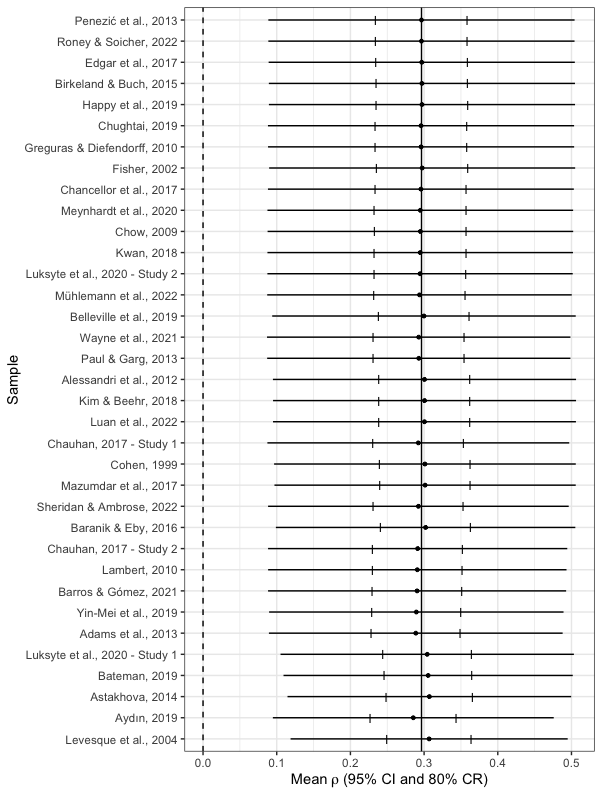 |
| --- |
| Figure S1d. Leave-One-Out Visualization for LS ↔ OCBs |

### Positive Affect

The forest plot for the PA ↔ OCB effects is seen in Figure S2a. For the CMA, there was also no drift observed (Figure S2b). Moreover, the largest sample (Credé et al., 2009 - Study 1, *ρ* = .34, *n* = 1,147) did not deviate at all from the final effect size (*ρ* = .34). The PET-PEESE analysis for Positive Affect was conducted to assess funnel plot asymmetry. Neither the PET (*z* = -1.78, *p* > .05) nor PEESE (*z* = -1.66, *p* > .05) indicated significant funnel plot asymmetry, suggesting no publication bias or small-study effects in the meta-analysis of Positive Affect. Moreover, the contour-enhanced funnel plot (Figure S2c) did not indicate that studies were clustering within that significant p-value range, which indicates that lack of significant p-hacking in the results. Lastly, an examination of the one-sample removed figure (Figure S2d) shows that no one study significantly influenced the results of these effect sizes.

| 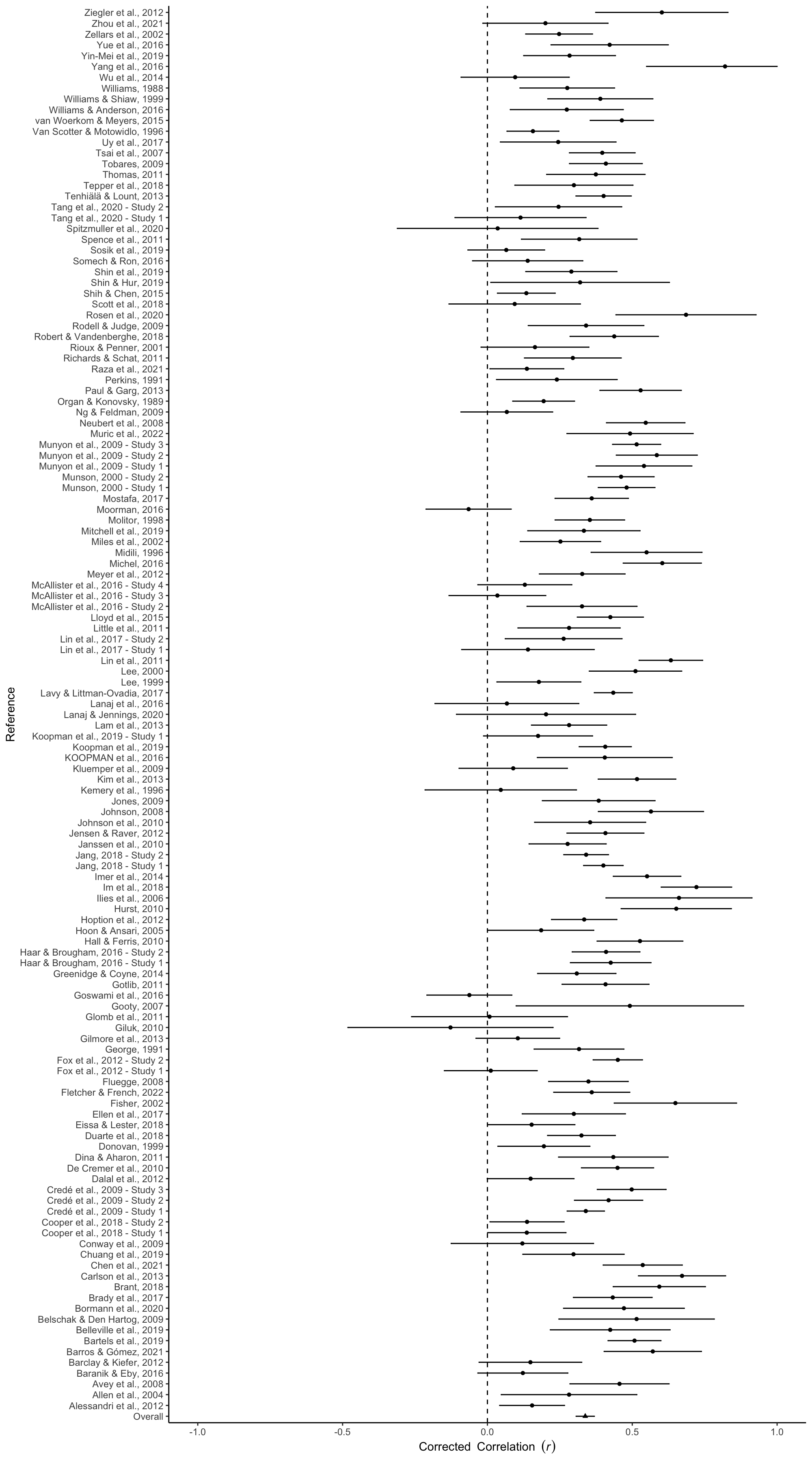 |
| --- |
| Figure S2a. Forest Plot for PA ↔ OCBs |

| 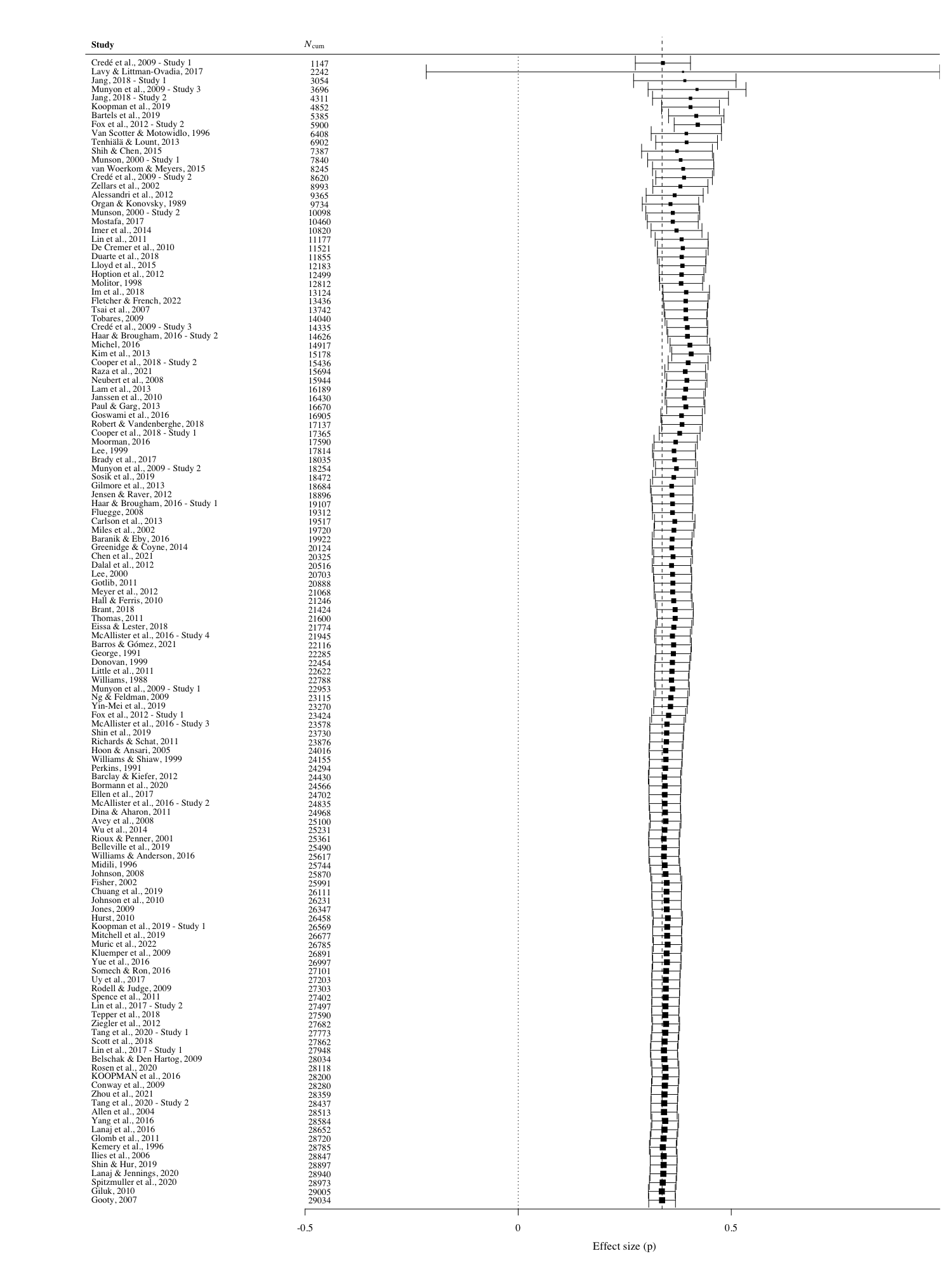 |
| --- |
| Figure S2b. CMA Plot for PA ↔ OCBs |

| 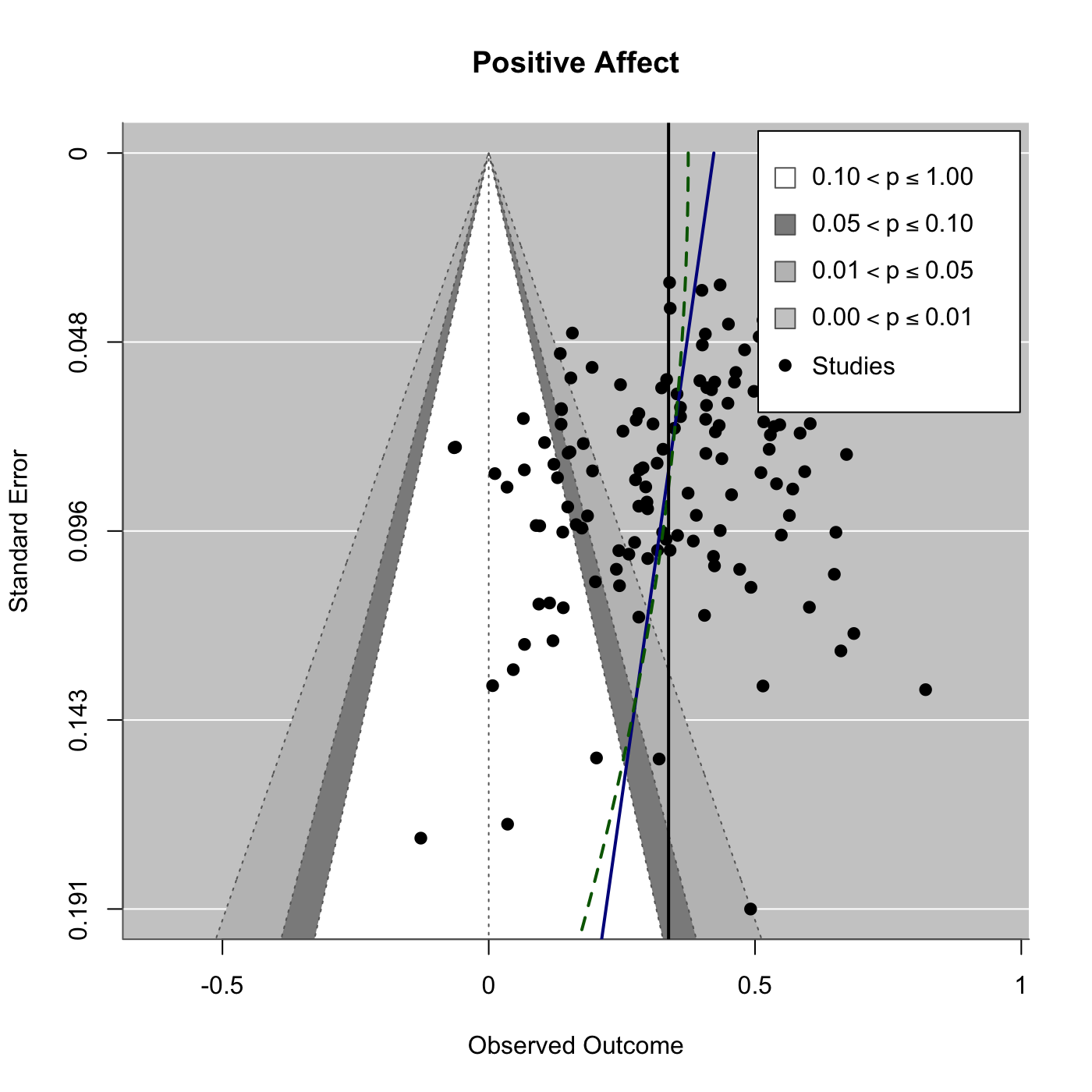 |
| --- |
| Figure S2b. Funnel Plot for PA ↔ OCBs with PET (dark blue, solid), PEESE (dark green, dashed) effect size (black, solid) lines, and estimated confidence 95% CI of effect size |

| 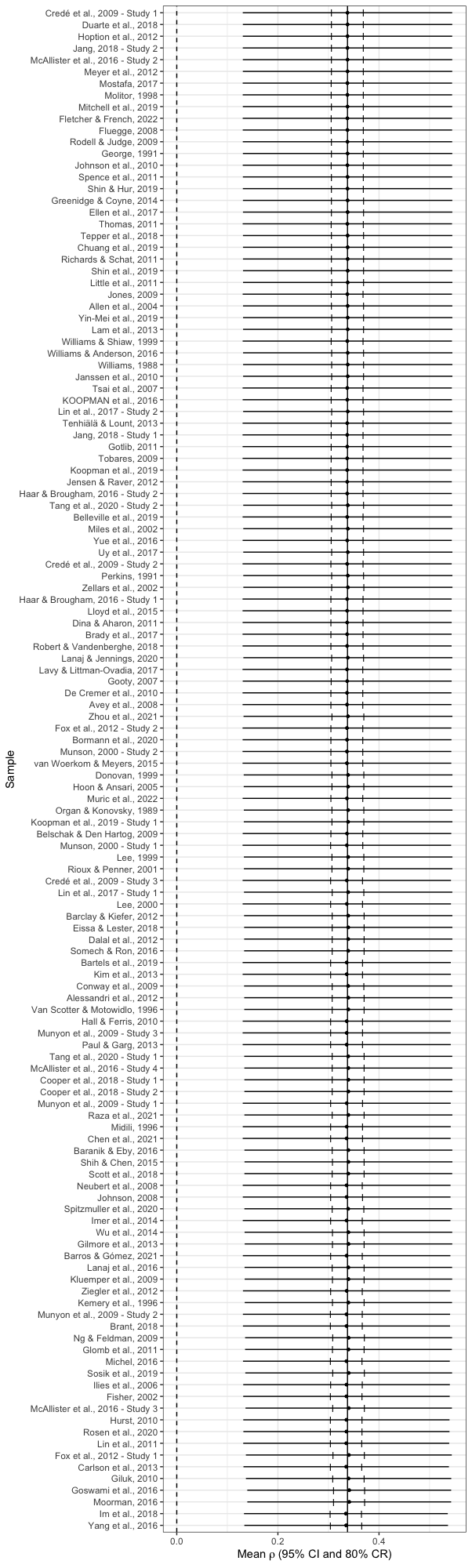 |
| --- |
| Figure S2d. Leave-One-Out Visualization for PA ↔ OCBs |

### Negative Affect

The forest plot for the NA ↔ OCB effects is seen in Figure S3a. For the CMA, there appeared to be a slight drift towards effects with a stronger magnitude (Figure S3b). The largest sample (Credé et al., 2009 - Study 1, *ρ* = .01, *n* = 1,147) did appear to deviate from the final effect size (*ρ* = -.11). This could mean that more precise (as determined by their sample size) results systematically differ from those with smaller sample sizes. This asymmetry in the results is suggestive of some publication bias. The PET-PEESE analysis for Negative Affect was conducted to assess funnel plot asymmetry (Figure S3c). Both the PET (z = 3.63, p < .001) and PEESE (z = 3.66, p < .001) indicated significant funnel plot asymmetry, suggesting the presence of publication bias or small-study effects in the meta-analysis of Negative Affect. Moreover, there appears to be clustering around significant p-values. Notably, the trim-and-fill analysis (Figure S3d) indicates the effect should be more negative (*ρ* = −.15 vs. *ρ* = −.11 from the original analysis), suggesting that the true effect may be *more* negative than originally estimated. Lastly, an inspection of the leave-one-out analysis did not suggest that effects were significantly influenced by any one study.

| 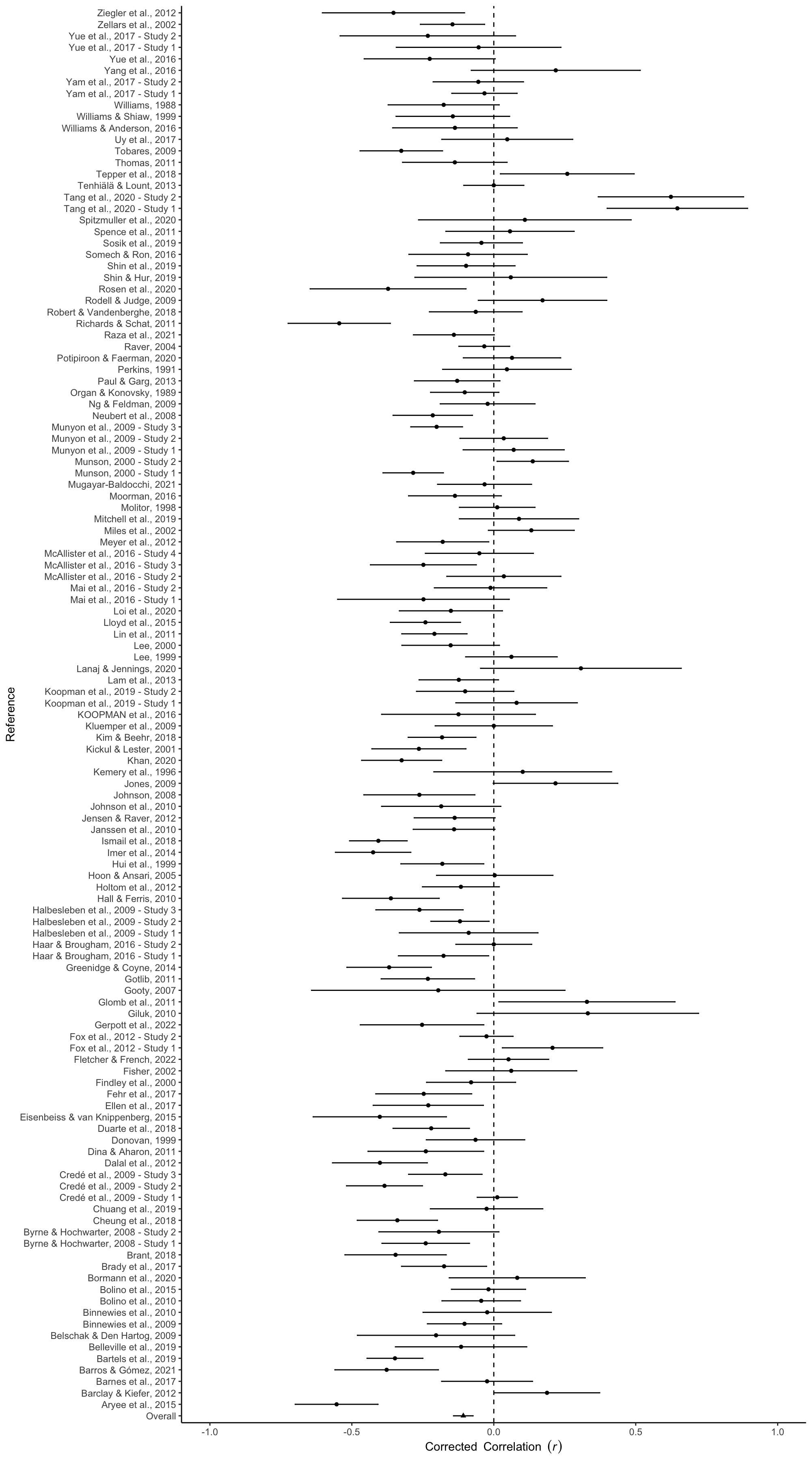 |
| --- |
| Figure S3a. Forest Plot for NA ↔ OCBs |

| 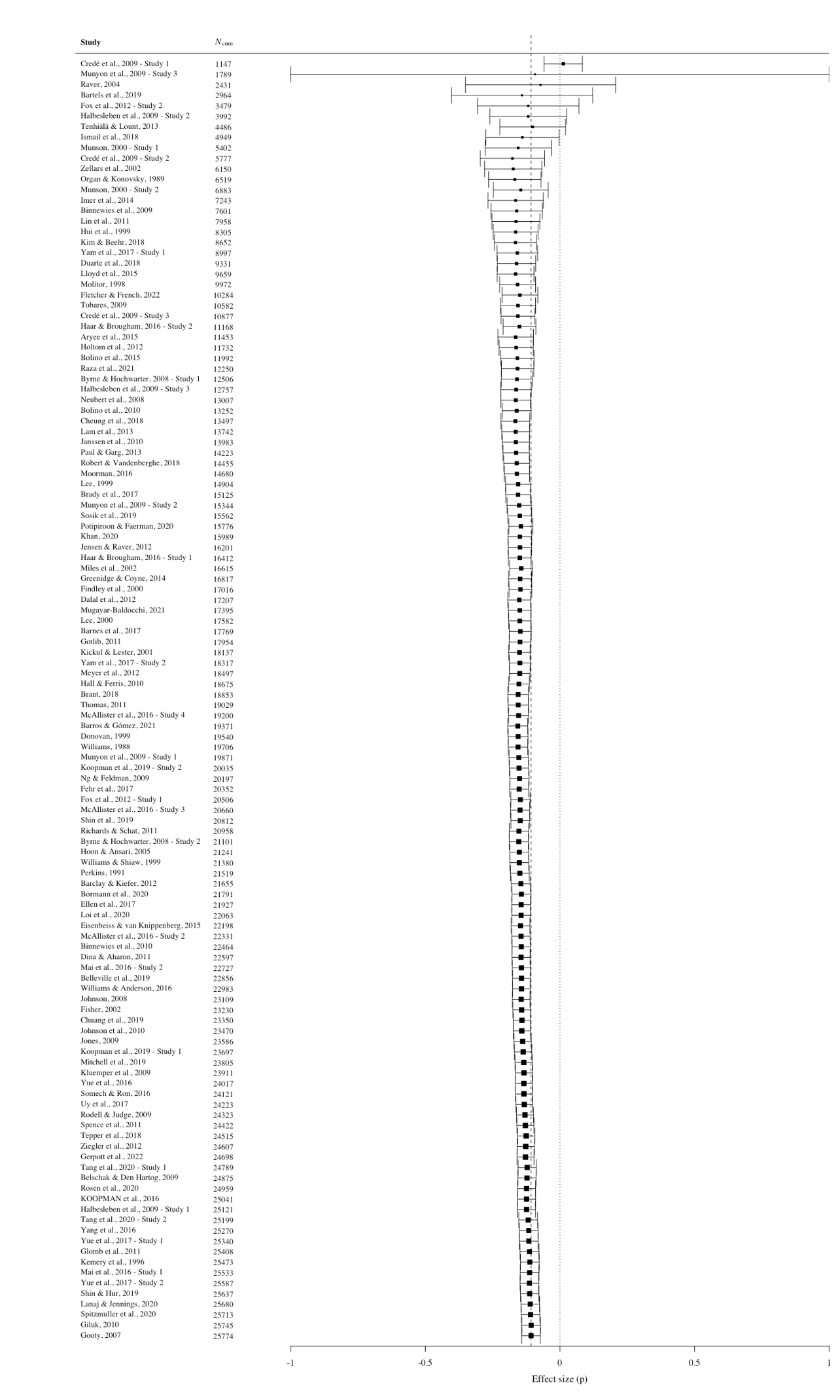 |
| --- |
| Figure S3b. CMA Plot for NA ↔ OCBs |

| 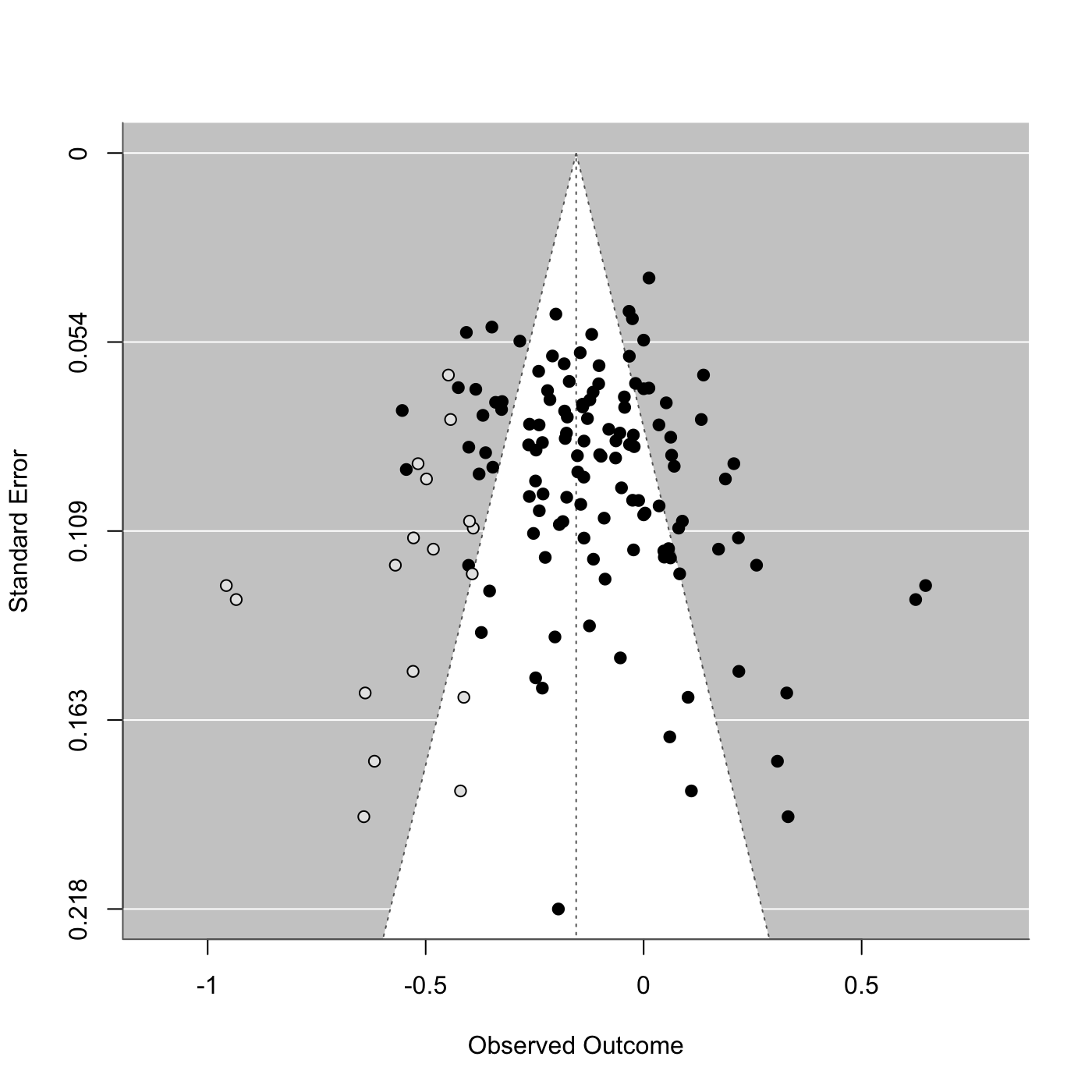 |
| --- |
| Figure S3d. Trim-and-Fill Funnel Plot for NA ↔ OCBs |

| 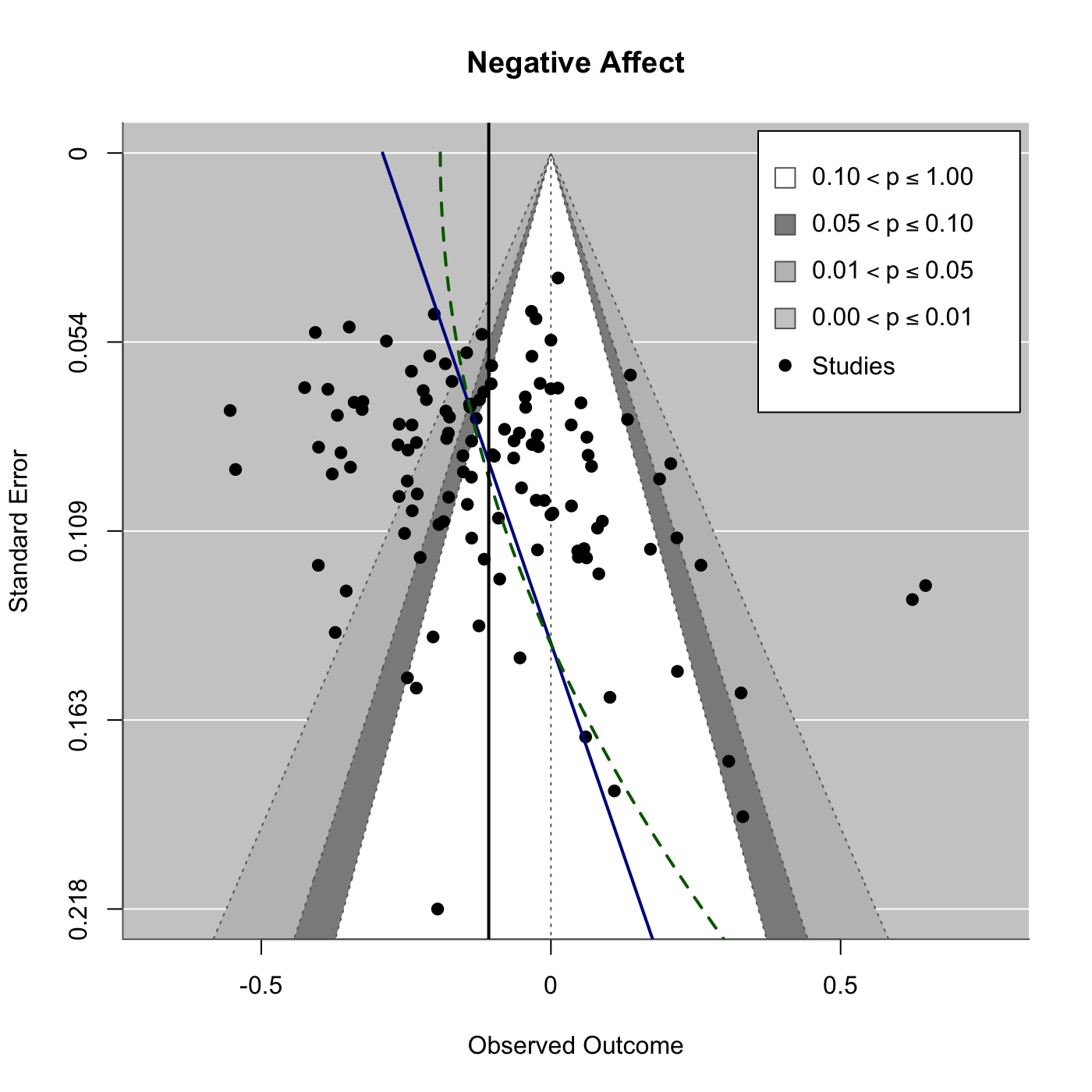 |
| --- |
| Figure S3c. Funnel Plot for NA ↔ OCBs with PET (dark blue, solid), PEESE (dark green, dashed) effect size (black, solid) lines, and estimated confidence 95% CI of effect size |

| 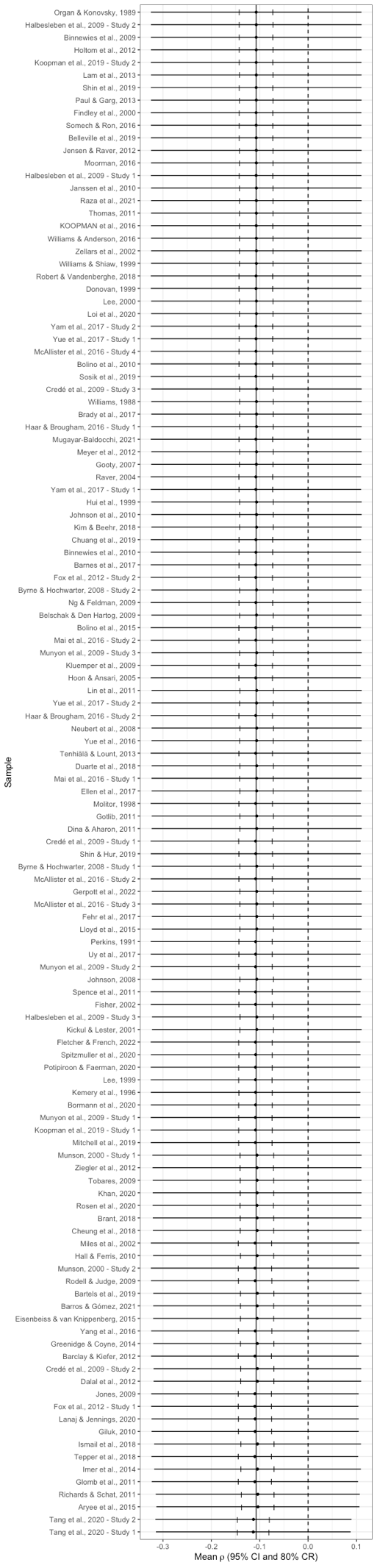 |
| --- |
| Figure S2e. Leave-One-Out Visualization for NA ↔ OCBs |

## Hypothesis 3 – Longitudinal Analysis

Because only a small number of studies were available for the longitudinal meta-analytic results, the robustness and precision of these sensitivity analyses are necessarily limited. We nonetheless conducted them, as the patterns may be of interest to readers who wish to consider additional perspectives on the data.

### Life Satisfaction 🡪 Future OCB

A forest plot of the effects is seen in Figure S4a. The results from the CMA can be seen in Figure S4b, with the study having the largest sample (Luan et al., 2022; *ρ* = .17, *n* = 209) not being distinct from the overall effect size (*ρ* = .16). The PET-PEESE analysis for Life Satisfaction predicting future OCBs was conducted to assess funnel plot asymmetry. Neither the PET (*z* = 0.04, p > .05) nor PEESE (*z* = 0.04, p > .05) indicated significant funnel plot asymmetry. Moreover, the contour-enhanced funnel plot (Figure S4c) did not indicate clustering within that significant p-value range. Lastly, a visualization of the one-sample removed results can be seen in Figure S4d.

| 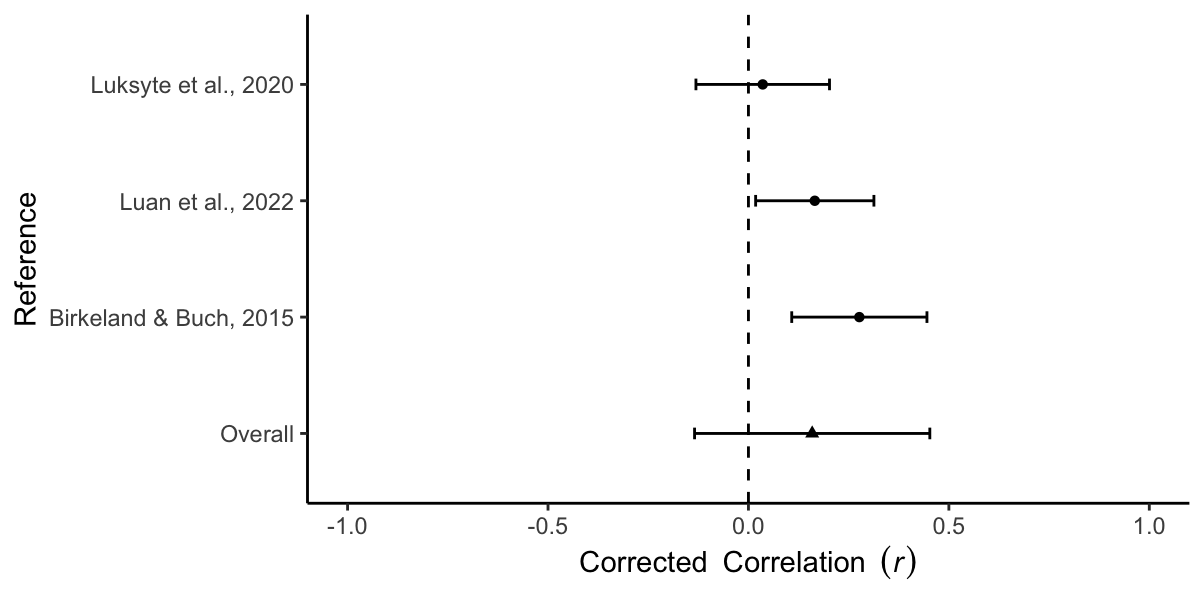 |
| --- |
| Figure S4a. Forest Plot for LS 🡪 Future OCBs |

| 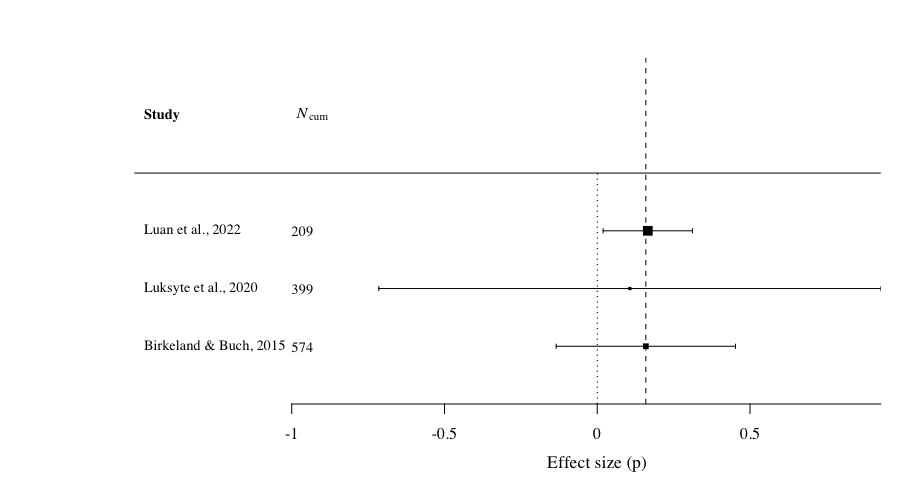 |
| --- |
| Figure S4b. CMA Plot for LS 🡪 Future OCBs |

| 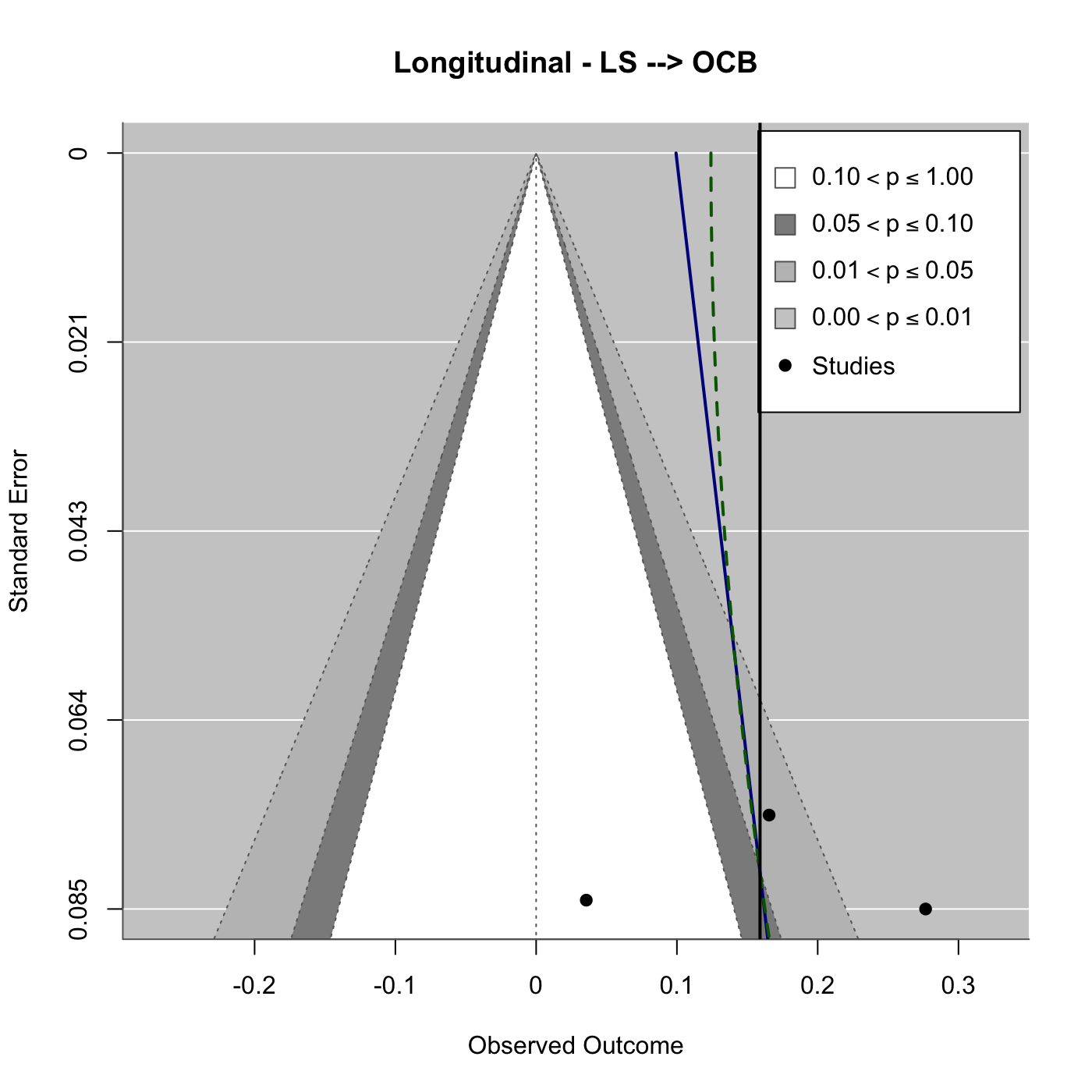 |
| --- |
| Figure S4c. Funnel Plot for LS 🡪 Future OCBs with PET (dark blue, solid), PEESE (dark green, dashed) effect size (black, solid) lines, and estimated confidence 95% CI of effect size |

| 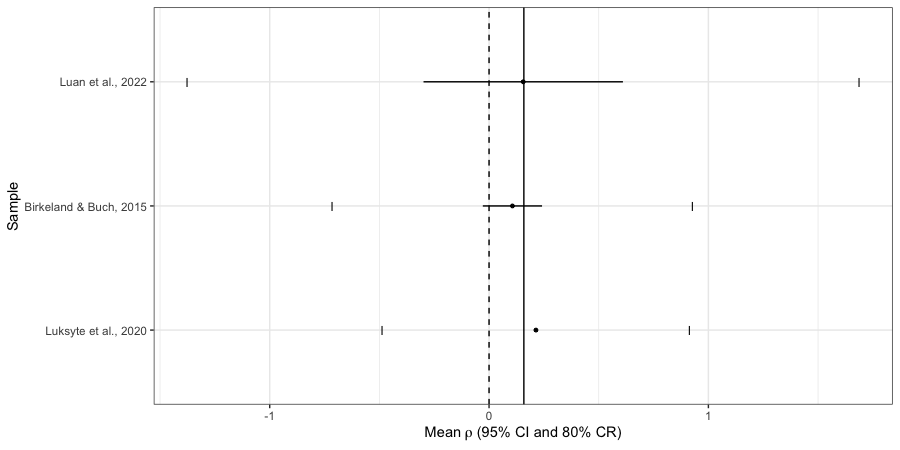 |
| --- |
| Figure S4d. Leave-One-Out Visualization |

### Positive Affect 🡪 Future OCB

A forest plot of the effects is seen in Figure S5a. The results from the CMA can be seen in Figure S5b, with the study having the largest sample (Jang, 2018 - Study 2; *ρ* = .34, *n* = 614) not being distinct from the overall effect size (*ρ* = .39). The PET-PEESE analysis for Positive Affect predicting future OCBs was conducted to assess funnel plot asymmetry. Neither the PET (*z* = -1.37, p > .05) nor PEESE (*z* = -1.88, p > .05) indicated significant funnel plot asymmetry, suggesting no publication bias or small-study effects in the meta-analysis of Positive Affect predicting future OCBs. Moreover, the contour-enhanced funnel plot (Figure S5c) did not indicate clustering within that significant p-value range. Lastly, a visualization of the one-sample removed results can be seen in Figure S5d.

| 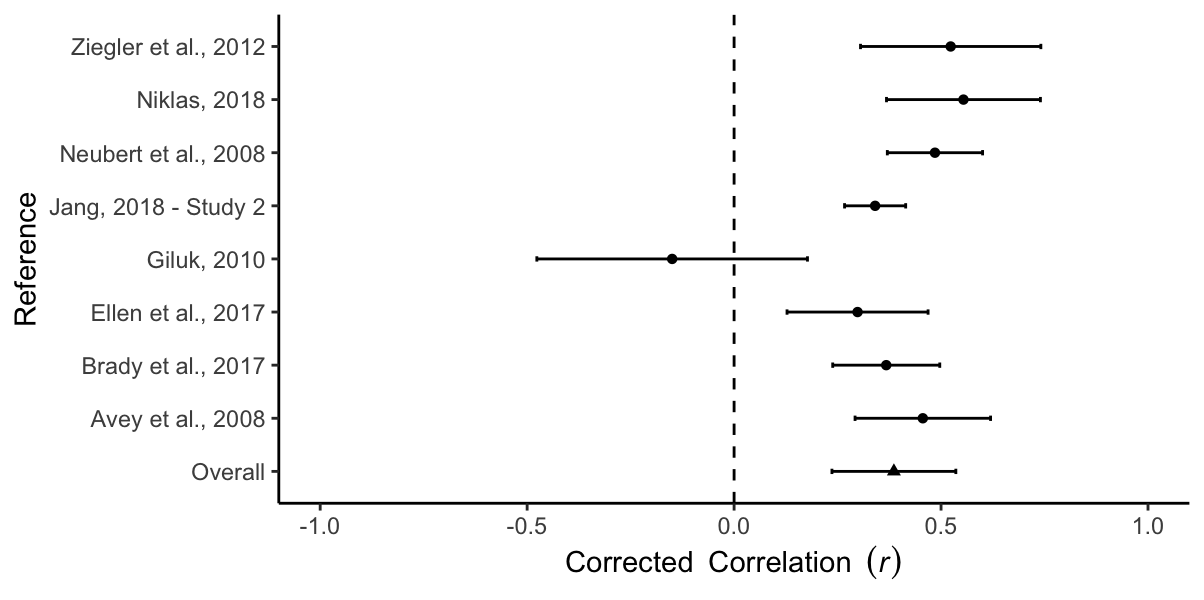 |
| --- |
| Figure S5a. Forest Plot for PA 🡪 Future OCBs |

| 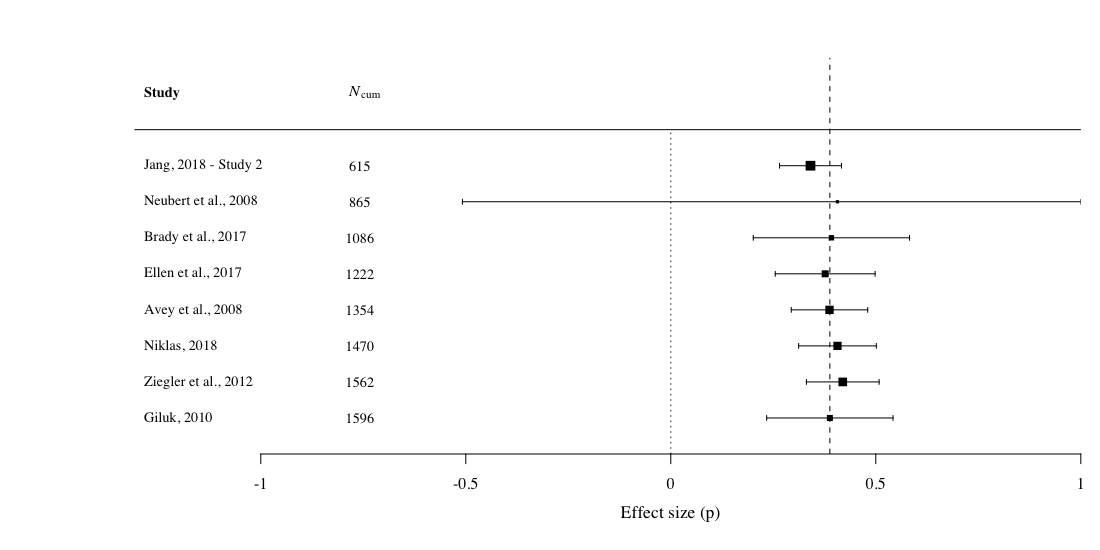 |
| --- |
| Figure S5b. CMA Plot for PA 🡪 Future OCBs |

| 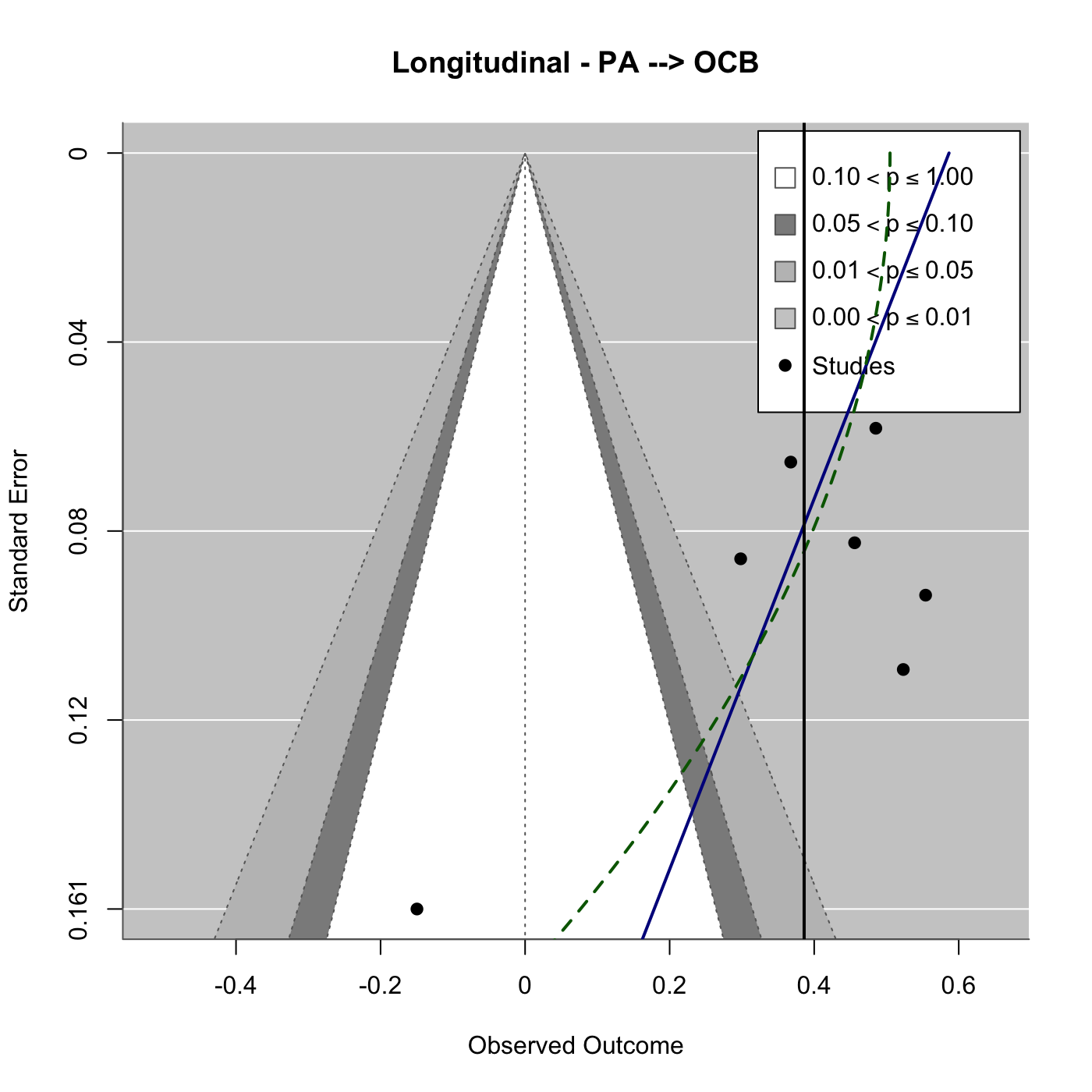 |
| --- |
| Figure S5b. Funnel Plot for PA 🡪 Future OCBs with PET (dark blue, solid), PEESE (dark green, dashed) effect size (black, solid) lines, and estimated confidence 95% CI of effect size |

| 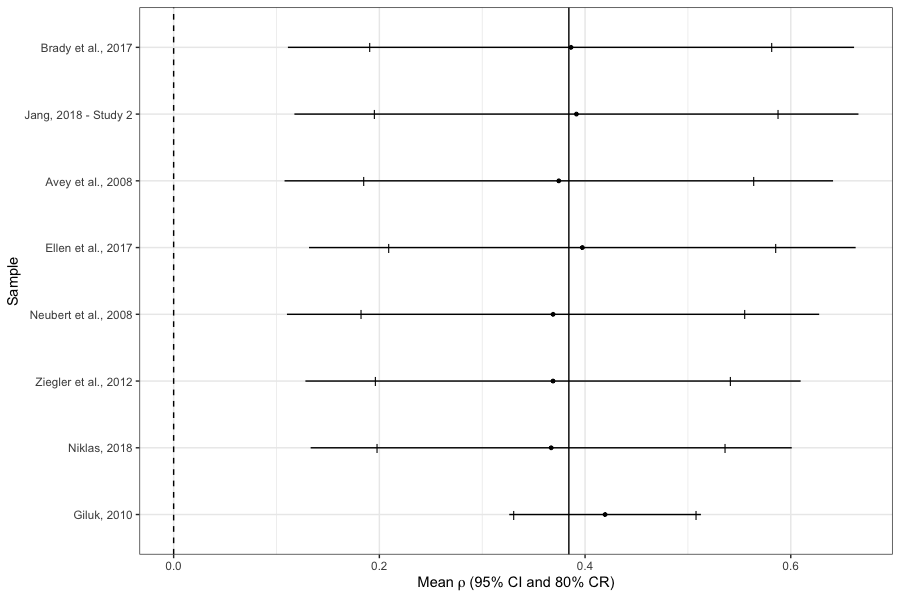 |
| --- |
| Figure S5d. Leave-One-Out Visualization |

### Negative Affect 🡪 Future OCB

A forest plot of the effects is seen in Figure S6a. The results from the CMA can be seen in Figure S6b, with the study having the largest sample (Halbesleben et al., 2009 - Study 2; *ρ* = -.11, *n* = 513) not being distinct from the overall effect size (*ρ* = -.13). The PET-PEESE analysis for Negative Affect predicting future OCBs was conducted to assess funnel plot asymmetry. Neither the PET (*z* = -0.38, *p* > .05) nor PEESE (*z* = 0.06, *p* > .05) indicated significant funnel plot asymmetry. Moreover, the contour-enhanced funnel plot (Figure S6c) did not indicate clustering within that significant p-value range. Lastly, a visualization of the one-sample removed results can be seen in Figure S6d.

| 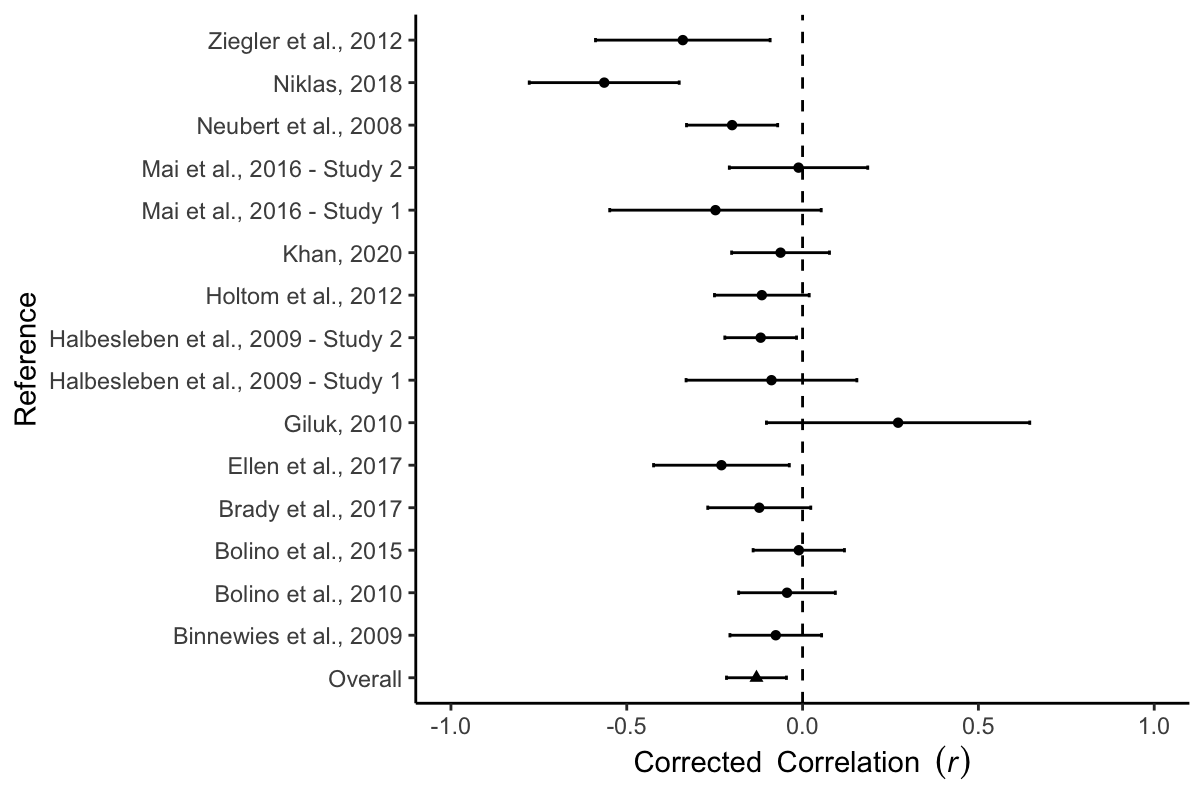 |
| --- |
| Figure S6a. Forest Plot for NA 🡪 Future OCBs |

| 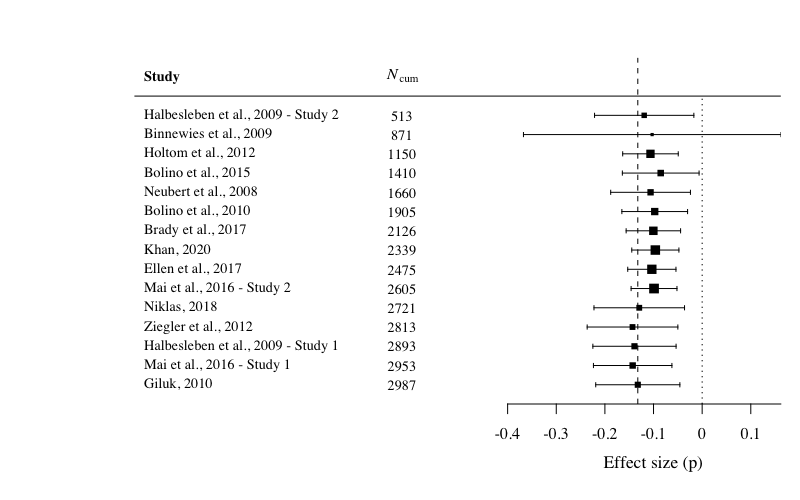 |
| --- |
| Figure S6b. CMA Plot for NA 🡪 Future OCBs |

| 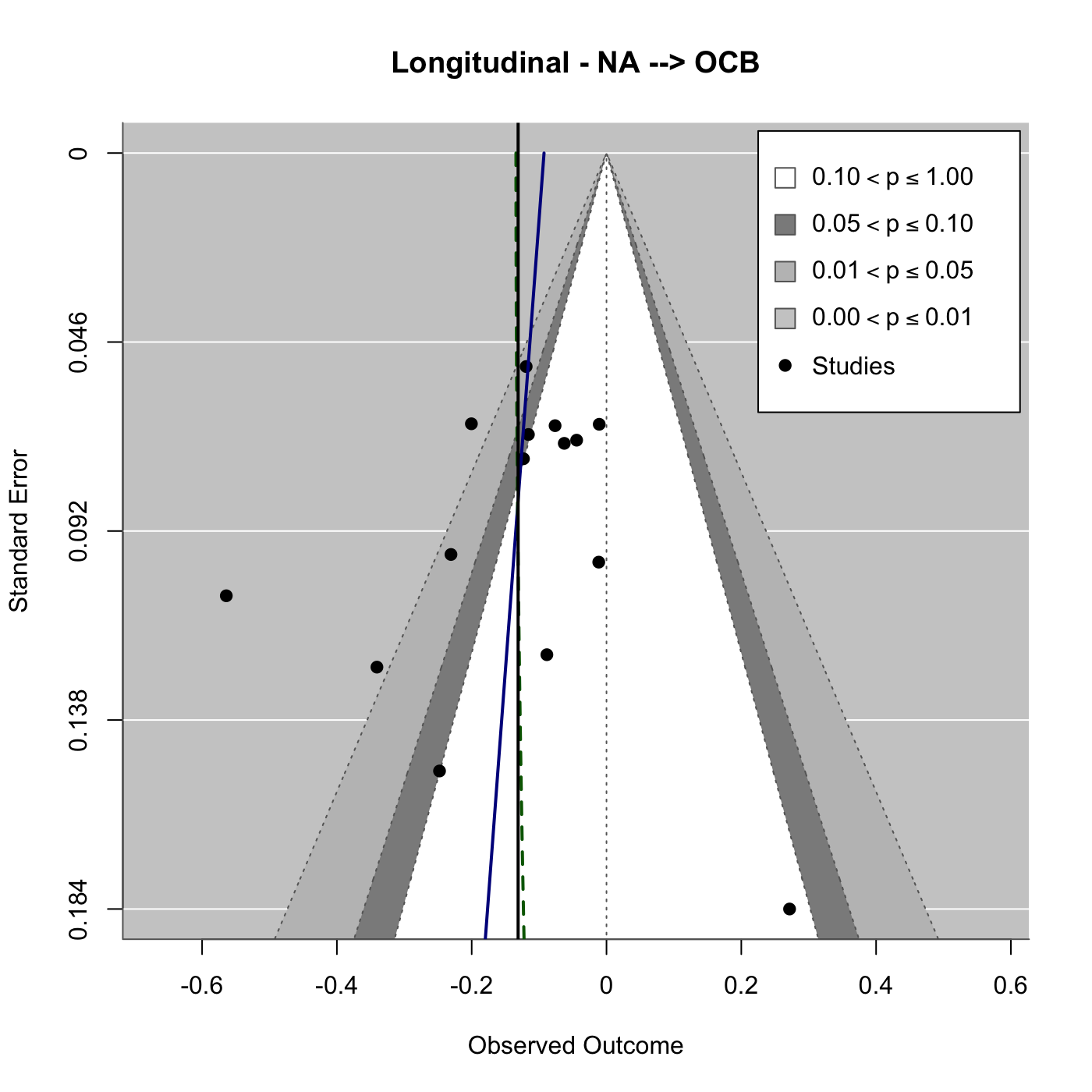 |
| --- |
| Figure S6c. Funnel Plot for NA 🡪 Future OCBs with PET (dark blue, solid), PEESE (dark green, dashed) effect size (black, solid) lines, and estimated confidence 95% CI of effect size |

| 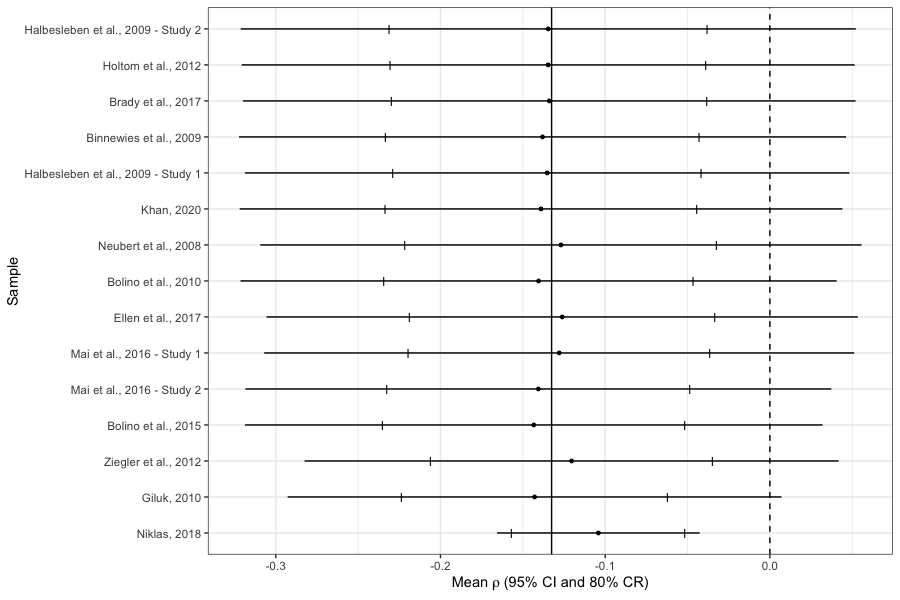 |
| --- |
| Figure S6d. Leave-One-Out Visualization |

### OCB 🡪 Future Positive Affect

A forest plot of the effects is seen in Figure S7a. The results from the CMA can be seen in Figure S7b, with the study having the largest sample (Mostafa, 2017; *ρ* = .31, *n* = 362) not being distinct from the overall effect size (*ρ* = .20). The PET-PEESE analysis for OCBs predicting future Positive Affect was conducted to assess funnel plot asymmetry. Neither the PET (z = -1.32, p > .05) nor PEESE (z = -1.44, p > .05) indicated significant funnel plot asymmetry (Fogire S7c). Lastly, a visualization of the one-sample removed results can be seen in Figure S7d.

| 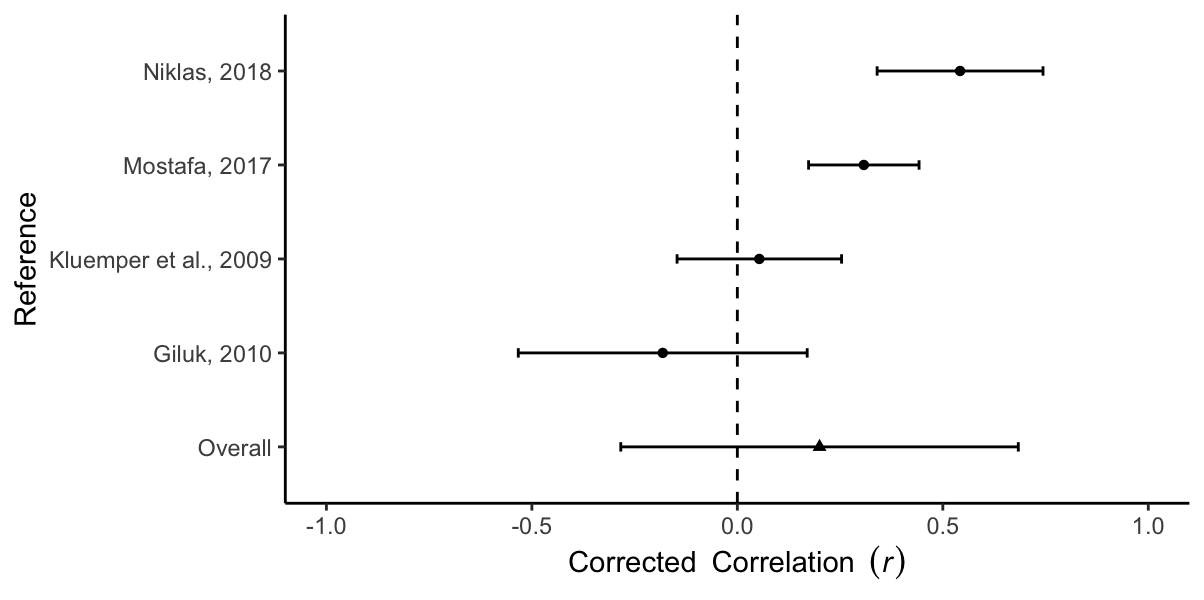 |
| --- |
| Figure S7a. Forest Plot for OCBs 🡪 Future PA |

| 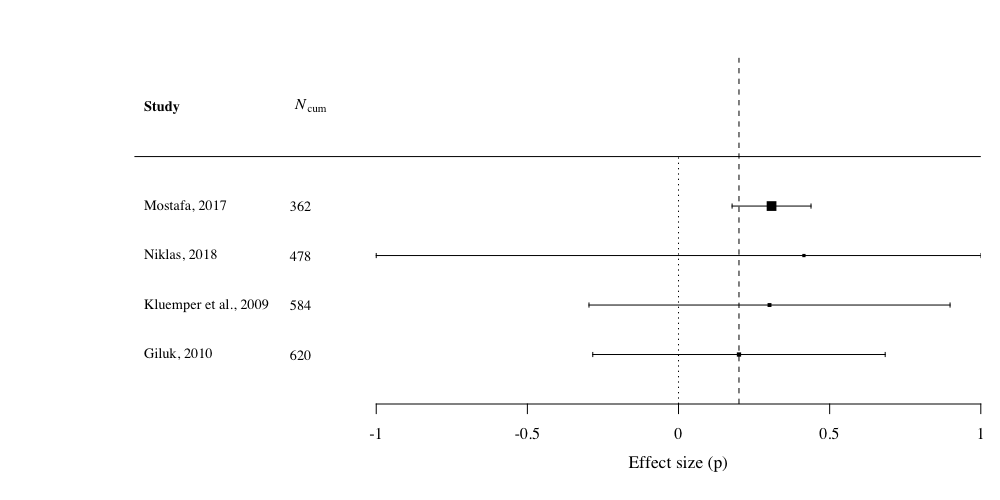 |
| --- |
| Figure S7b. CMA Plot for OCBs 🡪 Future PA |

| 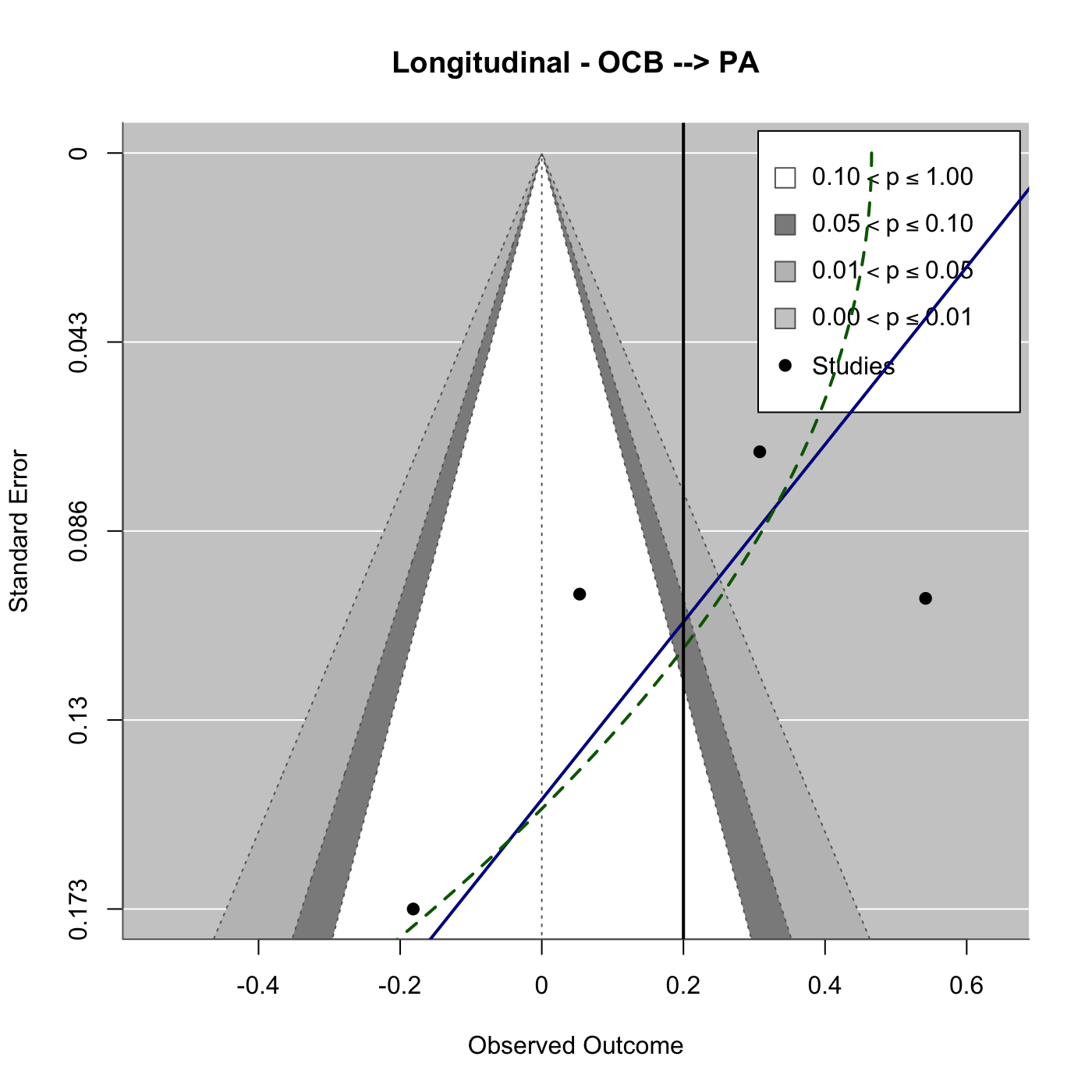 |
| --- |
| Figure S7c. Funnel Plot for OCBs 🡪 Future PA with PET (dark blue, solid), PEESE (dark green, dashed) effect size (black, solid) lines, and estimated confidence 95% CI of effect size |

| 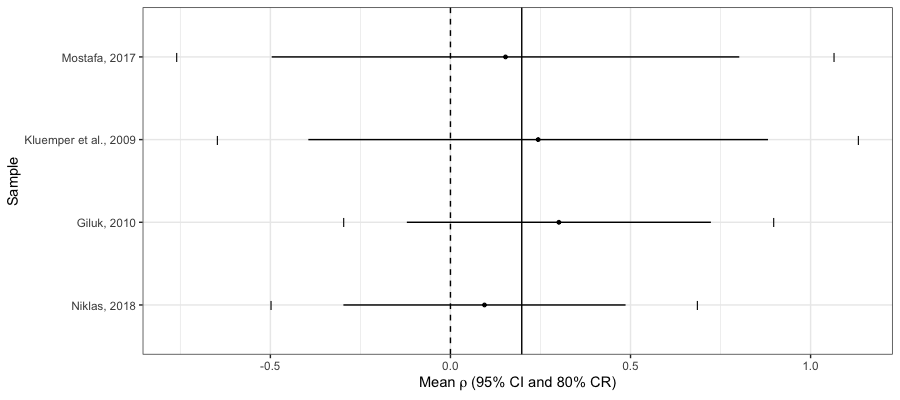 |
| --- |
| Figure S7d. Leave-One-Out Visualization |

### OCB 🡪 Future Negative Affect

A forest plot of the effects is seen in Figure S8a. The results from the CMA can be seen in Figure S8b, with the study having the largest sample (Niklas, 2018; *ρ* = -.41, *n* = 116) not able to be compared to be distinct or not with the overall effect size (*ρ* = -.06). The PET-PEESE analysis for OCBs predicting future Negative Affect was conducted to assess funnel plot asymmetry. Neither the PET (z = 1.06, p > .05) nor PEESE (z = 1.06, p > .05) indicated significant funnel plot asymmetry (Figure S8c). Lastly, a visualization of the one-sample removed results can be seen in Figure S8d.

| 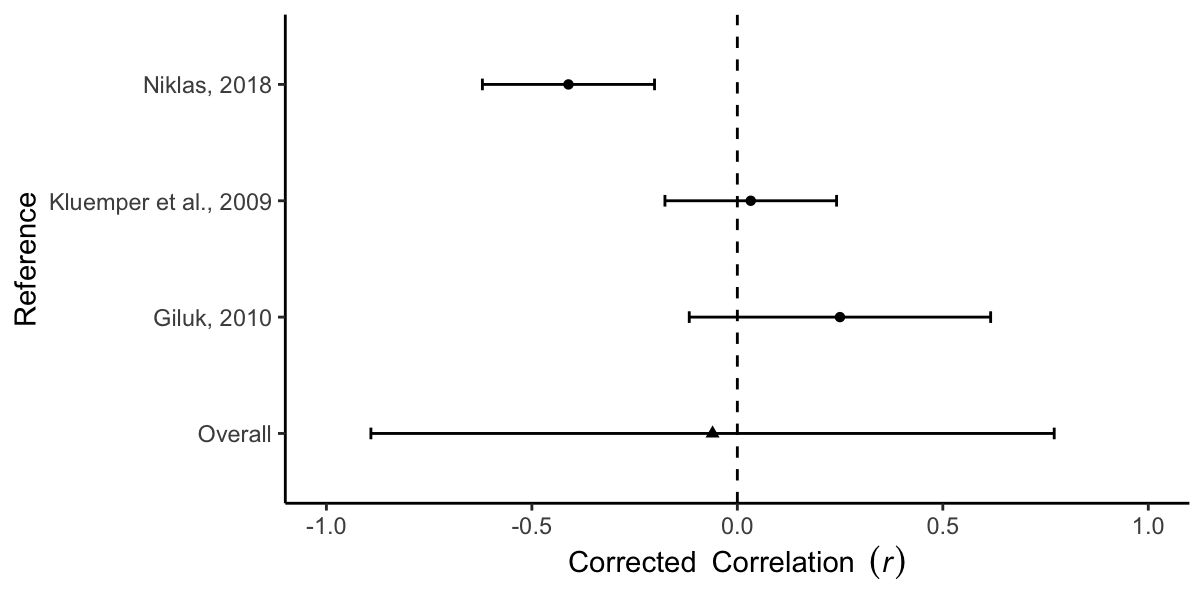 |
| --- |
| Figure S8a. Forest Plot for NA 🡪 Future OCBs |

| 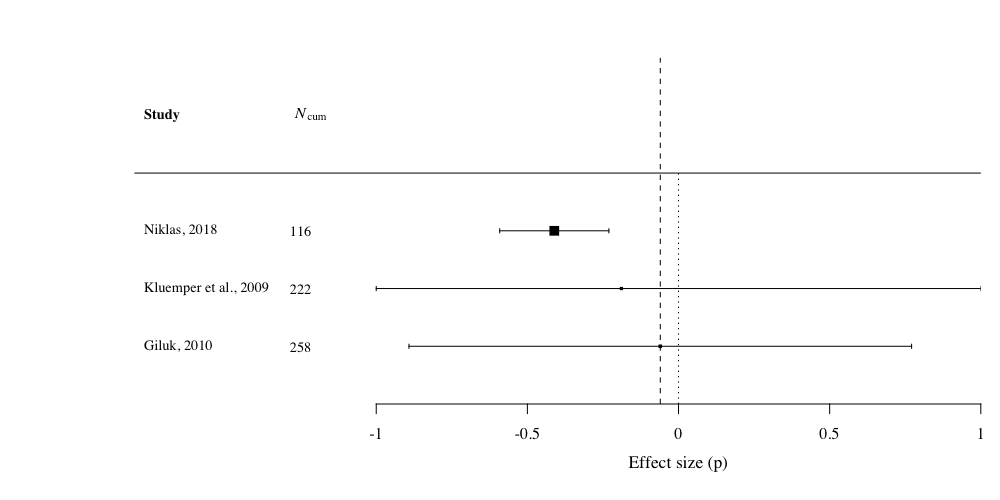 |
| --- |
| Figure S8b. CMA Plot for NA 🡪 Future OCBs |

| 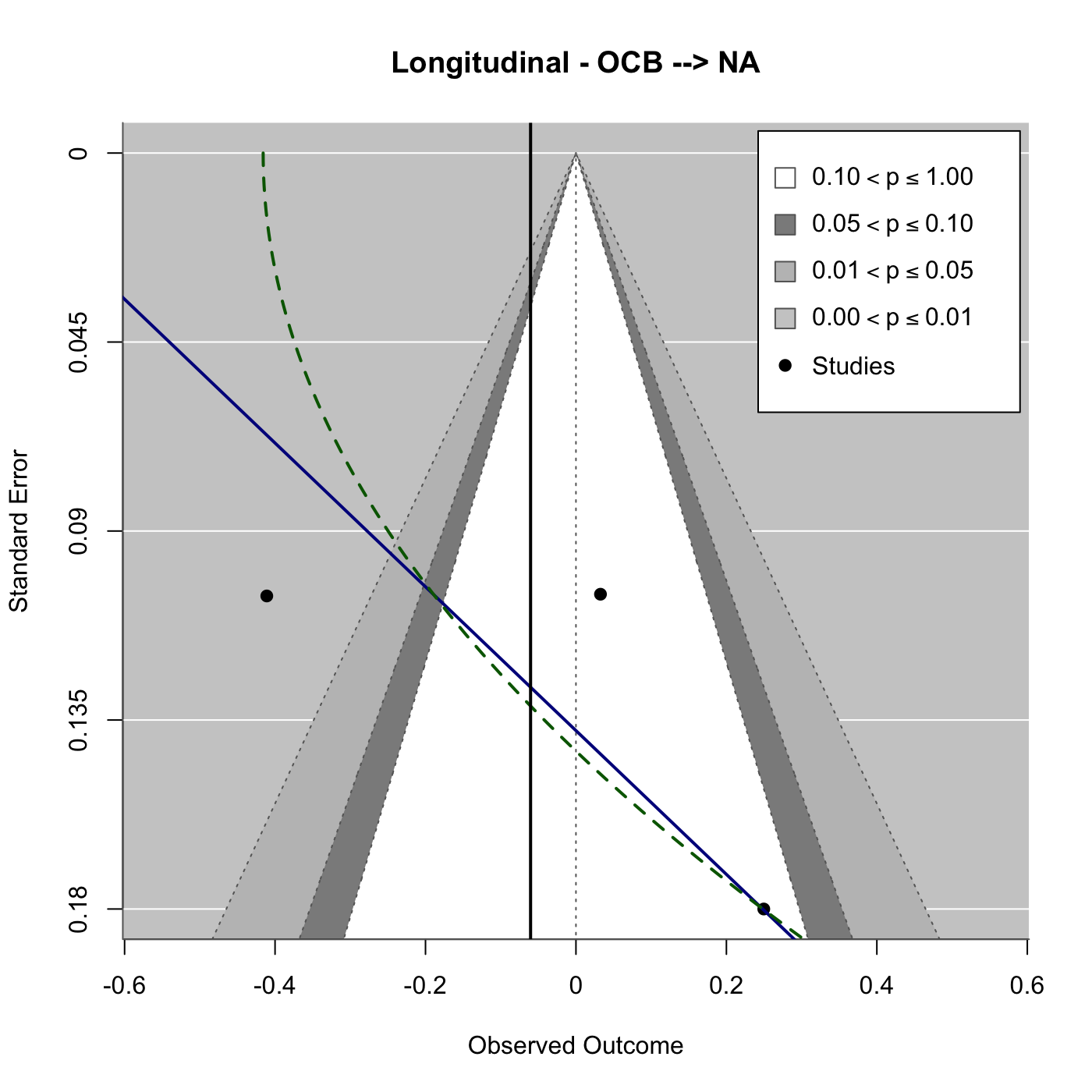 |
| --- |
| Figure S8c. Funnel Plot for NA 🡪 Future OCBs with PET (dark blue, solid), PEESE (dark green, dashed) effect size (black, solid) lines, and estimated confidence 95% CI of effect size |

| 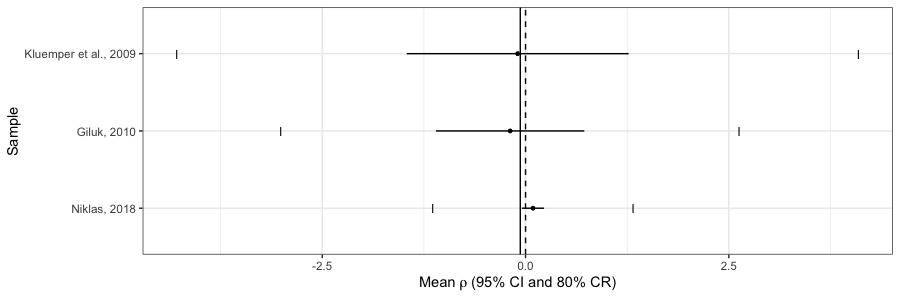 |
| --- |
| Figure S8d. Leave-One-Out Visualization |

## Hypothesis 4 – Within-Person Analysis

###

### Positive Affect

A forest plot of the effects is seen in Figure S9a. The results from the CMA can be seen in Figure S9b, with the study having the largest sample (Koopman et al., 2019 - Study 1; *ρ* = .14, *n* = 1254) not seemingly deviating from the overall effect size (*ρ* = .18), nor did it appear to have significant drift. The PET-PEESE analysis for Positive Affect (within-person) was conducted to assess funnel plot asymmetry (Figure S9c). Neither the PET (z = 0.57, p > .05) nor PEESE (z = 0.40, p > .05) indicated significant funnel plot asymmetry, suggesting no publication bias or small-study effects in the meta-analysis of Positive Affect within-person. Moreover, the contour-enhanced funnel plot did not indicate that studies were clustering within that significant p-value range, indicating a lack of significant p-hacking in the results. Lastly, a visualization of the one-sample removed results can be seen in Figure S9d, with no sample drastically altering the effect size estimates.

| 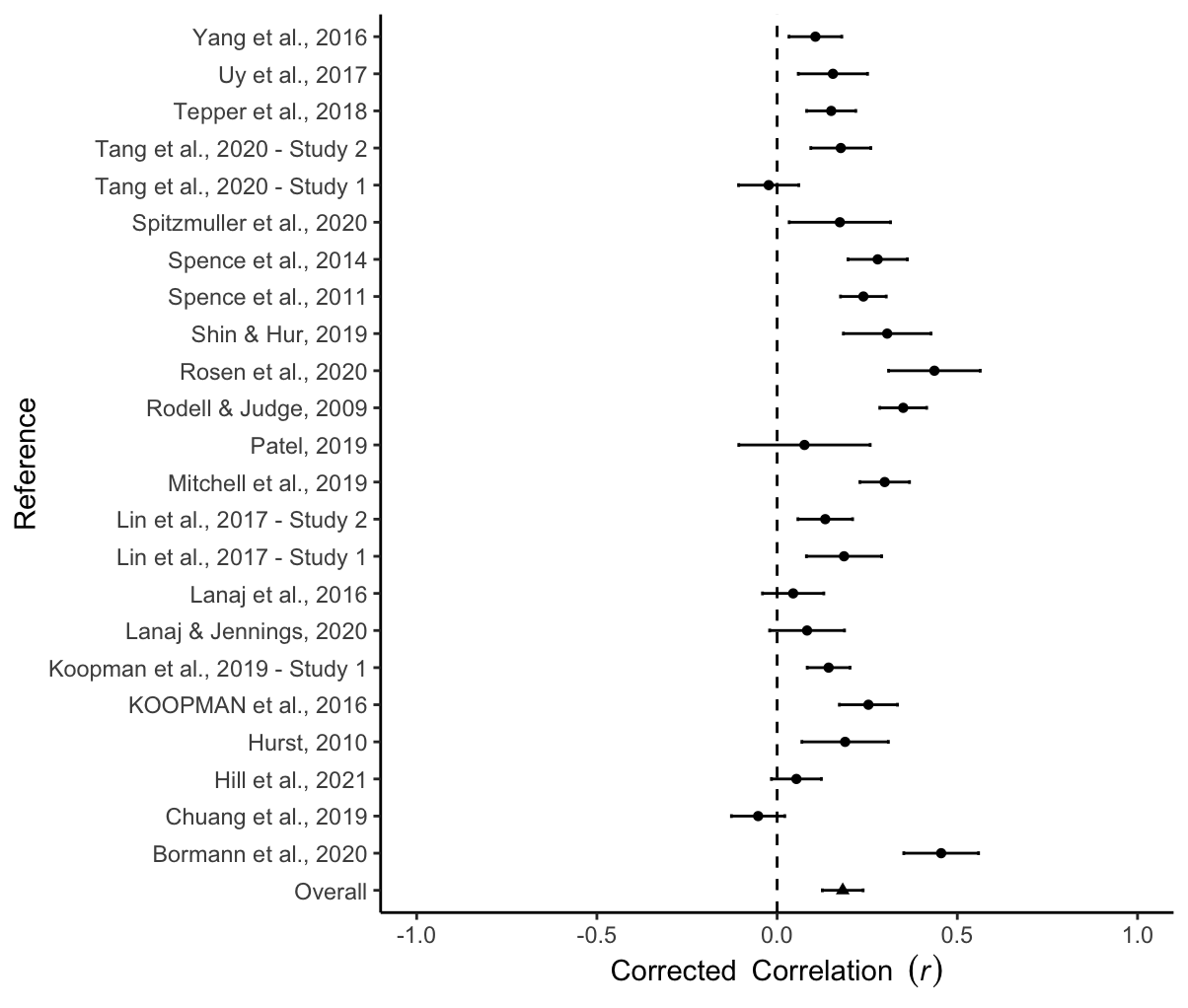 |
| --- |
| Figure S9a. Forest Plot for PA ↔ OCBs at within-person level |

| 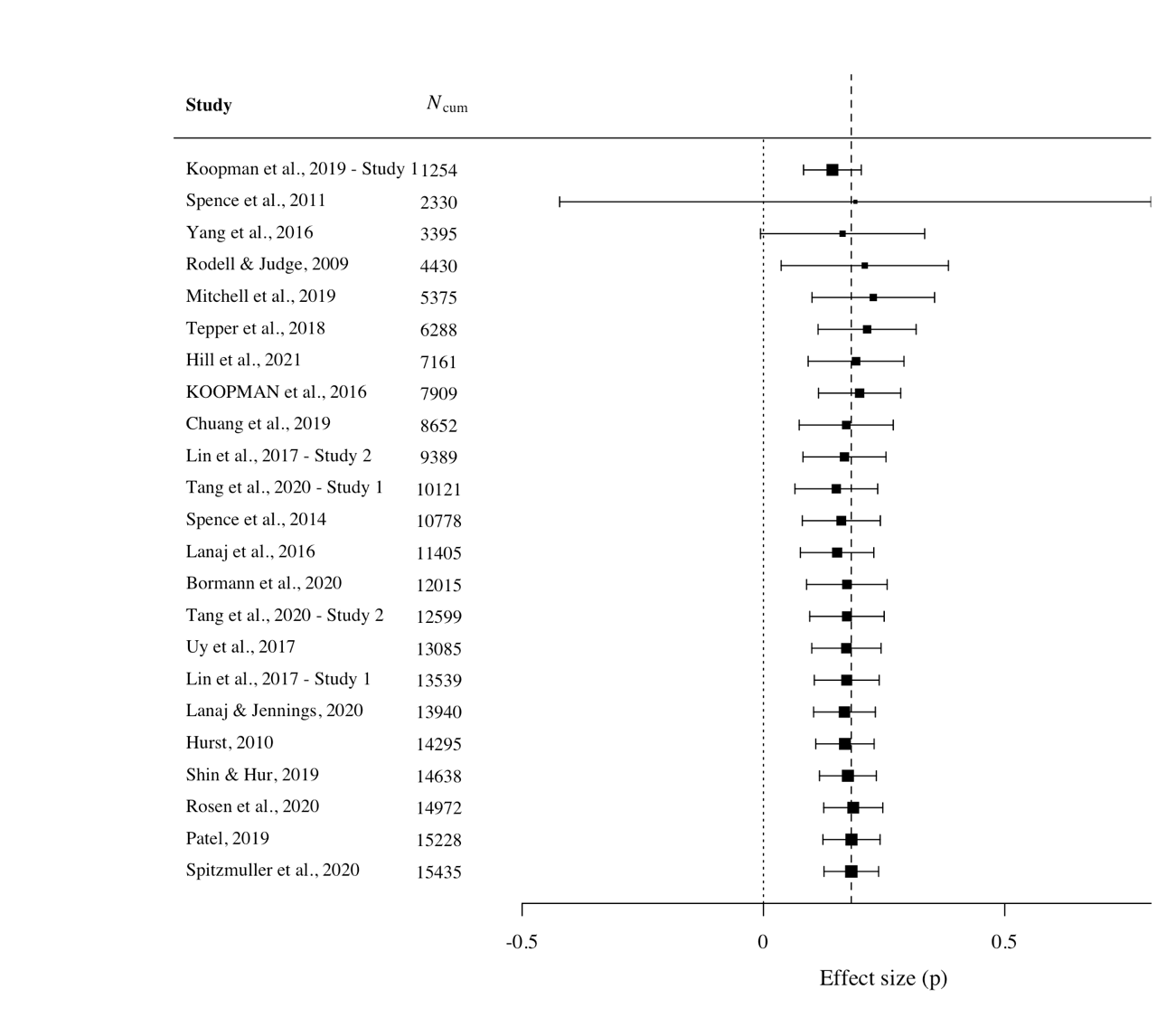 |
| --- |
| Figure S9b. CMA Plot for PA ↔ OCBs (Within) |

| 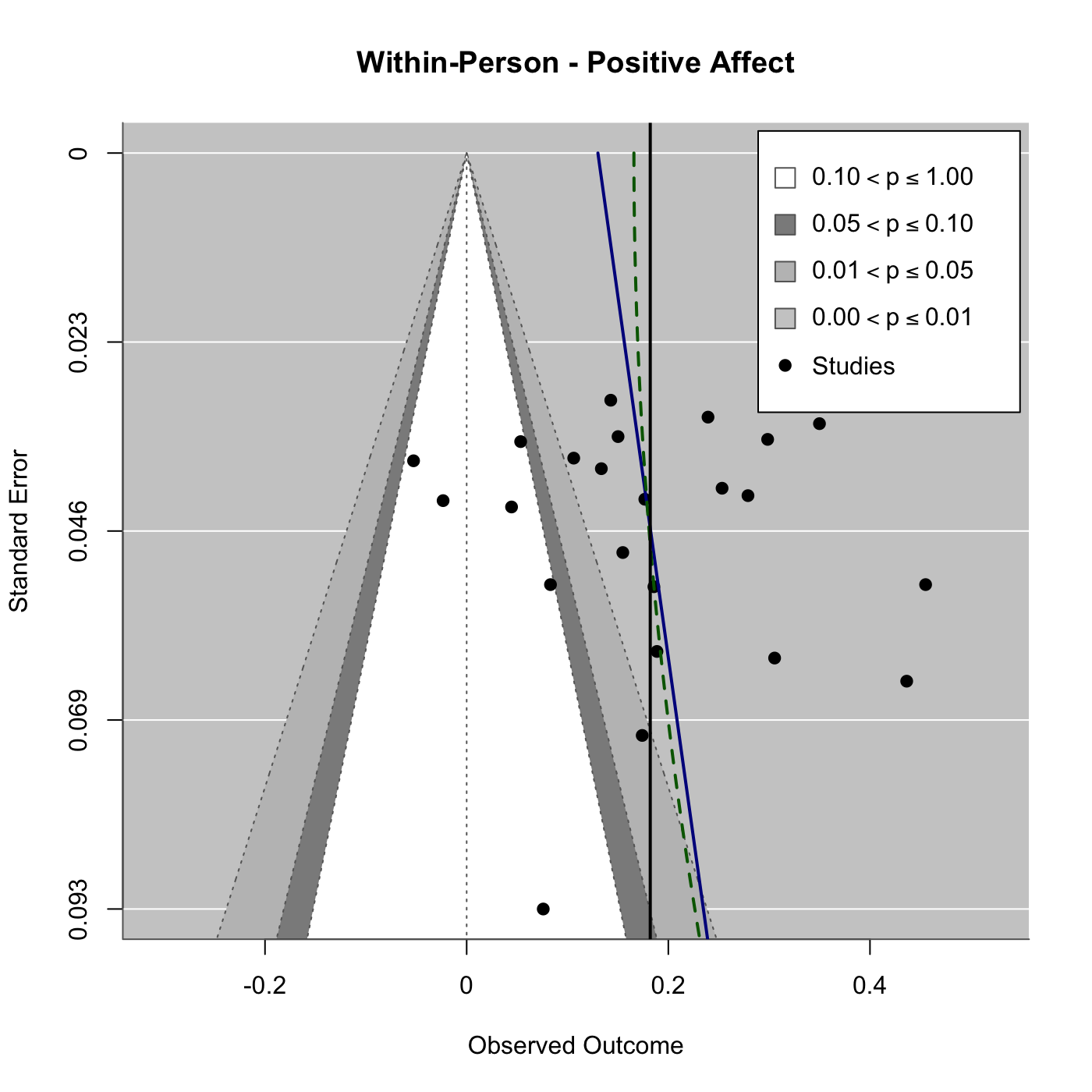 |
| --- |
| Figure S9c. Funnel Plot for PA ↔ OCBs for within-person analysis with PET (dark blue, solid), PEESE (dark green, dashed) effect size (black, solid) lines, and estimated confidence 95% CI of effect size |

| 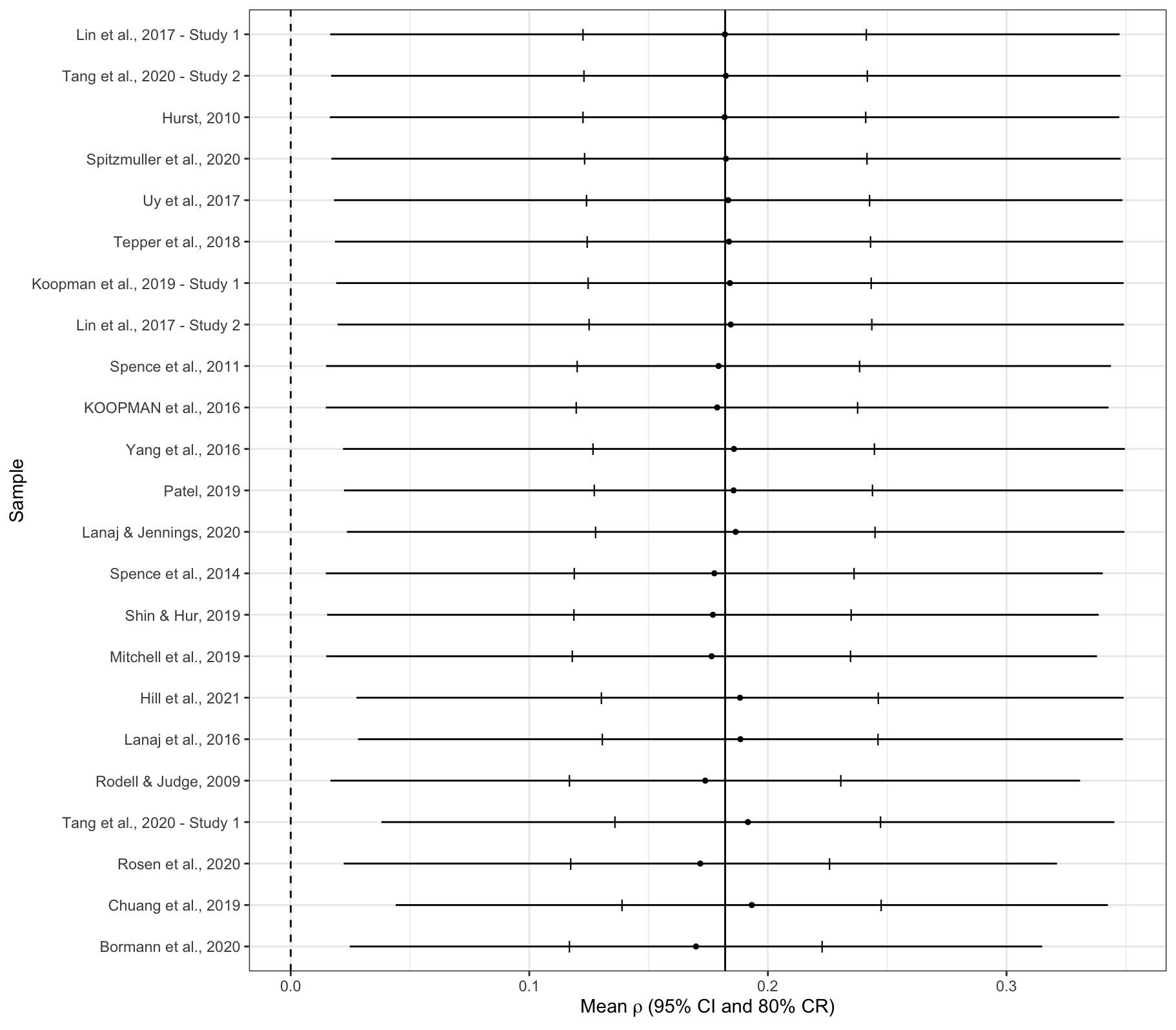 |
| --- |
| Figure S9d. Leave-One-Out Visualization for PA ↔ OCBs (Within) |

### Negative Affect

A forest plot of the effects is seen in Figure S10a. The results from the CMA can be seen in Figure S10b, with the study having the largest sample (Fehr et al., 2017; *ρ* = -.20 *n* = 1532), which differed drastically from the final overall effect size (*ρ* = .05). Moreover, a visual inspection of the CMA plot suggests that significant drift is occurring towards a null effect. The PET-PEESE analysis for Negative Affect within-person was conducted to assess funnel plot asymmetry (Figure S10c). Neither the PET (z = 0.48, p > .05) nor PEESE (z = 0.09, p > .05) indicated significant funnel plot asymmetry, suggesting no publication bias or small-study effects in the meta-analysis of Negative Affect within-person. Lastly, a visualization of the one-sample removed results can be seen in Figure S10d, with no sample drastically altering the effect size estimates. Notably, the removal of Fehr et al. (2017) increased the effect size estimate to *ρ* = .06.

| 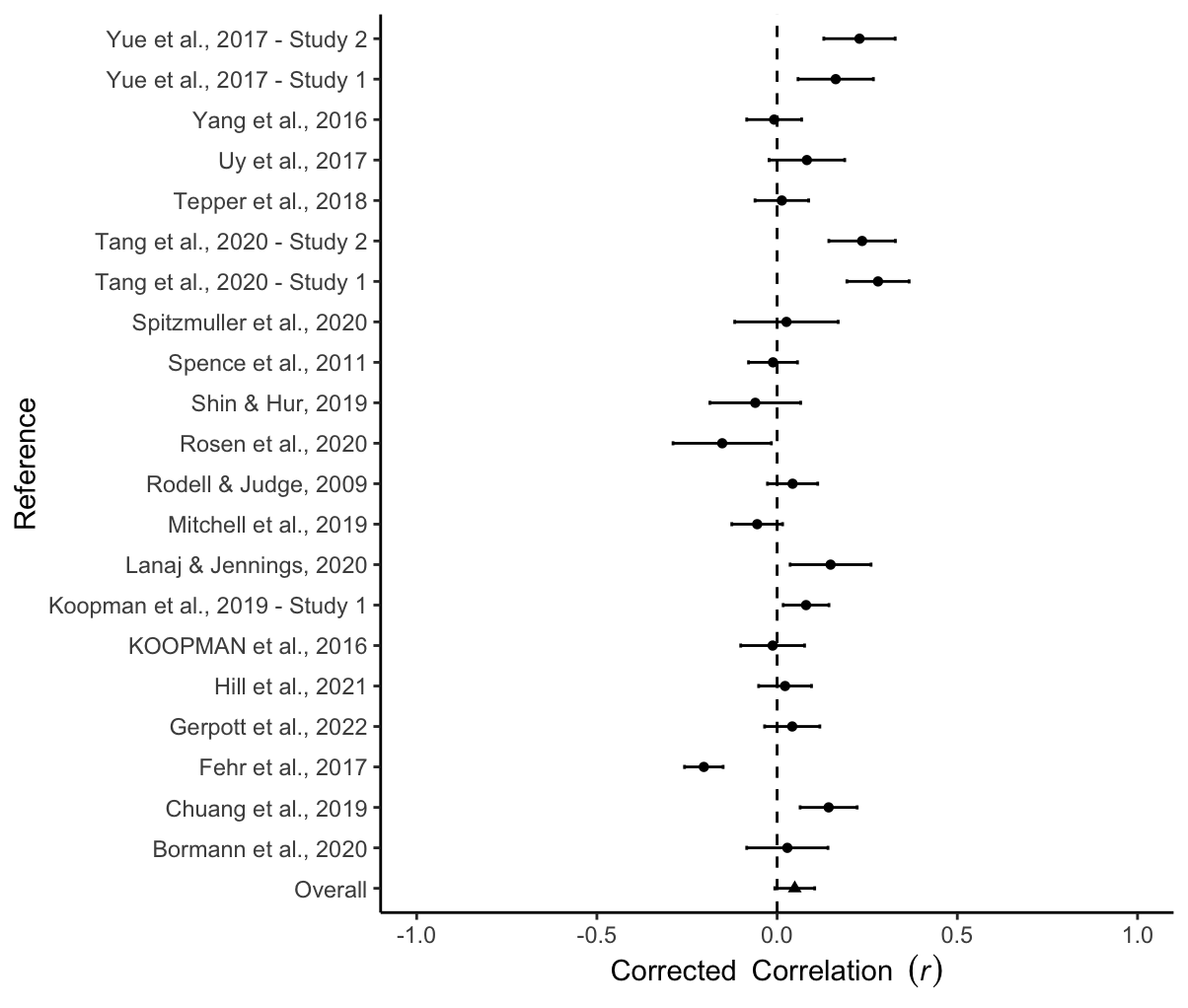 |
| --- |
| Figure S10a. Forest Plot for NA ↔ OCBs at within-person level |

| 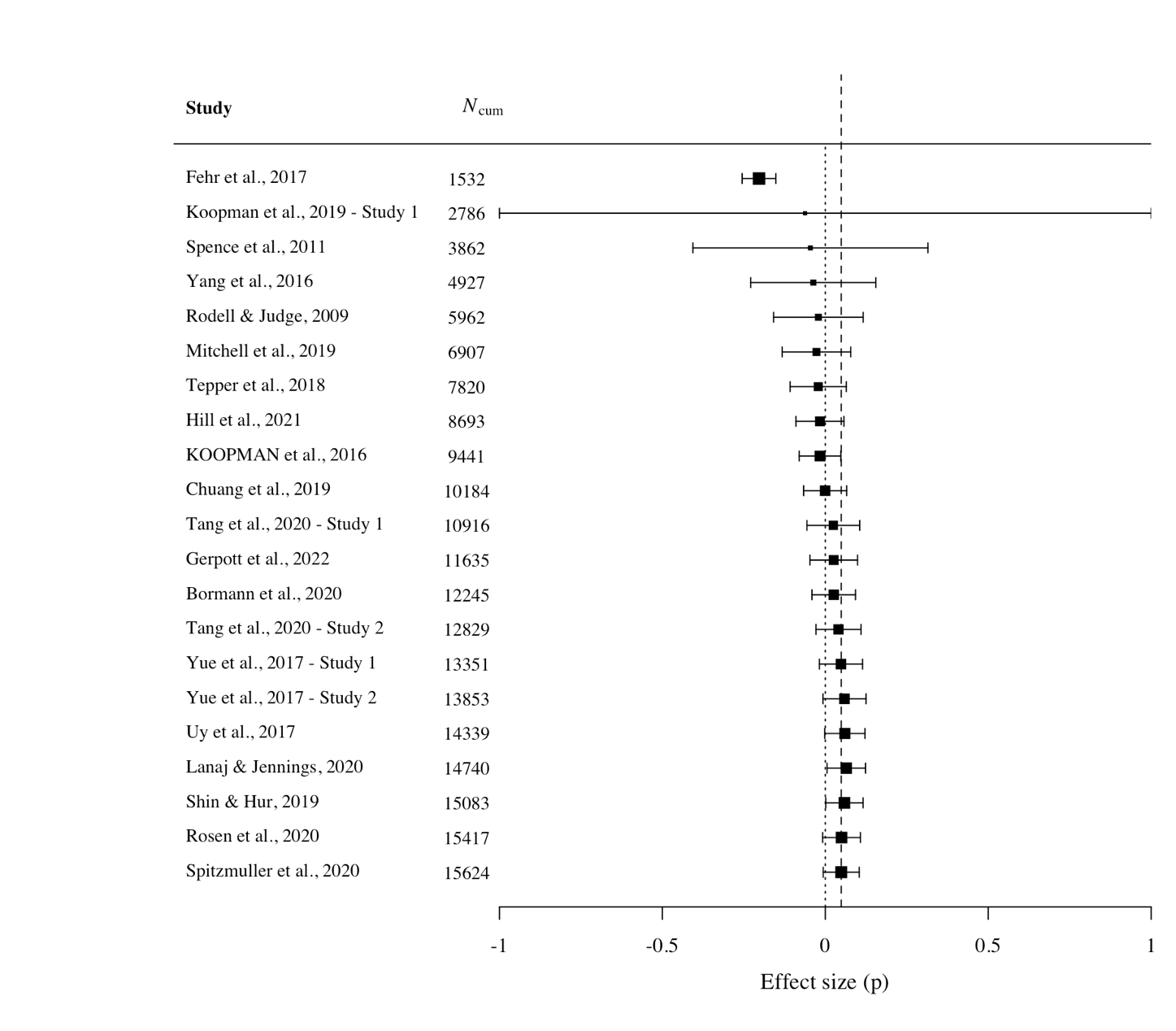 |
| --- |
| Figure S10b. CMA Plot for NA ↔ OCBs (Within) |

| 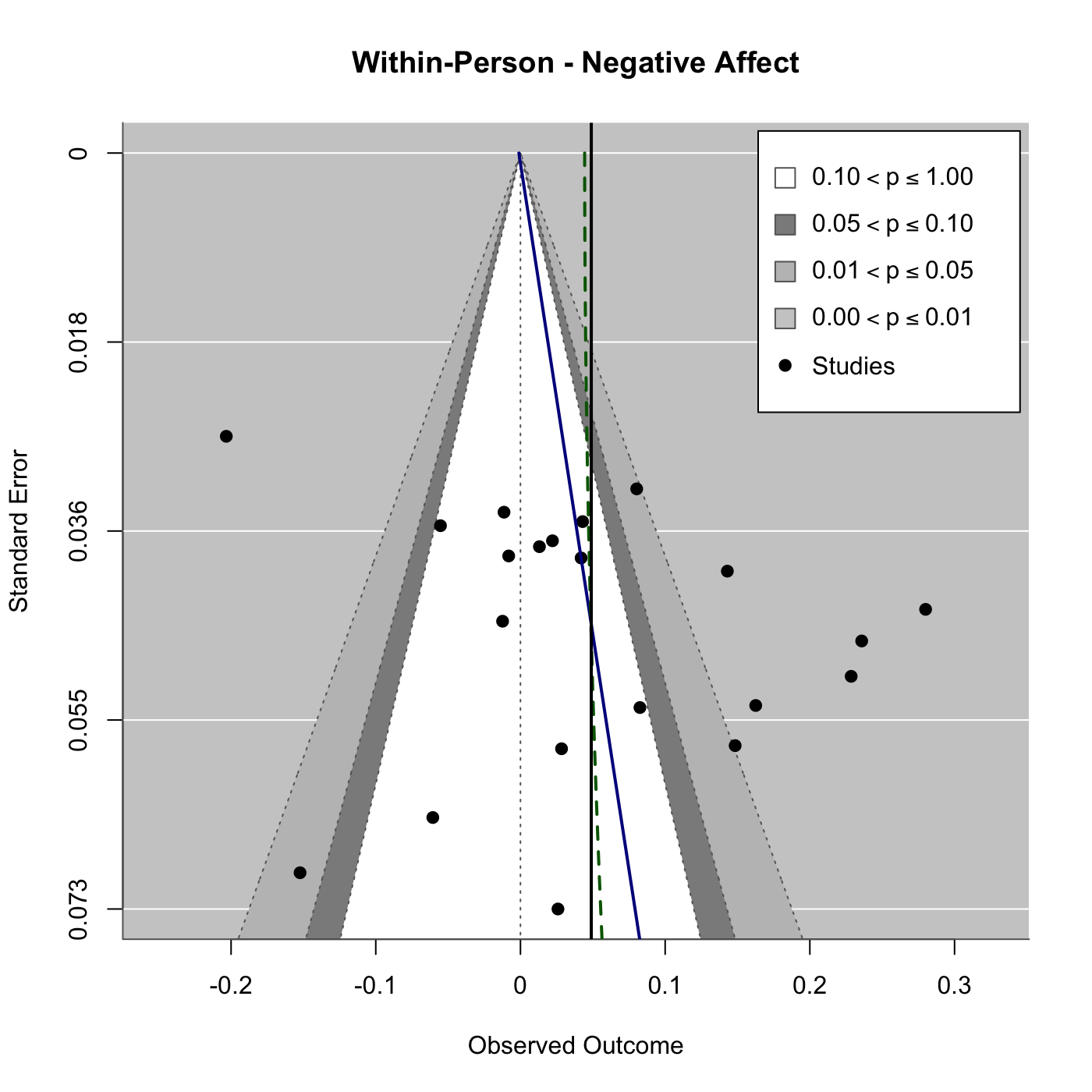 |
| --- |
| Figure S10c. Funnel Plot for NA ↔ OCBs for within-person analysis with PET (dark blue, solid), PEESE (dark green, dashed) effect size (black, solid) lines, and estimated confidence 95% CI of effect size |

| 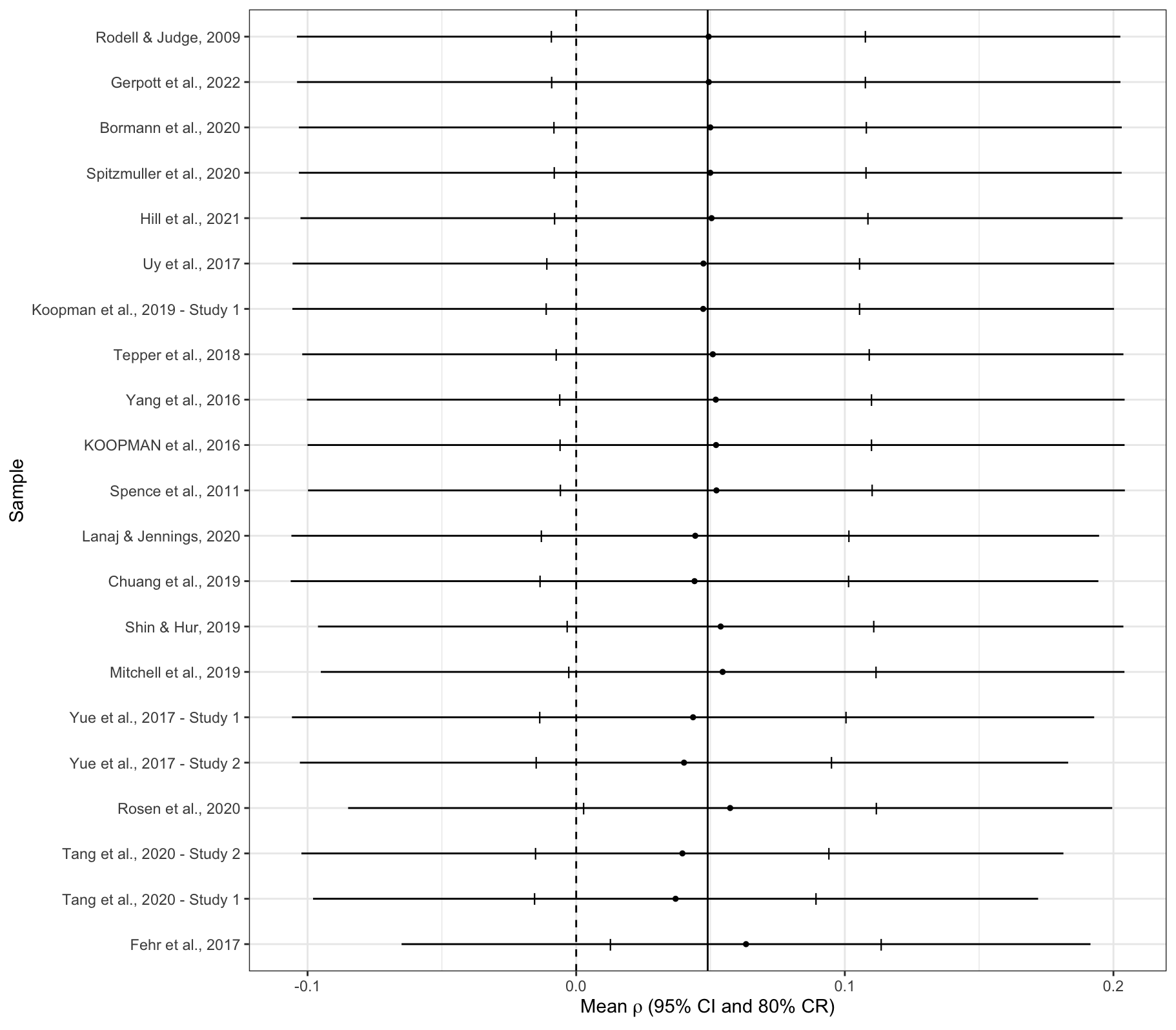 |
| --- |
| Figure S10d. Leave-One-Out Visualization for NA ↔ OCBs (Within) |

#

# Comparison of Between-Person Effects using Cross-Sectional, Intensive Longitudinal, and Longitudinal Data

As noted in the main manuscript, for the between-person analysis, correlations from cross-sectional, intensive longitudinal design, and longitudinal studies were used. In longitudinal studies, each repeated measure of a construct (e.g., Life Satisfaction – Time 1, Life Satisfaction – Time 2) was treated as a separate measure of that construct and collapsed. In intensive longitudinal studies, aggregated within-person correlations (i.e., between-person correlations calculated using within-person data) were used for the between-person analysis, which has been shown to be relatively equivalent to ‘true’ between-person effects (e.g., Ilies & Judge, 2004). This supplemental analysis aims to compare the effects derived from these different types of correlations.

As displayed in Table S2, the correlations between PA and OCB revealed that the correlation for cross-sectional data (ρ = .34) was not significantly different from that for intensive longitudinal data (ρ = .32, Δ = .02, z = .43, p = .67), nor from that for longitudinal data (ρ = .35, Δ = -.01, z = -.22, p = .83). Similarly, the difference between the correlation for intensive longitudinal data and longitudinal data was not significant (Δ = -.03, z = -.49, p = .63).

In contrast, the correlations between NA and OCB showed significant differences. The correlation for cross-sectional data (ρ = -.14) was significantly lower than that for intensive longitudinal data (ρ = .09, Δ = -.23, z = -3.54, p < .01). However, there was no significant difference between the correlations for cross-sectional data and longitudinal data (ρ = -.14 for both, Δ = -.003, z = -.09, p = .93). The correlation for intensive longitudinal data was significantly higher than that for longitudinal data (Δ = .23, z = 3.35, p < .01). The 95% confidence intervals for these differences supported these findings, with the intervals not overlapping zero for the significant comparisons, reinforcing the presence of substantial differences between the correlation estimates from different data types.

| Table S2. Meta-Analytic Cross-Sectional Effect Sizes between Subjective Well-Being and Organizational Citizenship Behaviors at the Between-Person Level using Between, Fake Between, and Longitudinal Data | | | | | | | | | |
| --- | --- | --- | --- | --- | --- | --- | --- | --- | --- |
|  |  |  |  |  |  | 95% CI | | 80% CR | |
|  | k | *r* | SD_r_ | *ρ* | SD*_ρ_* | LL | UL | LL | UL |
| Life Satisfaction |  |  |  |  |  |  |  |  |  |
| *Cross-Sectional* | 26 | .26 | .16 | .31 | .18 | .24 | .38 | .10 | .52 |
| *Intensive Longitudinal* | 2 | .44 | .07 | .46 | .05 | -.01 | .93 | .46 | .46 |
| *Longitudinal* | 6 | .18 | .15 | .21 | .16 | .04 | .38 | .00 | .42 |
| Positive Affect |  |  |  |  |  |  |  |  |  |
| *Cross-Sectional* | 90 | .29 | .15 | .34 | .17 | .30 | .38 | .14 | .54 |
| *Intensive Longitudinal* | 23 | .28 | .18 | .32 | .22 | .22 | .41 | .08 | .56 |
| *Longitudinal* | 14 | .31 | .16 | .35 | .19 | .24 | .46 | .13 | .57 |
| Negative Affect |  |  |  |  |  |  |  |  |  |
| *Cross-Sectional* | 77 | -.12 | .14 | -.14 | .17 | -.18 | -.10 | -.33 | .05 |
| *Intensive Longitudinal* | 20 | .07 | .24 | .09 | .28 | -.04 | .22 | -.23 | .41 |
| *Longitudinal* | 21 | -.12 | .11 | -.14 | .12 | -.19 | -.08 | -.25 | -.02 |
| *Note.* k = number of studies, *r* = mean correlation coefficient, SD_r_ = standard deviation of *r*, *ρ* = mean corrected correlation, SD*_ρ_* = standard deviation of *ρ*, LL = lower limit, UL = upper limit, 95% CI = 95% confidence interval, and 80% CR = 80% credibility interval. | | | | | | | | | |

# Comparison Between Sample Size vs. Responses for Within-Person Analysis

| Table S3. Meta-Analytic Cross-Sectional Effect Sizes between Subjective Well-Being and Organizational Citizenship Behaviors at the Between-Person Level using Between, Fake Between, and Longitudinal Data | | | | | | | | | |
| --- | --- | --- | --- | --- | --- | --- | --- | --- | --- |
|  |  |  |  |  |  | 95% CI | | 80% CR | |
|  | Weight | *r* | SD_r_ | *ρ* | SD*_ρ_* | LL | UL | LL | UL |
| Positive Affect |  |  |  |  |  |  |  |  |  |
| *Daily Reponses* | 15435 | .16 | .11 | .18 | .13 | .13 | .24 | .02 | .34 |
| *Sample Size* | 2030 | .16 | .12 | .19 | .14 | .13 | .24 | .10 | .27 |
| Negative Affect |  |  |  |  |  |  |  |  |  |
| *Daily Reponses* | 15624 | .04 | .11 | .05 | .12 | -.01 | .10 | -.10 | .20 |
| *Sample Size* | 1872 | .03 | .11 | .04 | .13 | -.02 | .10 | .01 | .06 |
| *Note.* Weight = either the daily responses or sample size, *r* = mean correlation coefficient, SD_r_ = standard deviation of *r*, *ρ* = mean corrected correlation, SD*_ρ_* = standard deviation of *ρ*, LL = lower limit, UL = upper limit, 95% CI = 95% confidence interval, and 80% CR = 80% credibility interval. | | | | | | | | | |
